# Synthesis of trifluoromethylated 2*H*-azirines through Togni reagent-mediated trifluoromethylation followed by PhIO-mediated azirination

**DOI:** 10.3762/bjoc.14.123

**Published:** 2018-06-15

**Authors:** Jiyun Sun, Xiaohua Zhen, Huaibin Ge, Guangtao Zhang, Xuechan An, Yunfei Du

**Affiliations:** 1 Tianjin Key Laboratory for Modern Drug Delivery and High-Efficiency, School of Pharmaceutical Science and Technology, Tianjin University, Tianjin 300072, China; 2 Collaborative Innovation Center of Chemical Science and Engineering (Tianjin), Tianjin 300072, China

**Keywords:** azirination, 2*H*-azirine, iodosobenzene, Togni reagent, β-trifluoromethylation

## Abstract

The reaction of enamine compounds with the Togni reagent in the presence of CuI afforded β-trifluoromethylated enamine intermediates, which were converted directly to biologically interesting trifluoromethylated 2*H*-azirines by an iodosobenzene (PhIO)-mediated intramolecular azirination in a one-pot process.

## Introduction

**Figure 1:**
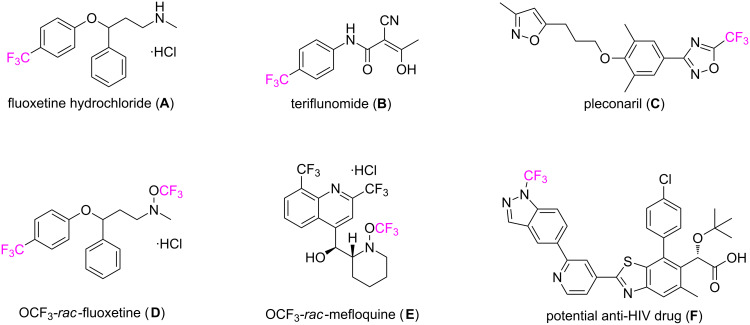
Representative pharmaceutical agents bearing the CF_3_ group.

The trifluoromethyl group is a striking structural motif, which can be widely found in the fields of pharmaceutical and agrochemical sciences. The introduction of this functional group in drug molecules can enhance their chemical and metabolic stability, improve their lipophilicity and bioavailability, and increase protein-binding affinity [1–6]. In this regard, the CF_3_ group has been introduced into many pharmaceutical agents [7–16]. For example, fluoxetine hydrochloride (Figure 1, **A**) [4,9–10] (Prozac^®^, an antidepressant and a selective serotonin reuptake inhibitor for the treatment of major depressive disorders, obsessive–compulsive disorders, etc.), teriflunomide (Figure 1, **B**) [11–13] (Aubagio^®^, the active metabolite of leflunomide for the treatment of multiple sclerosis), and pleconaril (Figure 1, **C**) [14–16] (an antiviral drug), all possess this privileged substituent. Although many useful synthetic methods [17–21] have been established for introducing the CF_3_ group into various organic molecules, the further development of novel routes for the selective trifluoromethylation is of continuing interest for synthetic and medicinal chemists.

**Figure 2:**
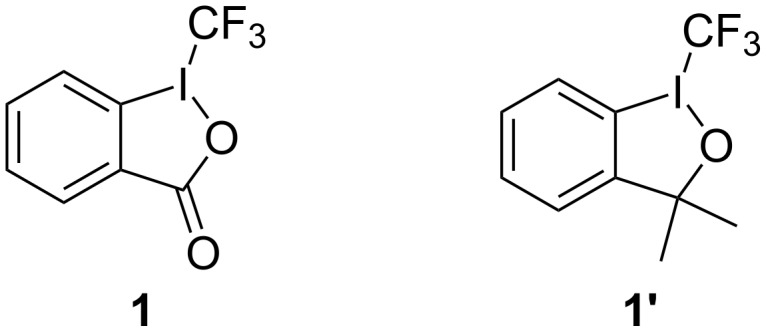
The structures of the Togni reagents 1-(trifluoromethyl)-1,2-benziodoxol-3(1*H*)-one (**1**) and trifluoromethyl-1,3-dihydro-3,3-dimethyl-1,2-benziodoxole (**1’**).

Togni reagents, including 1-(trifluoromethyl)-1,2-benziodoxol-3(1*H*)-one (**1**) and trifluoromethyl-1,3-dihydro-3,3-dimethyl-1,2-benziodoxole (**1’,** Figure 2), are effective and efficient hypervalent iodine reagents for trifluoromethylation reactions of a variety of substrates [22–23]. These reagents have found wide applications in the area of organofluorine chemistry, synthetic method development as well as medicinal chemistry [24–40]. For example, the Togni reagents have been successfully applied to introduce the CF_3_ group into pharmaceutical agents such as the fluoxetine derivative **D** (Figure 1), the mefloquine derivative **E** [41] and compound **F** [42] – a potential anti-HIV drug bearing a NCF_3_ moiety.

**Scheme 1:**
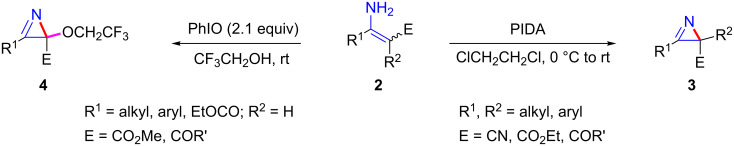
Our previous hypervalent iodine-mediated synthesis of 2*H*-azirine compounds.

2*H*-Azirines are a class of highly strained and reactive molecules containing a C–N double bond. The exclusive framework can be found in some natural products [43–47], which were shown to possess antibiotic activities [43–44]. Furthermore, compounds with this structural motif are also useful building blocks for the synthesis of functionalized amino derivatives and N-containing heterocyclic derivatives [48–51]. Thus, this class of compounds has gained considerable attention from synthetic chemists and many useful synthetic approaches [52–55] have been developed for accessing this exclusive class of heterocycles. In our previous works, we have realized the application of hypervalent iodine reagents for the construction of the 2*H*-azirine skeleton starting from enamines **2** via intramolecular oxidative cyclization (Scheme 1) [56–57]. When the R^2^ substituent is alkyl or aryl, the corresponding substrates **2** were converted to a series of alkylated or arylated 2*H*-azirines **3** in the presence of phenyliodine diacetate (PIDA) in 1,2-dichloroethane (DCE) [56]. Alternatively, the treatment of β-unsubstituted enamine substrates (**2**, R^2^ = H) with PhIO in 2,2,2-trifluoroethanol (TFE) afforded 2-trifluoroethoxy-2*H*-azirines **4** [57]. The latter process involves an intermolecular oxidative trifluoroethoxylation and the subsequent oxidative intramolecular azirination. In continuation of our interest in the construction of the 2*H*-azirine skeleton bearing versatile substituents, we herein report that the biologically interesting CF_3_ group can be incorporated into the privileged 2*H*-azirine framework through the Togni reagent **1**-mediated trifluoromethylation followed by PhIO-mediated azirination in a one-pot process.

## Results and Discussion

**Scheme 2:**
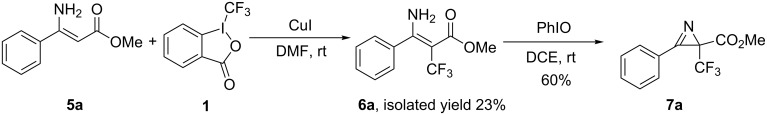
Study on the presumed Togni reagent **1**-mediated trifluoromethylation followed by PhIO-mediated azirination.

It is well documented that Togni reagents can realize the direct trifluoromethylation of alkenes [58–60] and electron-rich enamides [61]. Inspired by this, we envisaged that Togni reagent **1** could also enable the introduction of a CF_3_ group to the β-position of enamine substrates, and the so-obtained trifluoromethylated enamines could undergo a hypervalent iodine-mediated intramolecular azirination to give the corresponding trifluoromethylated 2*H*-azirines [56–57]. To test this conversion, the readily available enamine **5a** was used as a model substrate. The treatment of **5a** with Togni reagent **1** in the presence of CuI in *N*,*N*-dimethylformamide (DMF) [62] at room temperature for two hours afforded the β*-*trifluoromethylated enamine **6a** in a 23% yield. Subjecting enamine **6a** to PhIO in 1,2-dichloroethane (DCE) for 12 hours at room temperature led to the formation of the desired β*-*trifluoromethylated 2*H*-azirine **7a** in a yield of 60% (Scheme 2).

In order to make the synthesis of β*-*trifluoromethylated 2*H*-azirine more concise and convenient, we were keen to probe whether the two-step synthesis could be combined into a one-pot process. For this purpose, we first carried out the reaction of Togni reagent **1** and **5a** in the presence of CuI in DMF at room temperature, followed by an addition of PhIO. However, only trace amounts of the expected product **7a** were obtained (Table 1, entry 1). We next screened various solvents to increase the reaction outcome (Table 1, entries 1–4). Judging by the yield of the desired product, it was concluded that DCE was the best solvent (Table 1, entry 3). By increasing the reaction temperature from rt to 60 °C, the yields significantly increased to 55% (Table 1, entries 3, 5 and 6). However, an attempt to improve the product yield by operating the reaction at a higher temperature was unsuccessful (Table 1, entry 7). Replacing the catalyst CuI with other commonly used copper catalysts including CuCl, CuBr and CuOAc led to a decreased yield in each case (Table 1, entries 8–10). In addition the other commonly employed hypervalent iodine(III) reagents, namely, PIDA and phenyliodine bis(trifluoroacetate) (PIFA) were tested, but the results indicated that they were ineffective to further improve the yields (Table 1, entries 11 and 12).

**Table 1:** Optimization of reaction conditions.^a^

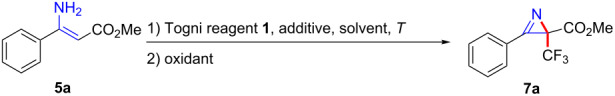					

Entry	Catalyst	Oxidant^b^	Solvent	Temp. (°C)	Yield^c^ (%)

1	CuI	PhIO	DMF	rt	trace
2	CuI	PhIO	CH_3_CN	rt	13
3	CuI	PhIO	DCE	rt	24
4	CuI	PhIO	toluene	rt	12
5	CuI	PhIO	DCE	40	35
6	CuI	PhIO	DCE	60	55
7	CuI	PhIO	DCE	reflux	48
8	CuCl	PhIO	DCE	60	49
9	CuBr	PhIO	DCE	60	50
10	CuOAc	PhIO	DCE	60	38
11	CuI	PIDA	DCE	60	46
12	CuI	PIFA	DCE	60	30

^a^Reaction conditions: Togni reagent **1** (1.2 mmol), **5a** (1.0 mmol), catalyst (0.2 mmol), oxidant (1.5 mmol) in solvent (10 mL) unless otherwise stated. ^b^The oxidant was added to the reaction mixture after the substrate **5a** was completely consumed (TLC analysis). ^c^Isolated yield.

**Scheme 3:**
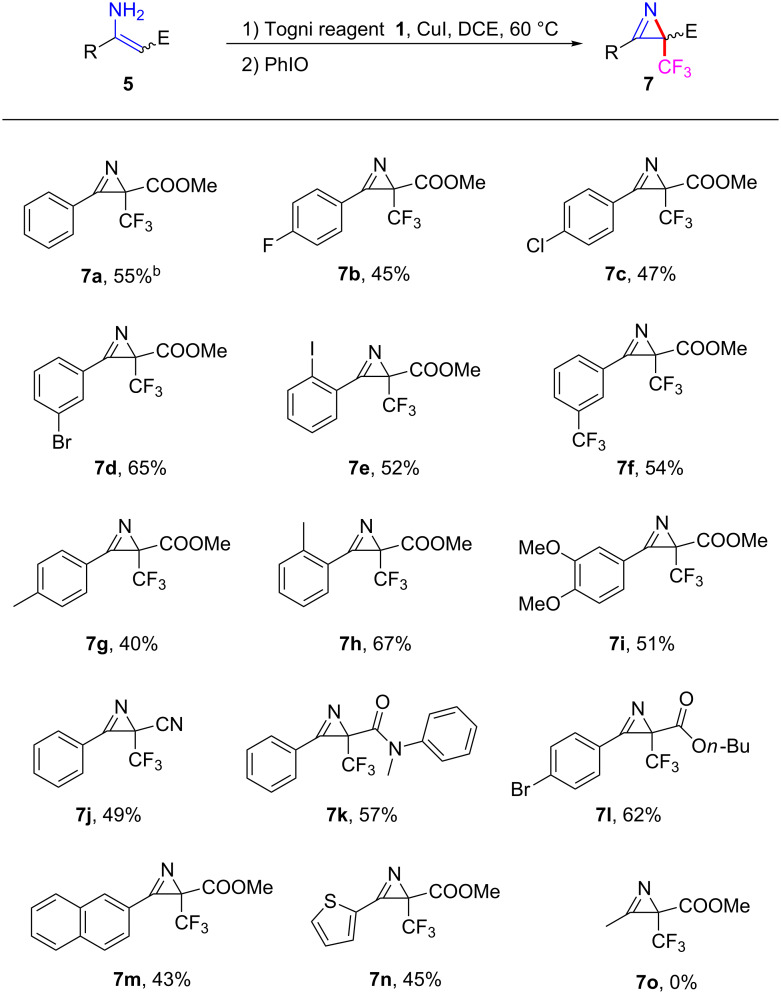
Togni reagent/PhIO-mediated one-pot synthesis of β-trifluoromethyl 2*H*-azirines. Reaction conditions: **1** (1.2 mmol), **5** (1.0 mmol), CuI (0.2 mmol), PhIO (1.5 mmol) in DCE (10 mL) unless otherwise stated. PhIO was added to the reaction mixture after the substrate **5** was completely consumed (TLC analysis). Yields refer to isolated yields.

With the optimized conditions in hand, we next explored the substrate scope for this newly established one-pot oxidative trifluoromethylation and azirination reaction. As shown in Scheme 3, a variety of substrates bearing halogen substituents at the *ortho*, *meta* and *para*-positions of the phenyl ring in the substrates were converted to the expected 2*H*-azirines **7b**–**e** in 45–65% one-pot yield. Notably, the substrate having a trifluoromethyl group at the *meta*-position in the phenyl ring also afforded the desired 2*H*-azirine product **7f** bearing two CF_3_ substituents in a satisfactory one-pot yield. Various enamine substrates with electron-donating groups (*p*-Me, *o*-Me and 3,4-di*-*OMe) in the aryl ring, also reacted efficiently under the conditions of the one-pot process to afford the corresponding products **7g**–**i** in a yield of 40–67%. Furthermore, when replacing the methoxycarbonyl group in **5a** with a cyano or *N*-methyl-*N*-phenylformyl group, the corresponding substrates **5j** and **5k** were converted to the desired products **7j** and **7k** in a yield of 49% and 57%, respectively. The methoxy group in the ester moiety could also be replaced by the *n*-butoxy group, with the desired product **7l** being isolated in a yield of 62%. In addition, this method was also applicable to substrates bearing naphthyl or thienyl groups at R substitution to give the desired products **7m** and **7n** in a yield of 43% and 45%, respectively. However, the method was not applicable to the substrate bearing an alkyl group, as the reaction of **5o**, even at lower temperatures (−20 °C, 0 °C, 20 °C and 40 °C) gave a complex mixture after adding PhIO.

**Scheme 4:**
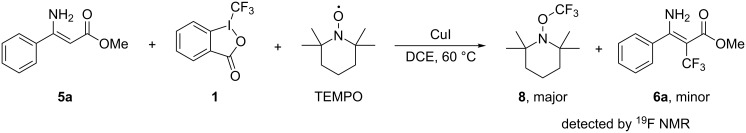
Control study with TEMPO.

To gain further insights into the reaction mechanism, 2,2,6,6-tetramethyl-1-piperidinyloxy (TEMPO), a well-known radical scavenger, was introduced to the model reaction (Scheme 4) following the method previously reported in the literature [63]. It was found that the trifluoromethylation was hampered and the TEMPO-CF_3_ adduct **8** was formed as a major product based on the analysis of its ^19^F NMR (δ −55.67).

**Scheme 5:**
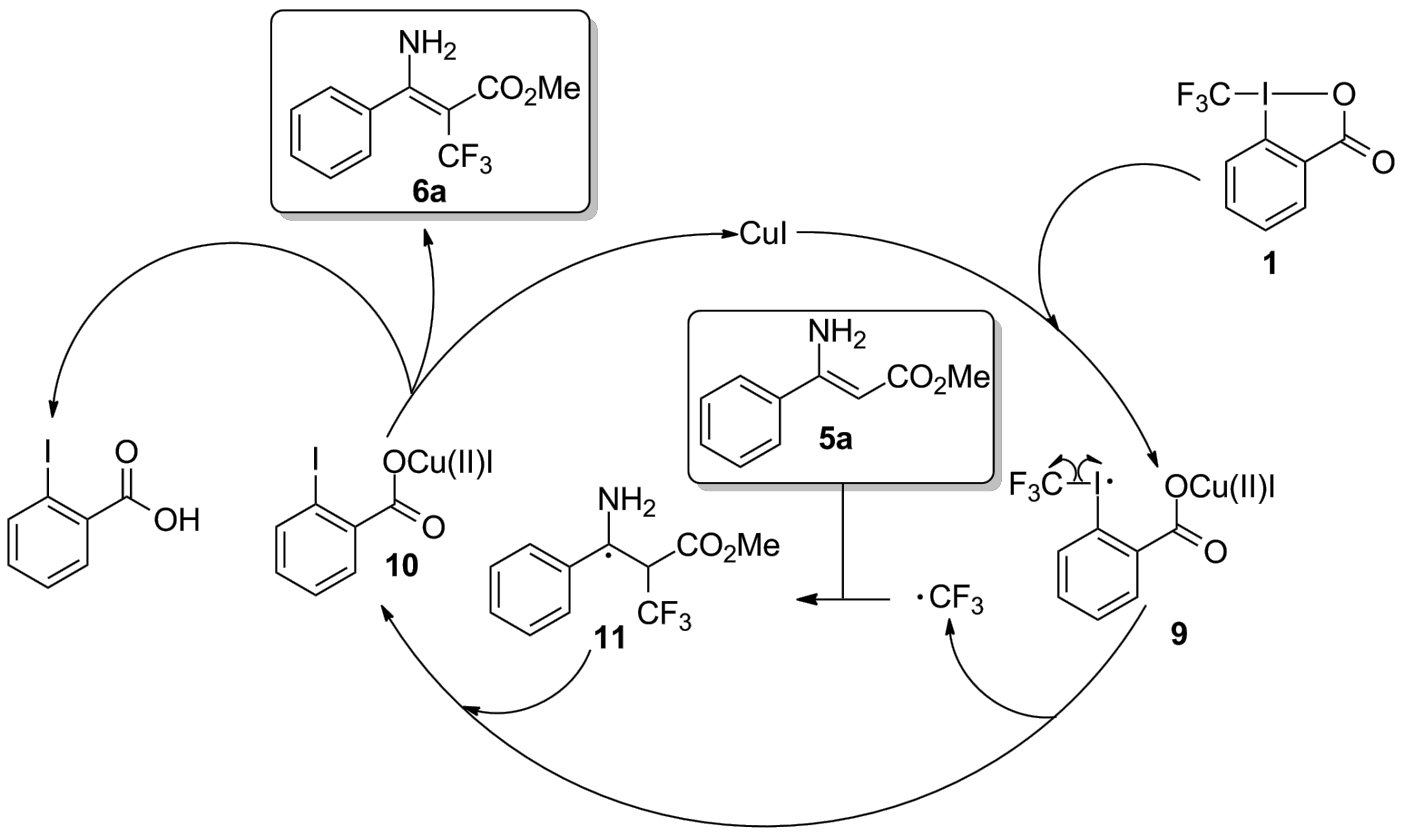
Proposed mechanism for the Togni reagent-mediated trifluoromethylation of enamines.

The above results from the experiment provided supportive evidence that the CF_3_ radical was likely involved as a reactive species in the reaction process. Based on this and previous reports [62–68], a possible reaction pathway has been proposed and is outlined in Scheme 5. Initially, CuI catalytically activates the Togni reagent **1**, leading to the formation of the CF_3_-containing radical intermediate **9**. Decomposition of the intermediate **9** produces (2-iodobenzoyloxy)copper(II) iodide (**10**) [65–66] with the simultaneous release of a CF_3_ radical. Then, the reaction of enamine **5a** with the CF_3_ radical affords the carbon-centered radical **11**. Next, the reaction of **10** and **11**, possibly through an electron-transfer process, along with the conversion of intermediate **10** to 2-iodobenzoic acid enables the conversion of intermediate **11** to **6a** (possibly tautomerized from its imine isomer) [69]. Finally, the β-trifluoromethylated enamine **6a** undergoes intramolecular azirination affording the corresponding β-trifluoromethylated 2*H*-azirine via a known pathway [56–57].

## Conclusion

In summary, we have reported an efficient hypervalent iodine-mediated trifluoromethylation and azirination process. In this transformation, the introduction of the CF_3_ group to the β-position of enamines followed by the intramolecular azirination was realized in a one-pot process, providing a general and straightforward access to biologically interesting trifluoromethylated 2*H*-azirine compounds. This method features mild reaction conditions, a simple operation, and metal-free characteristics. The presence of both, the biologically interesting CF_3_ group and the 2*H*-azirine skeleton in the products obtained might making them interesting for further applications in biological studies.

## Supporting Information

File 1 Synthetic details and characterization data.
