# Supplementary material for: Synthesis of trifluoromethylated 2H-azirines through Togni reagent-mediated trifluoromethylation followed by PhIO-mediated azirination
Source: Beilstein J Org Chem. 2018 Jun 15;14:1452–8. doi: 10.3762/bjoc.14.123 (PMC6009194; doi:10.3762/bjoc.14.123)
Supplement: File 1 — Synthetic details and characterization data. [file Beilstein_J_Org_Chem-14-1452-s001.pdf]

Supporting Information

for

Synthesis of trifluoromethylated 2*H*-azirines through Togni reagent-mediated trifluoromethylation followed by PhIO-mediated azirination

Jiyun Sun[1], Xiaohua Zhen[1], Huaibin Ge[1], Guangtao Zhang[1], Xuechan An[1], and Yunfei Du[1,2,*]

Address: [1]Tianjin Key Laboratory for Modern Drug Delivery and High-Efficiency, School of Pharmaceutical Science and Technology, Tianjin University, Tianjin 300072, China and [2]Collaborative Innovation Center of Chemical Science and Engineering (Tianjin), Tianjin 300072, China

Email: Yunfei Du - duyunfeier@tju.edu.cn; Tel: +86-22-27406121.

*Corresponding author

Synthetic details and characterization data

Contents

General Information S2
Preparation of Togni reagent **1** and substrates **5** S2
Preparation of β-trifluoromethylated 2*H*-azirines **7** and **6a** S6
Procedure of control experiment S6
References S7
Characterization data of products **7** and intermediate **6a** S8
^{1}H NMR, ^{19}F NMR and ^{13}C NMR spectra of **7** and intermediate **6a** S14

General information

^{1}H, ^{19}F and ^{13}C NMR spectra were recorded using a 600 MHz spectrometer (564 MHz for ^{19}F NMR and 150 MHz for ^{13}C NMR) or a 400 MHz spectrometer (375 MHz for ^{19}F NMR and 100 MHz for ^{13}C NMR) at 25 °C. Chemical shifts values are given in ppm and referred to TMS (0.00 ppm) as internal standard. Data are reported as follows: chemical shift, multiplicity (s = singlet, d = doublet, t = triplet, q = quartet, br = broad, m = multiplet). The coupling constants *J*, are reported in hertz (Hz). High resolution mass spectrometry (HRMS) was obtained on a Q-TOF micro spectrometer. Melting points were determined with a Micromelting point apparatus. TLC plates were visualized by exposure to ultraviolet light. Reagents and solvents were purchased as reagent grade and were used without further purification. All reactions were performed in standard glassware, heated at 70 °C for 3 h before use. Flash column chromatography was performed using silica gel (200–300 mesh).

Preparation of Togni reagent and substrates 5

Preparation of Togni reagent

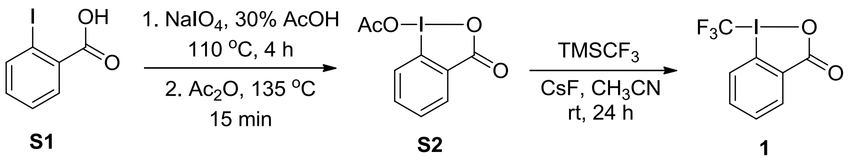

A 500 mL round-bottomed flask was charged with 2-iodobenzoic acid (20.0 g, 80.64 mmol, 1.0 equiv), finely ground $NaIO_4$ (18.11 g. 84.67 mmol, 1.05 equiv), and 30% aqueous acetic acid (120 mL). The flask was equipped with a reflux condenser and placed in an oil bath preheated to 110 °C. The reaction mixture was allowed to stir at this temperature for four hours at which point the flask was removed from the oil bath and allowed to cool to room temperature. Ice water (100 mL) was added, and the suspension was allowed to stir for 5 min. The fine white needle-like crystals were isolated by

Büchner filtration. The crystals were washed with ice water (2 × 100 mL) and dried in vacuo to afford **S1** in greater than quantitative yield (contains impurities).

In a 500 mL round-bottomed flask containing the crude 1-hydroxy-1,2-benziodoxol-3-(1*H*)-one (assumed 80.64 mmol, 100% yield for the previous step) was suspended in 56 mL Ac_2O. The flask was connected to a reflux condenser and placed in an oil bath preheated to 135 °C. The reaction mixture was stirred for 15 min until most of the solid materials dissolved. The flask was removed from the oil bath and allowed to cool to room temperature, resulting in the formation of white crystals. The flask was then placed in a −20 °C freezer overnight to complete the crystallization. The liquids were decanted and the flask was covered with aluminum foil to protect the product from light. Volatiles were removed in vacuo to afford the product in greater than quantitative yield.

To a flame-dried 500 mL round-bottomed flask containing the unpurified 1-acetoxy-1,2-benziodoxol-3-(1*H*)-one (assumed 80.64 mmol, 100% yield for the previous step) under argon was added anhydrous CsF (0.302 g, 2.0 mmol, 0.025 equiv), CH_3CN (170 mL), and $TMSCF_3$ (15.93 g, 16.6 mL, 112 mmol, 1.4 equiv) successively. The reaction mixture was stirred vigorously for 24 h. The white solid suspended in the reaction mixture was then isolated by Büchner filtration. This solid was dissolved in chloroform (300 mL) and any insoluble material was removed by filtration through a cotton plug. The colorless filtrate was then washed with water (200 mL) and sat. aq. Na_2CO_3 (200 mL). The solution was dried over Na_2SO_4, filtered and concentrated in vacuo to afford pure **1** (11.07 g, 43%). A second batch of **1** was obtained by concentrating the brown reaction filtrate, dissolving in chloroform (200 mL), washing with water (100 mL) and $NaHCO_3$ (100 mL). The solution was dried over Na_2SO_4 and concentrated in vacuo to afford a brownish-white solid. The solid was washed quickly in the flask with a small amount of methanol (~20 mL) to remove a large amount of the brown impurity. The resulting solid was then recrystallized from boiling methanol. Cooling to room temperature and placing in a −20 °C freezer resulted in crystal formation. The crystals were collected, washed with cold methanol, and dried in vacuo to provide a second batch of pure **1** (2.58 g, 10% yield). Total yield: 13.65 g (53%).

Preparation of substrates 5a–o

General procedure 1:

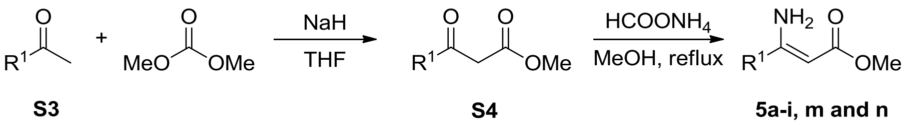

To a solution of ketone **S3** (1.0 equiv, 20 mmol) in THF (80 mL) was added methyl dicarbonate (3.0 equiv, 60 mmol) and NaH (2.0 equiv, 40 mmol, 60%). The reaction mixture was refluxed until TLC indicated the total consumption of the ketone. After cooling, the reaction mixture was poured into ice-water (100 mL), acidified with aqueous HCl (3 M) to pH 2~3 and extracted with EA (100 mL × 3). The combined organic layer was dried over Na_2SO_4 and evaporated under reduced pressure.

The obtained β-keto ester **S4** (1.0 equiv, 15 mmol) was dissolved in absolute MeOH (60 mL), followed by the addition of ammonium formate (5.0 equiv, 75 mmol). The reaction mixture was stirred under reflux until TLC indicated the total consumption of the β-keto ester and filtered through a short pad of Celite. The filtrate was concentrated in vacuo. To the residue was added water (100 mL), then EA (100 mL × 3) was used to extract the mixture and the organic layer was combined, dried over Na_2SO_4 and evaporated to dryness. The desired pure product was obtained by silica gel chromatography using a mixture of PE and EA as eluent.

Subtrates **5j** and **5o** were prepared from the commercial 3-oxo-3-phenylpropanenitrile **S4-j** and methyl 3-oxobutanoate **S4-o**.

General procedure 2:

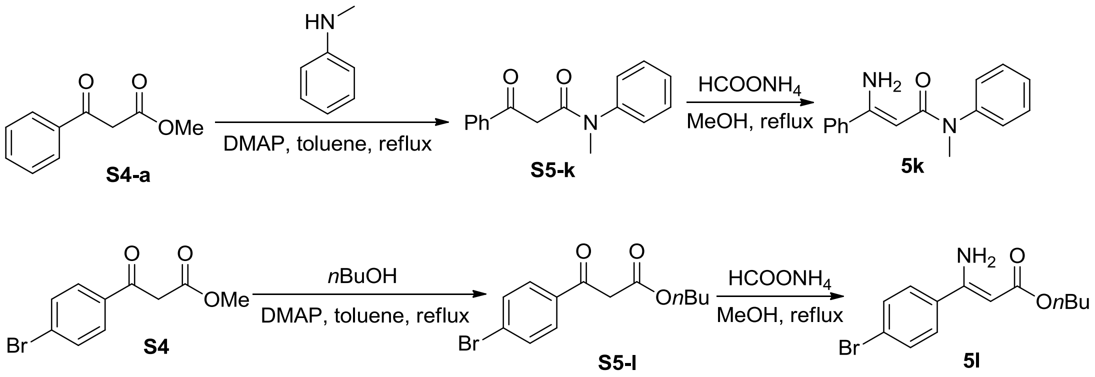

The obtained β-keto ester **S4** (1.0 equiv, 15 mmol) was dissolved in toluene. To this solution was added DMAP (4-dimethylaminopyridine, 0.1 equiv, 1.5 mmol) and *N*-methylaniline (1.1 equiv, 16.5 mmol, for **S5-k**) or *n*-butanol (1.1 equiv, 16.5 mmol, for **S5-l**) at room temperature. The reaction mixture was heated to reflux until **S4** was comsumed completely. The reaction mixture was cooled to room temperature and the solvent was removed in vacuo. To the residue was added water (100 mL), then EA (100 mL × 3) was used to extract the mixture and the organic layer was combined, dried over Na_2SO_4 and evaporated to dryness. The desired pure product, **S5-k** or **S5-l** were obtained by silica gel chromatography using a mixture of PE and EA as eluent.

The obtained **S5-k** or **S5-l** (1.0 equiv, 10 mmol) was disosolved in absolute MeOH (40 mL), followed by the addition of ammonium formate (50 mmol). The reaction mixture was stirred under reflux until TLC indicated the total consumption of the substrate and filtered through a short pad of Celite. The filtrate was concentrated in vacuo. To the residue was added water (100 mL), then EA (100 mL × 3) was used to extract the mixture and the organic layer was combined, dried over Na_2SO_4 and evaporated to dryness. The desired pure product **5k** or **5l** was obtained by silica gel chromatography using a mixture of PE and EA as eluent.

Spectral properties of the substrates **5**[1-2] and Togni reagent[3-4] matched the previously reported values.

Preparation of β-trifluoromethylated 2*H*-azirines 7 and intermediate 6a

General procedure

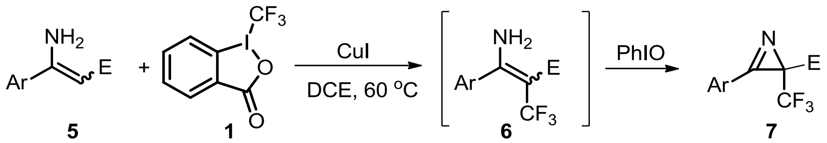

5 **1** **6** **7**

To the enamine substrate **5** (1.0 mmol) dissolved in DCE (10 mL) were added at room temperature Togni reagent **1** (1.2 mmol) and CuI (0.2 mmol). The reaction mixture was heated to 60 °C and progress of the reaction was monitored by TLC. Upon completion, PhIO (1.5 mmol) was added to the mixture and the reaction temperature was maintained at 60 °C. When TLC analysis indicated total comsumption of the formed intermediate **6**, the reaction was cooled to room temperature and quenched with saturated aqueous $NaHCO_3$ (30 mL). Then DCM (20 mL × 3) was used to extract the mixture and the combined organic layer was washed with saturated aqueous NaCl (20 mL), dried over Na_2SO_4 and evaporated to dryness. The desired product **7** was obtained by silica gel chromatography using a mixture of EtOAc/petroleum ether (PE) (10/90, v/v) as eluent.

The procedure of control experiments

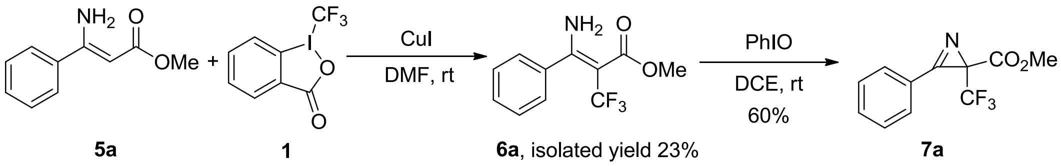

5a **1** **6a**, isolated yield 23% **7a**

Procedure of step I:

The enamine substrate **5a** (1.0 mmol) was dissolved in DMF (10 mL), then Togni reagent **1** (1.2 mmol) and CuI (0.2 mmol) were added at room temperature. The progress of the reaction was monitored by TLC. Upon completion, the reaction was

quenched with saturated aqueous $NaHCO_3$ (30 mL) at room temperature. Then DCM (20 mL × 3) was used to extract the mixture and the combined organic layer was washed with saturated aqueous NaCl (20 mL), dried over Na_2SO_4 and evaporated to dryness. The desired product **6a** was obtained by silica gel chromatography using a mixture of EtOAc/petroleum ether (PE) (10/90, v/v) as eluent.

Procedure of Step II:

The obtained **6a** (56 mg, 0.23 mmol) was dissolved in DCE (10 mL), then PhIO (1.2 equiv, 0.28 mmol) was added to the reaction mixture at room temperature. When the TLC analysis indicated intermediate **6a** was totally consumed, the reaction was quenched with saturated aqueous $NaHCO_3$ (10 mL) at room temperature. Then DCM (10 mL × 3) was used to extract the mixture and the organic layer was combined, washed with saturated aqueous NaCl (10 mL), dried over Na_2SO_4 and evaporated to dryness. The desired product **7a** was obtained by silica gel chromatography using a mixture of EtOAc/petroleum ether (PE) (10/90, v/v) as eluent. Yield: 33.5 mg, 60%.

References

1. Sun, J.; Zhang-Negrerie, Daisy.; Du, Y. *Adv. Synth. Catal.* **2016**, *358*, 2035.

2. Yuan, Y.; Hou, W.; Zhang-Negrerie, D.; Zhao, Kang.; Du, Y. *Org. Lett.* **2014**, *16*, 5410.

3. Stanek, K.; Koller, R.; Togni, A. *J. Org. Chem.* **2008**, *73*, 7678.

4. Eisenberger, P.; Gischig, S.; Togni, A. *Chem. – Eur. J.* **2006**, *12*, 2579.

Characterization data of products 7 and intermediate 6a

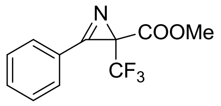

7a

Methyl 3-phenyl-2-(trifluoromethyl)-2*H*-azirine-2-carboxylate (7a)

Following the general procedure, **7a** was purified by silica gel chromatography (EtOAc/PE = 10/90). Yield: 134 mg, 55%, yellow oil. ^{1}H NMR (600 MHz, $CDCl_3$) δ 7.92 (d, *J* = 7.1 Hz, 2H), 7.73 (t, *J* = 7.5 Hz, 1H), 7.64 (t, *J* = 7.7 Hz, 2H), 3.80 (s, 3H). ^{19}F NMR (564 MHz, $CDCl_3$) δ -65.70. ^{13}C NMR (150 MHz, $CDCl_3$) δ 166.6, 155.5, 135.2, 131.1, 129.7, 122.78 (q, *J* = 275.3 Hz), 119.6, 53.2, 38.0 (q, *J* = 38.9 Hz). HRMS (ESI) calcd for $C_{11}H_8F_3NNaO_2^+$ [M + Na^+] 266.0399, found 266.0403.

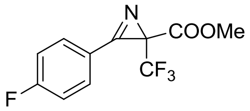

7b

Methyl 3-(4-fluorophenyl)-2-(trifluoromethyl)-2*H*-azirine-2-carboxylate (7b)

Following the general procedure, **7b** was purified by silica gel chromatography (EtOAc/PE = 10/90). Yield: 128 mg, 45%, yellow oil. ^{1}H NMR (600 MHz, $CDCl_3$) δ 8.37 – 7.69 (m, 2H), 7.34 (t, *J* = 8.5 Hz, 2H), 3.81 (s, 3H). ^{19}F NMR (564 MHz, $CDCl_3$) δ -65.76, -99.80. ^{13}C NMR (150 MHz, $CDCl_3$) δ 167.0 (d, *J* = 177.4 Hz), 165.9, 154.5, 133.7 (d, *J* = 9.8 Hz), 122.7 (q, *J* = 275.3 Hz), 117.5 (d, *J* = 22.8 Hz), 115.9 (d, *J* = 2.9 Hz), 53.2, 38.1 (q, *J* = 38.9 Hz). HRMS (ESI) calcd for $C_{11}H_7F_4NNaO_2^+$ [M + Na^+] 284.0305, found 284.0309.

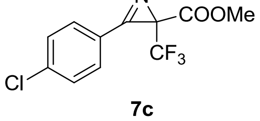

7c

Methyl 3-(4-chlorophenyl)-2-(trifluoromethyl)-2*H*-azirine-2-carboxylate (7c)

Following the general procedure, **7c** was purified by silica gel chromatography (EtOAc/PE = 10/90). Yield: 130 mg, 47%, yellow solid, m.p. 67-68 °C. ^{1}H NMR (600

MHz, $CDCl_3$) δ 7.86 (d, *J* = 8.5 Hz, 2H), 7.62 (d, *J* = 8.4 Hz, 2H), 3.81 (s, 3H). ^{19}F NMR (564 MHz, $CDCl_3$) δ -65.74. ^{13}C NMR (150 MHz, $CDCl_3$) δ 166.3, 154.9, 142.0, 132.2, 130.3, 122.6 (q, *J* = 275.4 Hz), 53.2, 38.2 (q, *J* = 39.0 Hz). HRMS (ESI) calcd for $C_{11}H_7{}^{35}ClF_3NNaO_2^+$ [M + Na$^+$] 300.0010, found 300.0018.

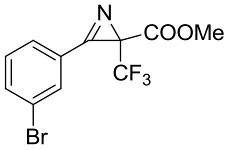

7d

Methyl 3-(3-bromophenyl)-2-(trifluoromethyl)-2*H*-azirine-2-carboxylate (7d)

Following the general procedure, **7d** was purified by silica gel chromatography (EtOAc/PE = 10/90). Yield: 209 mg, 65%, yellow oil. ^{1}H NMR (600 MHz, $CDCl_3$) δ 8.04 (s, 1H), 7.85 (d, *J* = 7.1 Hz, 2H), 7.52 (t, *J* = 7.8 Hz, 2H), 3.82 (s, 3H). ^{19}F NMR (564 MHz, $CDCl_3$) δ -65.70. ^{13}C NMR (151 MHz, $CDCl_3$) δ 166.19, 155.10, 138.10, 133.51, 131.23, 129.48, 123.71, 122.5 (q, *J* = 273.9 Hz),121.51, 53.32, 38.4 (q, *J* = 39.0 Hz). HRMS (ESI) calcd for $C_{11}H_7{}^{79}BrF_3NNaO_2^+$ [M + Na$^+$] 343.9504, found 343.9511.

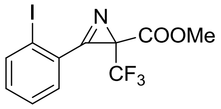

7e

Methyl 3-(2-iodophenyl)-2-(trifluoromethyl)-2*H*-azirine-2-carboxylate (7e)

Following the general procedure, **7e** was purified by silica gel chromatography (EtOAc/PE = 10/90). Yield: 192 mg, 52%, yellow solid, m.p. 65-66 °C. ^{1}H NMR (600 MHz, $CDCl_3$) δ 8.10 (d, *J* = 8.0 Hz, 1H), 7.77 (dd, *J* = 7.7, 1.5 Hz, 1H), 7.60 (td, *J* = 7.6, 0.9 Hz, 1H), 7.40 (td, *J* = 7.8, 1.6 Hz, 1H), 3.82 (s, 3H). ^{19}F NMR (564 MHz, $CDCl_3$) δ -65.67. ^{13}C NMR (151 MHz, $CDCl_3$) δ 166.5, 157.6, 141.3, 135.6, 134.7, 128.9, 124.0, 122.6 (q, *J* = 274.0 Hz), 98.8, 53.25, 39.2 (q, *J* = 38.2 Hz). HRMS (ESI) calcd for $C_{11}H_7F_3{}^{127}INNaO_2^+$ [M + Na$^+$] 391.9366, found 391.9374.

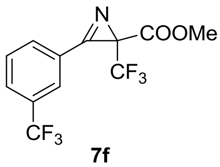

7f

Methyl 2-(trifluoromethyl)-3-(3-(trifluoromethyl)phenyl)-2*H*-azirine-2-carboxylate (7f)

Following the general procedure, **7f** was purified by silica gel chromatography (EtOAc/PE = 10/90). Yield: 168 mg, 54%, yellow oil. ^{1}H NMR (600 MHz, $CDCl_3$) δ 8.17 (s, 1H), 8.11 (d, *J* = 7.7 Hz, 1H), 8.00 (d, *J* = 7.9 Hz, 1H), 7.82 (t, *J* = 7.8 Hz, 1H), 3.83 (s, 3H). ^{19}F NMR (564 MHz, $CDCl_3$) δ -63.17, -65.80. ^{13}C NMR (150 MHz, $CDCl_3$) δ 166.2, 155.6, 134.1, 132.7 (q, *J* = 33.8 Hz), 131.7 (q, *J* = 5.1 Hz), 130.7, 127.6 (q, *J* = 3.7 Hz), 123.1 (q, *J* = 272.8 Hz), 122.6 (q, *J* = 275.4 Hz), 120.8, 53.4, 38.6 (q, *J* = 39.2 Hz). HRMS (ESI) calcd for $C_{12}H_7F_6NNaO_2^+$ [M + Na$^+$] 334.0273, found 334.0277.

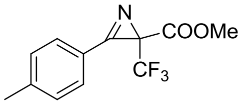

7g

Methyl 3-(*p*-tolyl)-2-(trifluoromethyl)-2*H*-azirine-2-carboxylate (7g)

Following the general procedure, **7g** was purified by silica gel chromatography (EtOAc/PE = 10/90). Yield: 103 mg, 40%, yellow oil. ^{1}H NMR (600 MHz, $CDCl_3$) δ 7.80 (d, *J* = 8.1 Hz, 2H), 7.43 (d, *J* = 7.9 Hz, 2H), 3.79 (s, 3H), 2.49 (s, 3H). ^{19}F NMR (564 MHz, $CDCl_3$) δ -65.71. ^{13}C NMR (151 MHz, $CDCl_3$) δ 166.8, 154.9, 146.6, 131.1, 130.5, 122.8 (q, *J* = 273.6 Hz), 116.7, 53.1, 37.8 (q, *J* = 38.9 Hz), 22.1. HRMS (ESI) calcd for $C_{12}H_{10}F_3NNaO_2^+$ [M + Na$^+$] 280.0556, found 280.0556.

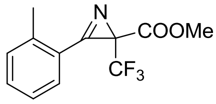

7h

Methyl 3-(*o*-tolyl)-2-(trifluoromethyl)-2*H*-azirine-2-carboxylate (7h)

Following the general procedure, **7h** was purified by silica gel chromatography

(EtOAc/PE = 10/90). Yield: 172 mg, 67%, yellow solid, m.p. 53-54 °C. ^{1}H NMR (600 MHz, $CDCl_3$) δ 7.72 – 7.56 (m, 2H), 7.51 – 7.36 (m, 2H), 3.80 (s, 3H), 2.75 (s, 3H). ^{19}F NMR (564 MHz, $CDCl_3$) δ -65.66. ^{13}C NMR (150 MHz, CDCl3) δ 166.9, 154.4, 142.8, 134.8, 132.9, 131.4, 126.8, 122.9 (q, J = 273.5 Hz), 118.5, 53.2, 36.4 (q, J = 38.2 Hz) , 20.1. HRMS (ESI) calcd for $C_{12}H_{10}F_3NNaO_2^+$ [M + Na$^+$] 280.0556, found 280.0558.

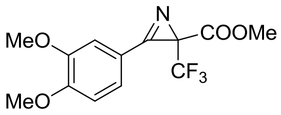

7i

Methyl 3-(3,4-dimethoxyphenyl)-2-(trifluoromethyl)-2*H*-azirine-2-carboxylate (7i)

Following the general procedure, **7i** was purified by silica gel chromatography (EtOAc/PE = 10/90). Yield: 154 mg, 51%, yellow oil. ^{1}H NMR (600 MHz, $CDCl_3$) δ 7.44 (td, J = 4.3, 1.8 Hz, 2H), 7.12 – 6.95 (m, 1H), 4.00 (s, 3H), 3.98 (s, 3H), 3.80 (s, 3H). ^{19}F NMR (564 MHz, $CDCl_3$) δ -65.75. ^{13}C NMR (150 MHz, $CDCl_3$) δ 166.9, 154.9, 154.2, 150.0, 126.5, 122.9 (q, J = 273.7 Hz), 111.9, 111.6, 111.4, 56.3, 56.2, 53.1, 38.1 (q, J = 38.2 Hz). HRMS (ESI) calcd for $C_{13}H_{12}F_3NNaO_4^+$ [M + Na$^+$] 326.0611, found 326.0605.

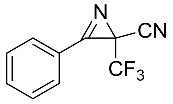

7j

3-Phenyl-2-(trifluoromethyl)-2*H*-azirine-2-carbonitrile (7j)

Following the general procedure, **7j** was purified by silica gel chromatography (EtOAc/PE = 10/90). Yield: 103 mg, 49%, yellow oil. ^{1}H NMR (600 MHz, $CDCl_3$) δ 7.97 (d, J = 7.5 Hz, 2H), 7.83 (t, J = 7.5 Hz, 1H), 7.71 (t, J = 7.5 Hz, 2H). ^{19}F NMR (564 MHz, $CDCl_3$) δ -67.89. ^{13}C NMR (150 MHz, $CDCl_3$) δ 156.3, 136.4, 131.5, 130.2, 121.1 (q, J = 274.4 Hz), 118.0, 114.2, 27.2 (q, J = 46.1 Hz). HRMS (ESI) calcd for $C_{10}H_5F_3N_2Na^+$ [M + Na$^+$] 233.0297, found 233.0296.

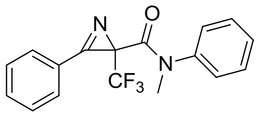

7k

N-Methyl-N,3-diphenyl-2-(trifluoromethyl)-2H-azirine-2-carboxamide (7k)

Following the general procedure, **7k** was purified by silica gel chromatography (EtOAc/PE = 10/90). Yield: 181 mg, 57%, yellow solid, m.p. 55-56 °C. ^{1}H NMR (600 MHz, $CDCl_3$) δ 7.76 (s, 2H), 7.62 (t, J = 7.3 Hz, 1H), 7.50 (t, J = 7.2 Hz, 2H), 7.31 (t, J = 7.2 Hz, 2H), 7.24 (t, J = 7.3 Hz, 1H), 7.17 (d, J = 7.4 Hz, 2H), 3.31 (s, 3H). ^{19}F NMR (564 MHz, $CDCl_3$) δ -65.77. ^{13}C NMR (150 MHz, $CDCl_3$) δ 164.8, 141.7, 134.4, 130.80, 129.5, 129.2, 128.1, 127.5, 123.3 (q, J = 275.6 Hz), 120.6, 40.0 (q, J = 38.1 Hz), 39.04. HRMS (ESI) calcd for $C_{17}H_{13}F_3N_2NaO^+$ [M + Na^+] 341.0872, found 341.0876.

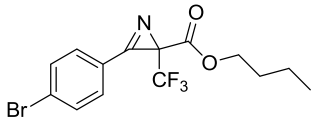

7l

n-Butyl 3-(4-bromophenyl)-2-(trifluoromethyl)-2H-azirine-2-carboxylate (7l)

Following the general procedure, **7l** was purified by silica gel chromatography (EtOAc/PE = 10/90). Yield: 225 mg, 62%, yellow oil. ^{1}H NMR (400 MHz, DMSO) δ 8.16 – 7.91 (m, 4H), 4.19 (td, J = 6.4, 2.3 Hz,2H), 1.54 (dt, J = 14.4, 6.5 Hz, 2H), 1.33 – 1.10 (m, 2H), 0.83 (t, J = 7.4 Hz, 3H). ^{19}F NMR (375 MHz, DMSO) δ -64.78. ^{13}C NMR (100MHz, DMSO) δ 165.6, 155.3, 134.0, 133.3, 130.9, 123.2 (q, J = 273.6 Hz) 118.0, 66.5, 37.8 (q, J = 37.6 Hz) 30.2, 18.8, 13.8. HRMS (ESI) calcd for $C_{14}H_{13}{}^{79}BrF_3NNaO_2{}^+$ [M + Na^+] 385.9974, found 385.9984.

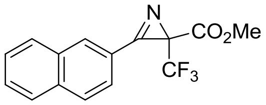

7m

Methyl 3-(naphthalen-2-yl)-2-(trifluoromethyl)-2H-azirine-2-carboxylate (7m)

Following the general procedure, **7m** was purified by silica gel chromatography

(EtOAc/PE = 10/90). Yield: 126 mg, 43%, yellow solid, m.p. 90-91 °C. ^{1}H NMR (600 MHz, $CDCl_3$) δ 8.33 (s, 1H), 8.07 (d, *J* = 8.5 Hz, 1H), 8.00 (t, *J* = 8.5 Hz, 2H), 7.96 (d, *J* = 8.2 Hz, 1H), 7.71 (t, *J* = 7.6 Hz, 1H), 7.68 – 7.59 (m, 1H), 3.81 (s, 3H). ^{19}F NMR (564 MHz, $CDCl_3$) δ -65.59. ^{13}C NMR (150 MHz, $CDCl_3$) δ 166.9, 155.7, 136.5, 134.1, 132.8, 130.1, 130.0, 129.6, 128.4, 128.0, 125.0, 123.0 (q, *J* = 273.5 Hz), 116.9, 53.3, 38.3 (q, *J* = 38.5 Hz). HRMS (ESI) calcd for $C_{15}H_{10}F_3NNaO_2^+$ [M + Na^+] 316.0556, found 316.0550.

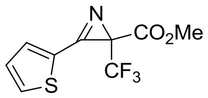

7n

Methyl 3-(thiophen-2-yl)-2-(trifluoromethyl)-2*H*-azirine-2-carboxylate (7n)

Following the general procedure, **7n** was purified by silica gel chromatography (EtOAc/PE = 10/90). Yield: 112 mg, 45%, yellow solid, m.p. 86-87 °C. ^{1}H NMR (600 MHz, $CDCl_3$) δ 8.01 (d, *J* = 5.0 Hz, 1H), 7.77 (d, *J* = 3.8 Hz, 1H), 7.35 – 7.30 (m, 1H), 3.81 (s, 3H). ^{19}F NMR (564 MHz, $CDCl_3$) δ -66.05. ^{13}C NMR (150 MHz, $CDCl_3$) δ 166.5, 148.9, 137.8, 137.4, 129.2, 122.7 (q, *J* = 274.0 Hz), 121.3, 53.3, 38.7 (q, *J* = 38.6 Hz). HRMS (ESI) calcd for $C_9H_6F_3NNaO_2S^+$ [M + Na^+] 271.9964, found 271.9956.

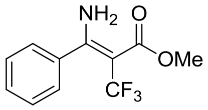

6a

(*E*)-Methyl 3-amino-3-phenyl-2-(trifluoromethyl)acrylate (6a)

Following the above procedure, **6a** was purified by silica gel chromatography (EtOAc/PE = 10/90). Yield: 56 mg, 23%, yellow oil. ^{1}H NMR (600 MHz, $CDCl_3$) δ 9.31 (brs, 1H), 7.47 – 7.40 (m, 3H), 7.37 (d, *J* = 7.5 Hz, 2H), 5.02 (brs, 1H), 3.83 (s, 3H). ^{19}F NMR (564 MHz, $CDCl_3$) δ -50.4. ^{13}C NMR (150 MHz, $CDCl_3$) δ 168.4, 164.7, 137.3, 130.0, 128.4, 126.9 (d, *J* = 1.5 Hz), 125.1 (q, *J* = 267.6 Hz), 89.0 (q, *J* = 32.3 Hz), 51.5. HRMS (ESI) calcd for $C_{10}H_{11}F_3NNaO_2^+$ [M + Na^+] 268.0556, found 268.0562.

^{1}H NMR, ^{19}F NMR and ^{13}C NMR spectra of **7** and intermediate **6a**

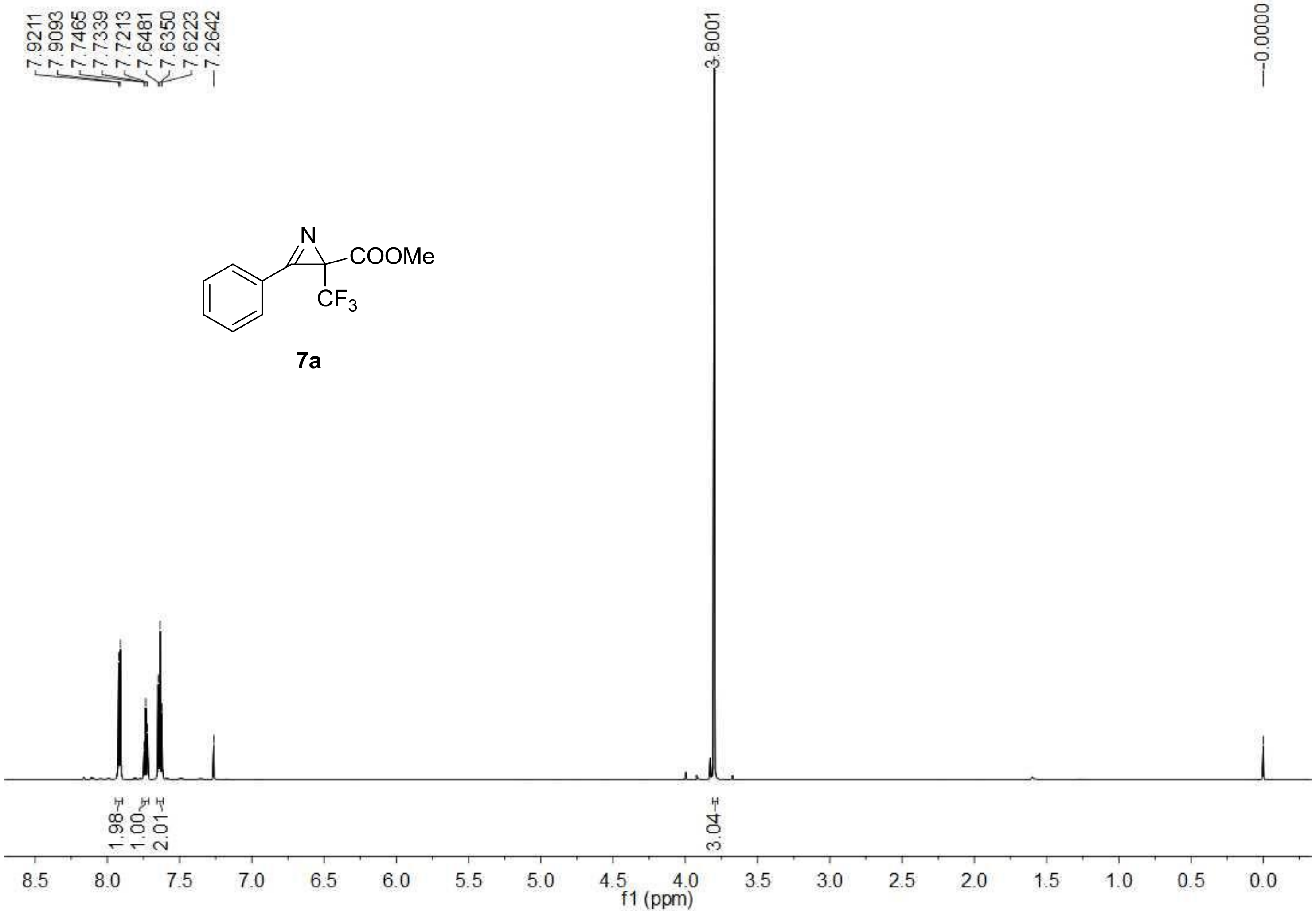

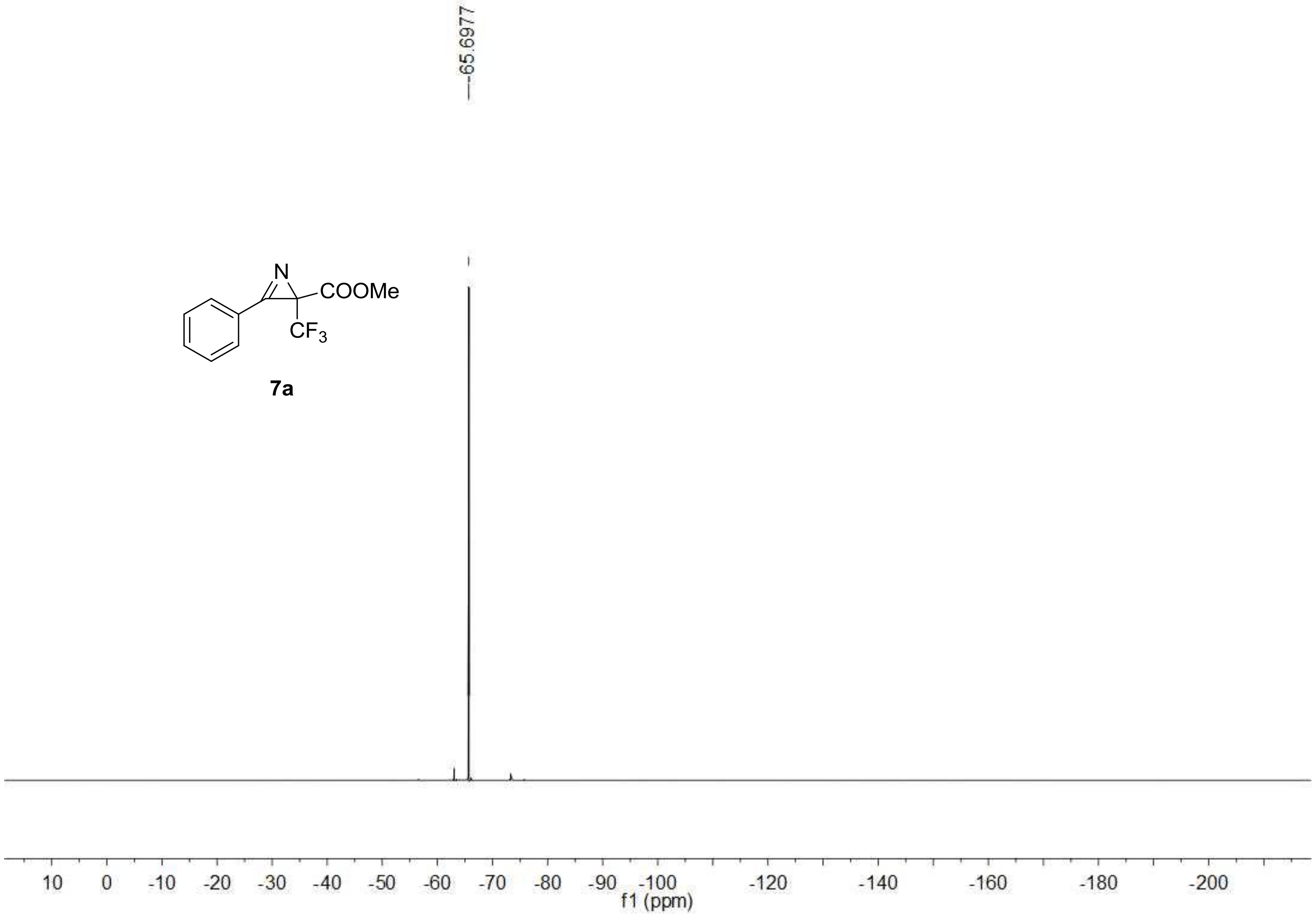
-65.6977
N
COOMe
CF3
7a
10
0
-10
-20
-30
-40
-50
-60
-70
-80
-90
-100
-120
-140
-160
-180
-200
f1 (ppm)

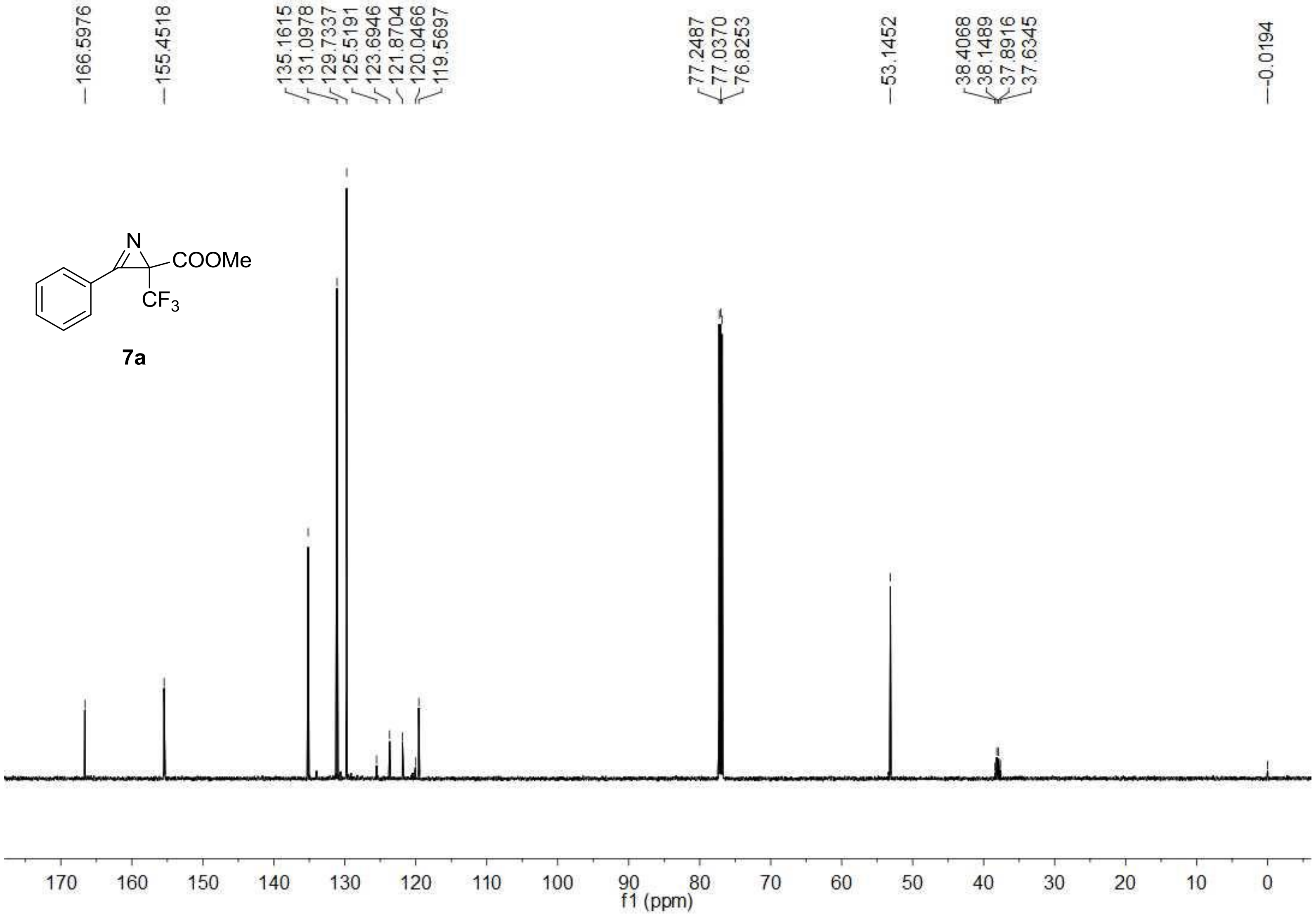

166.5976
155.4518
135.1615
131.0978
129.7337
125.5191
123.6946
121.8704
120.0466
119.5697
77.2487
77.0370
76.8253
53.1452
38.4068
38.1489
37.8916
37.6345
-0.0194
N
COOMe
CF3
7a
170
160
150
140
130
120
110
100
90
80
70
60
50
40
30
20
10
0
f1 (ppm)

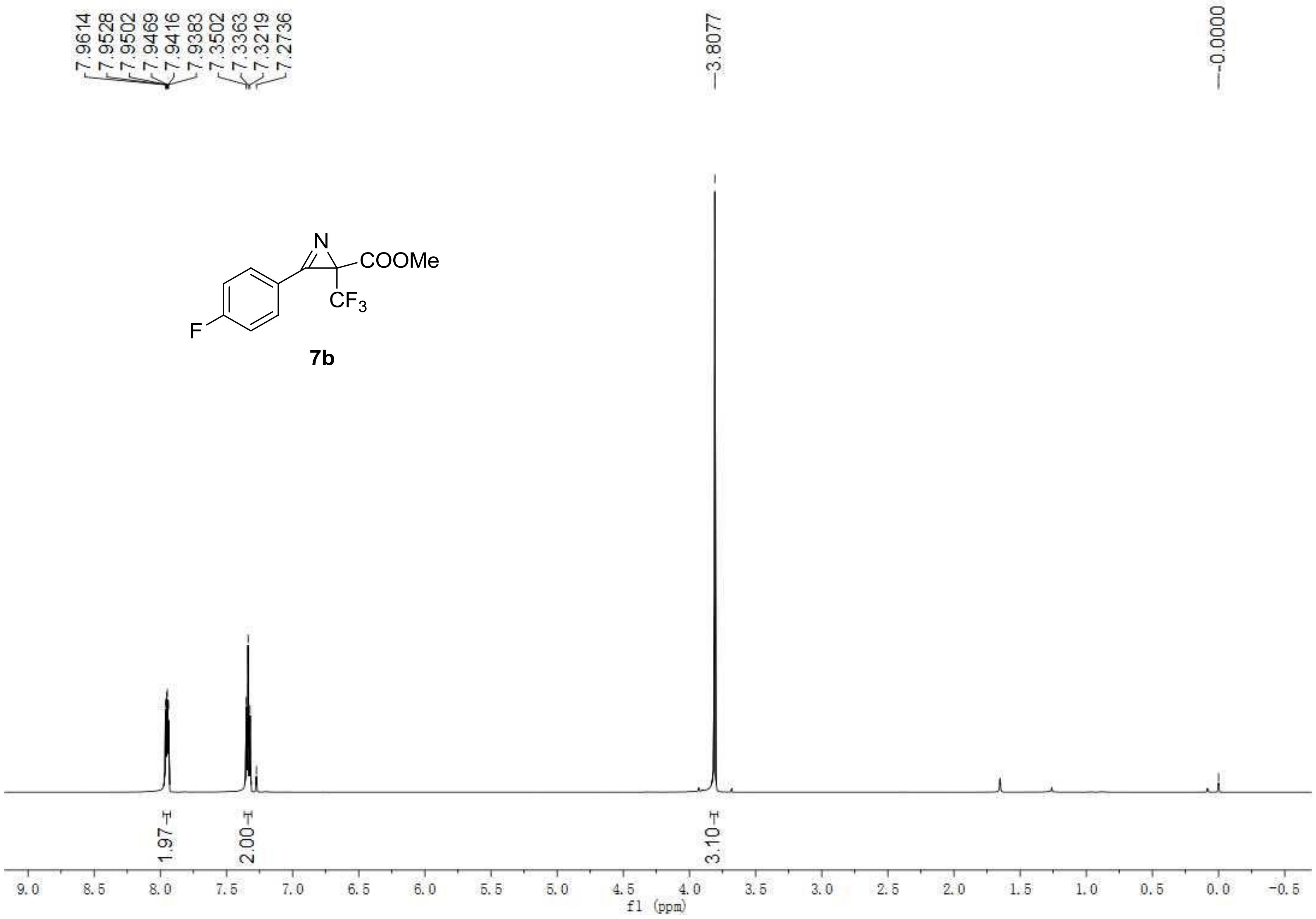
7.9614
7.9528
7.9502
7.9469
7.9416
7.9383
7.3502
7.3363
7.3219
7.2736
3.8077
0.0000
N
COOMe
CF3
F
7b
1.97
2.00
3.10
9.0 8.5 8.0 7.5 7.0 6.5 6.0 5.5 5.0 4.5 4.0 3.5 3.0 2.5 2.0 1.5 1.0 0.5 0.0 -0.5
f1 (ppm)

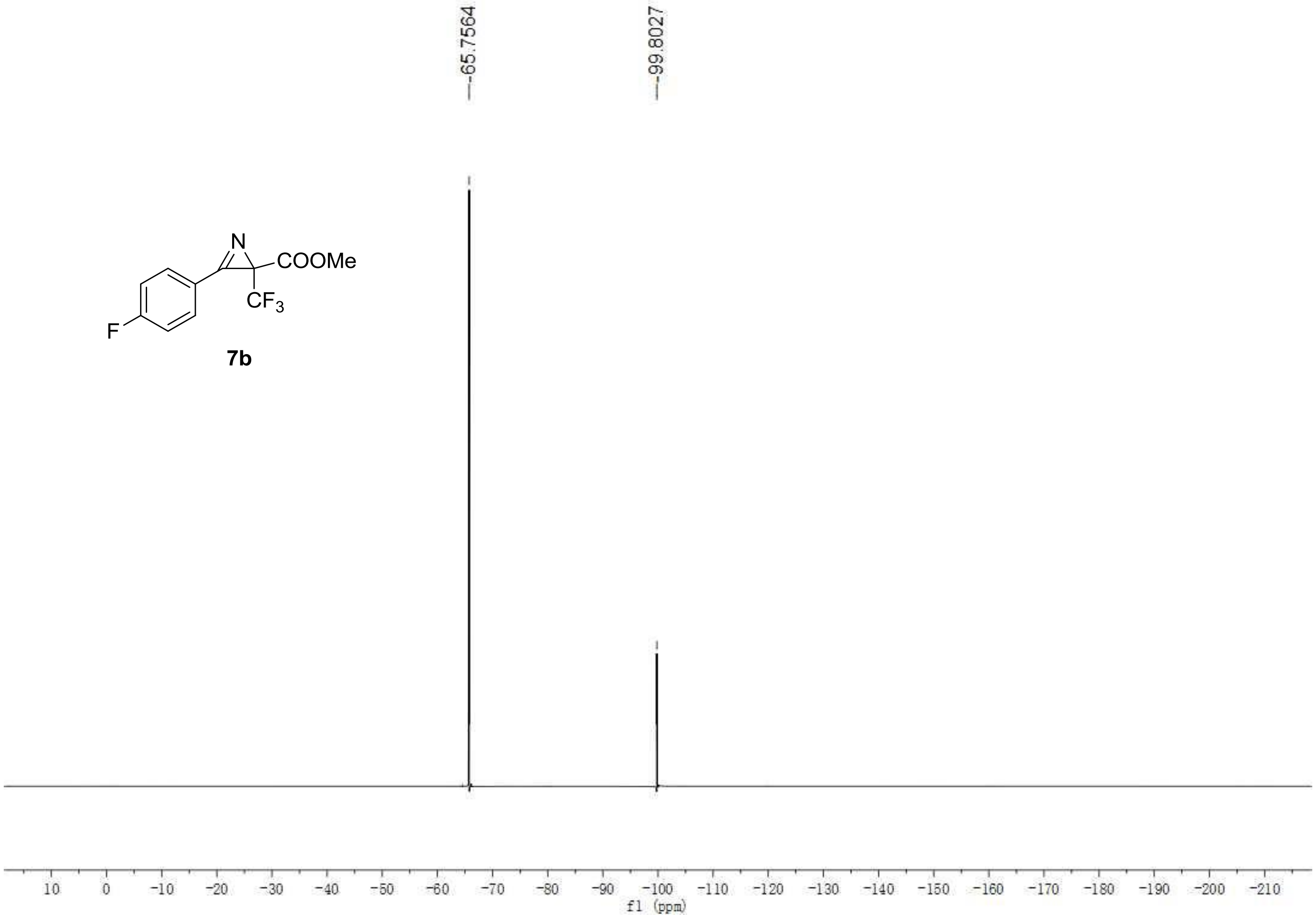
-65.7564
-99.8027
N
COOMe
CF3
F
7b
10 0 -10 -20 -30 -40 -50 -60 -70 -80 -90 -100 -110 -120 -130 -140 -150 -160 -170 -180 -190 -200 -210
f1 (ppm)

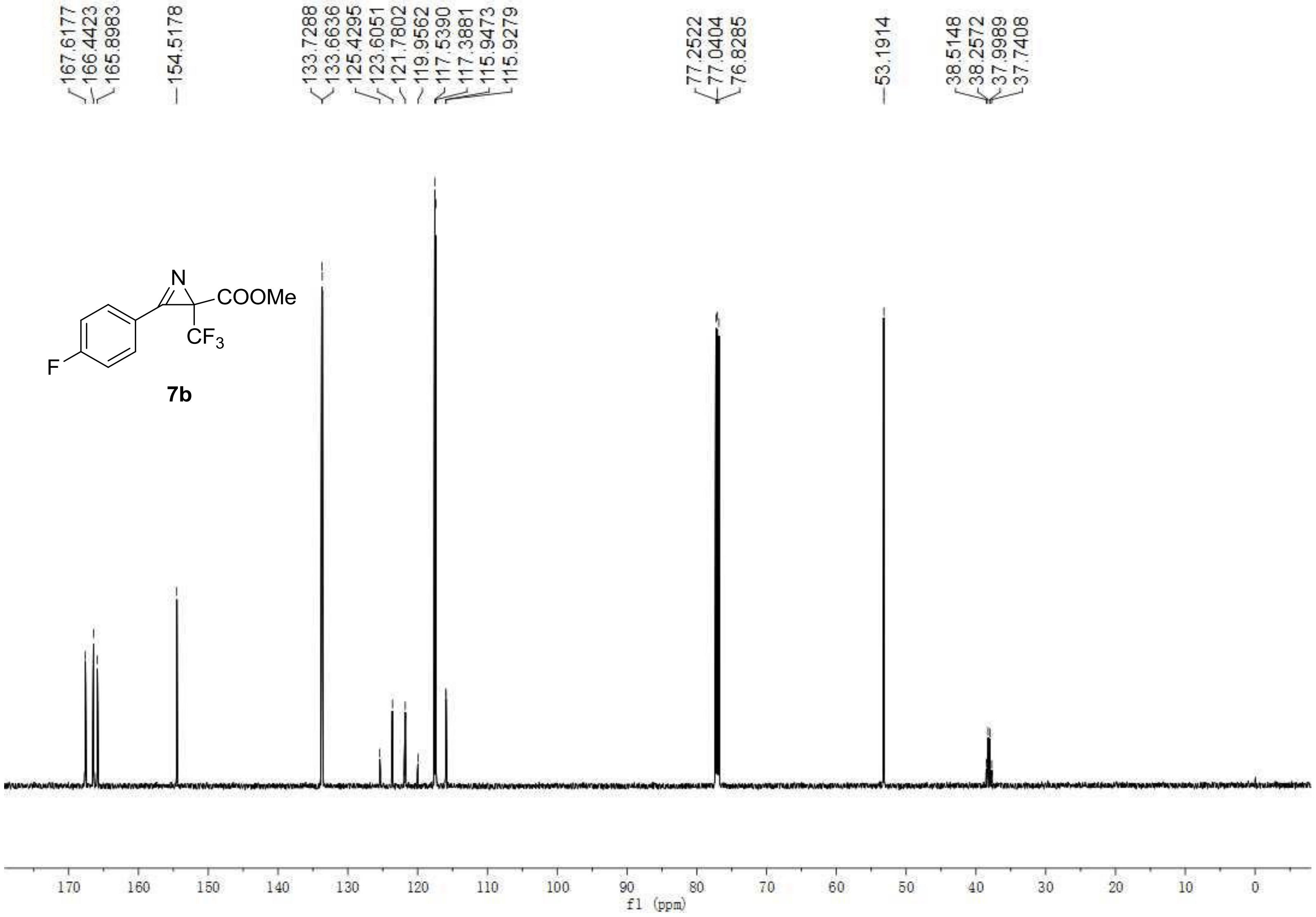
167.6177
166.4423
165.8983
154.5178
133.7288
133.6636
125.4295
123.6051
121.7802
119.9562
117.5390
117.3881
115.9473
115.9279
77.2522
77.0404
76.8285
53.1914
38.5148
38.2572
37.9989
37.7408
N
COOMe
CF3
F
7b
170
160
150
140
130
120
110
100
90
80
70
60
50
40
30
20
10
0
f1 (ppm)

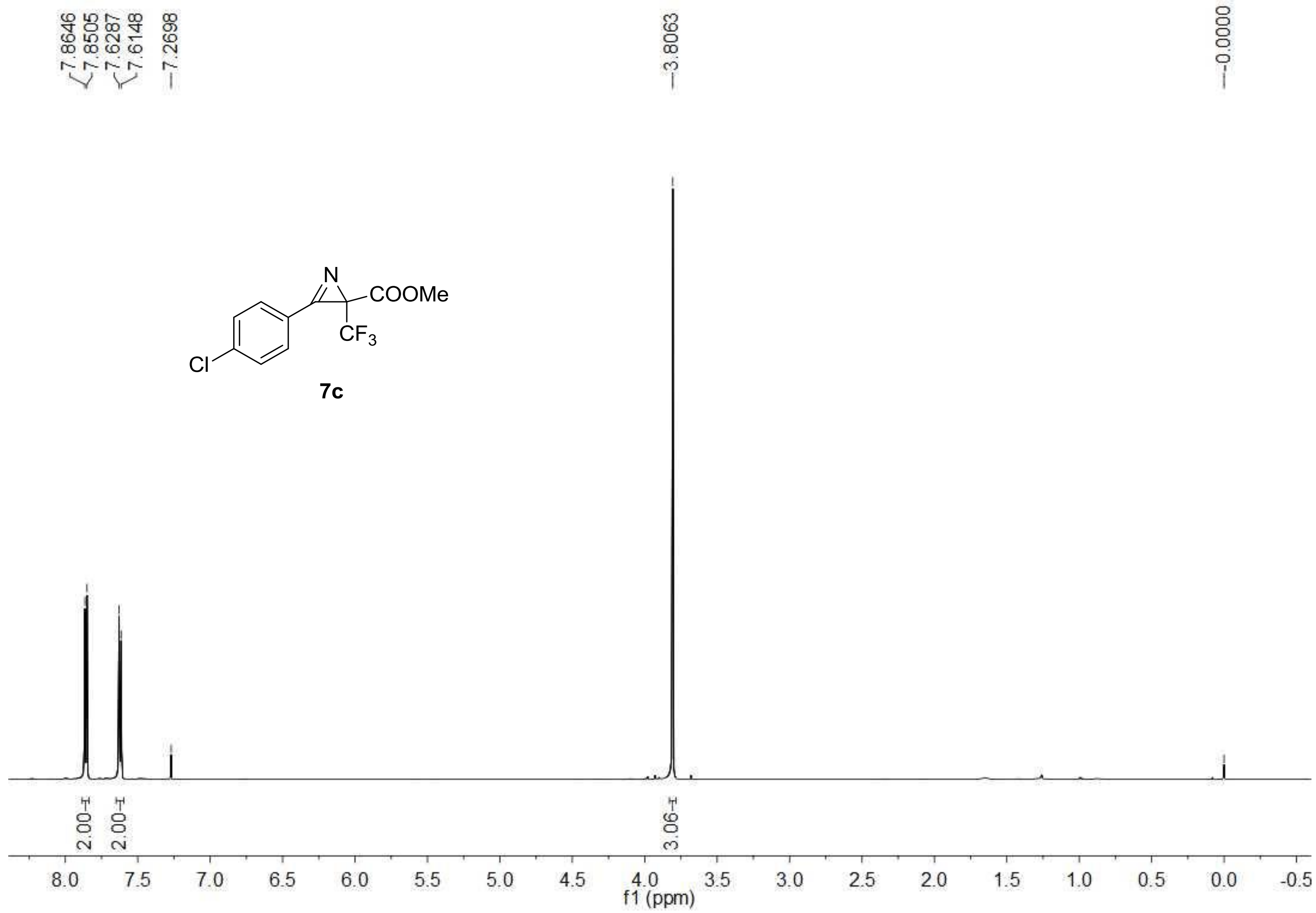
7.8646
7.8505
7.6287
7.6148
7.2698
3.8063
0.0000
N
COOMe
CF3
Cl
7c
2.00
2.00
3.06
8.0 7.5 7.0 6.5 6.0 5.5 5.0 4.5 4.0 3.5 3.0 2.5 2.0 1.5 1.0 0.5 0.0 -0.5
f1 (ppm)

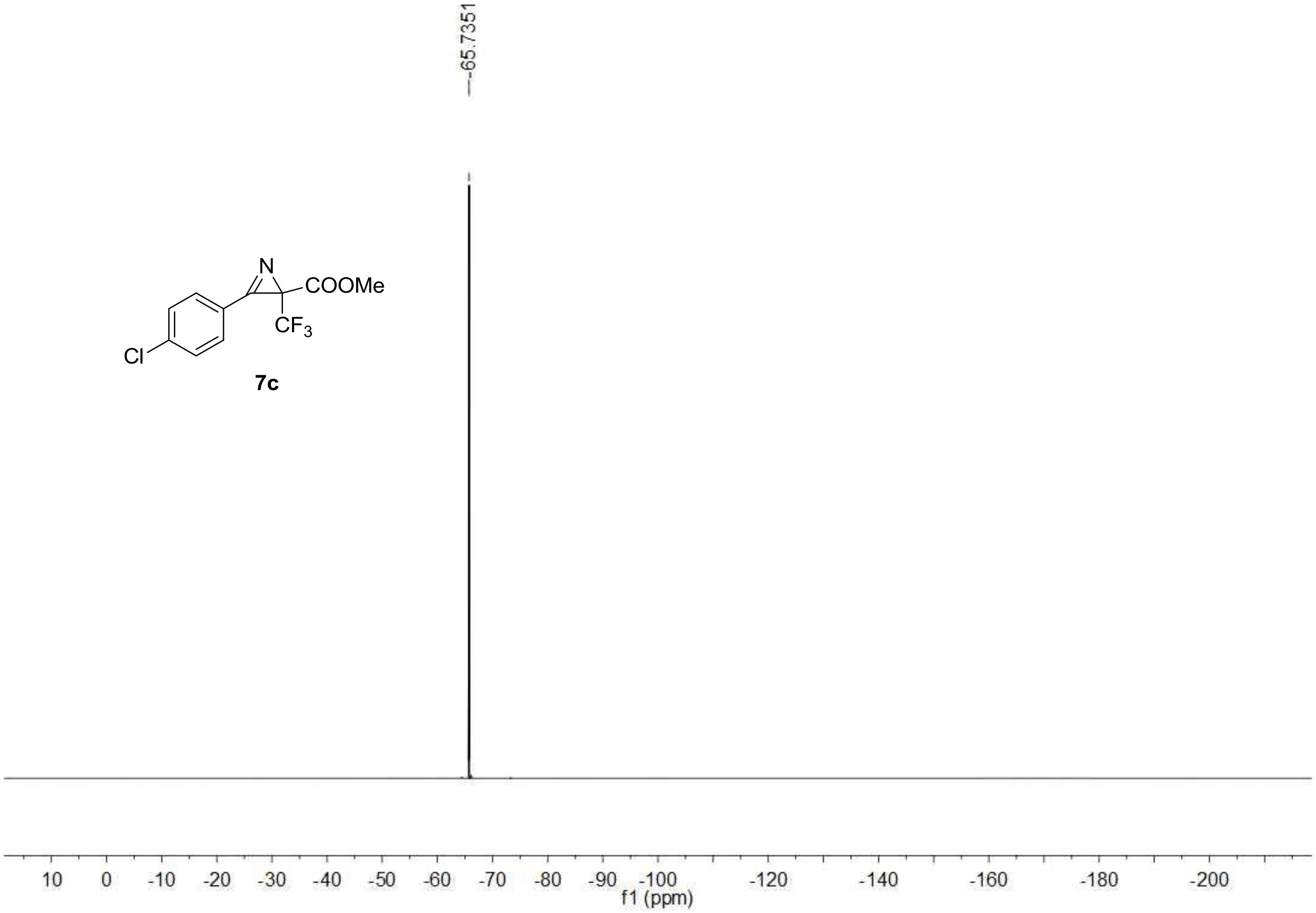
7c
-65.7351
10
0
-10
-20
-30
-40
-50
-60
-70
-80
-90
-100
-120
-140
-160
-180
-200
f1 (ppm)

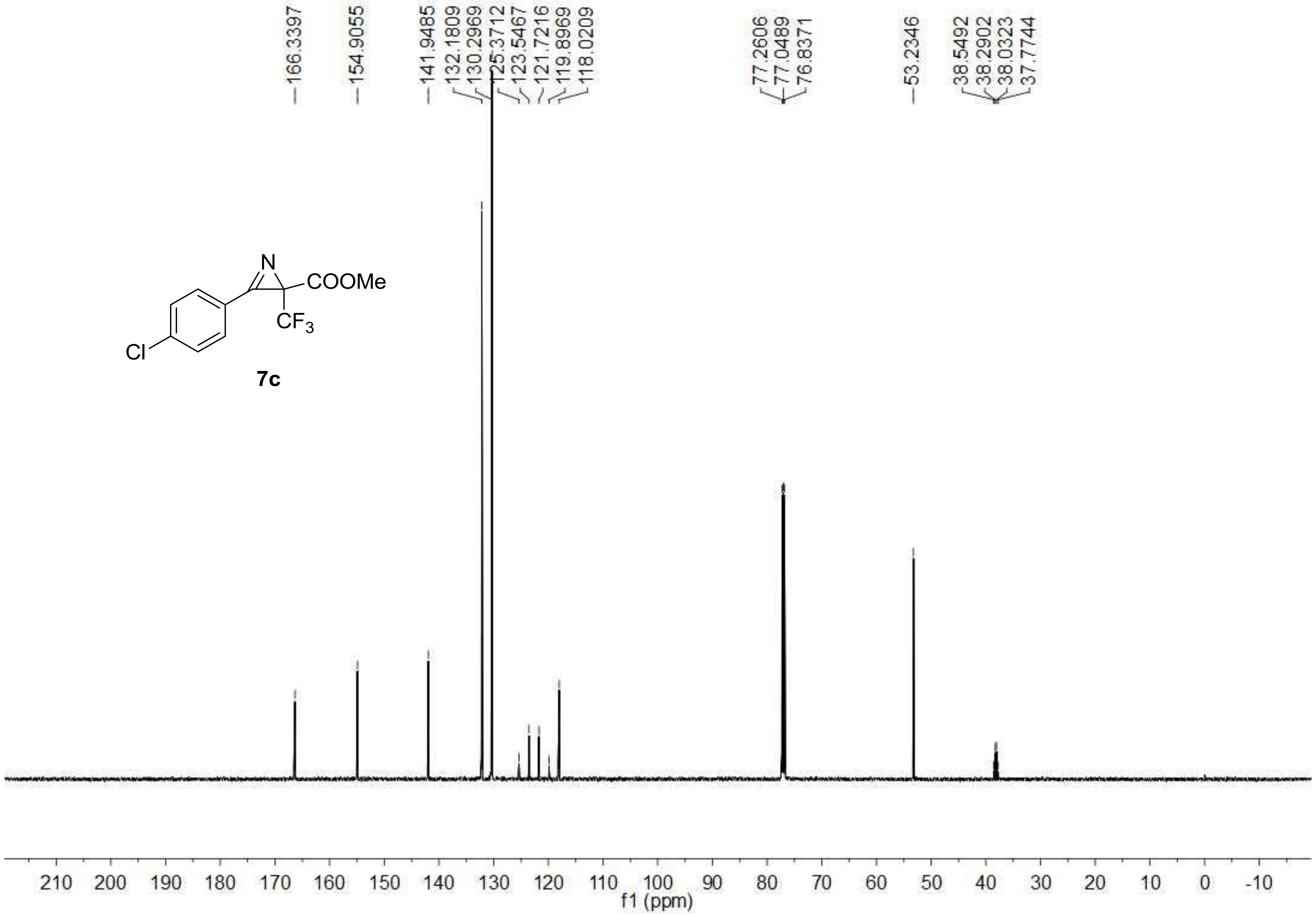
166.3397
154.9055
141.9485
132.1809
130.2969
125.3712
123.5467
121.7216
119.8969
118.0209
77.2606
77.0489
76.8371
53.2346
38.5492
38.2902
38.0323
37.7744
N
COOMe
CF3
Cl
7c
210 200 190 180 170 160 150 140 130 120 110 100 90 80 70 60 50 40 30 20 10 0 -10
f1 (ppm)

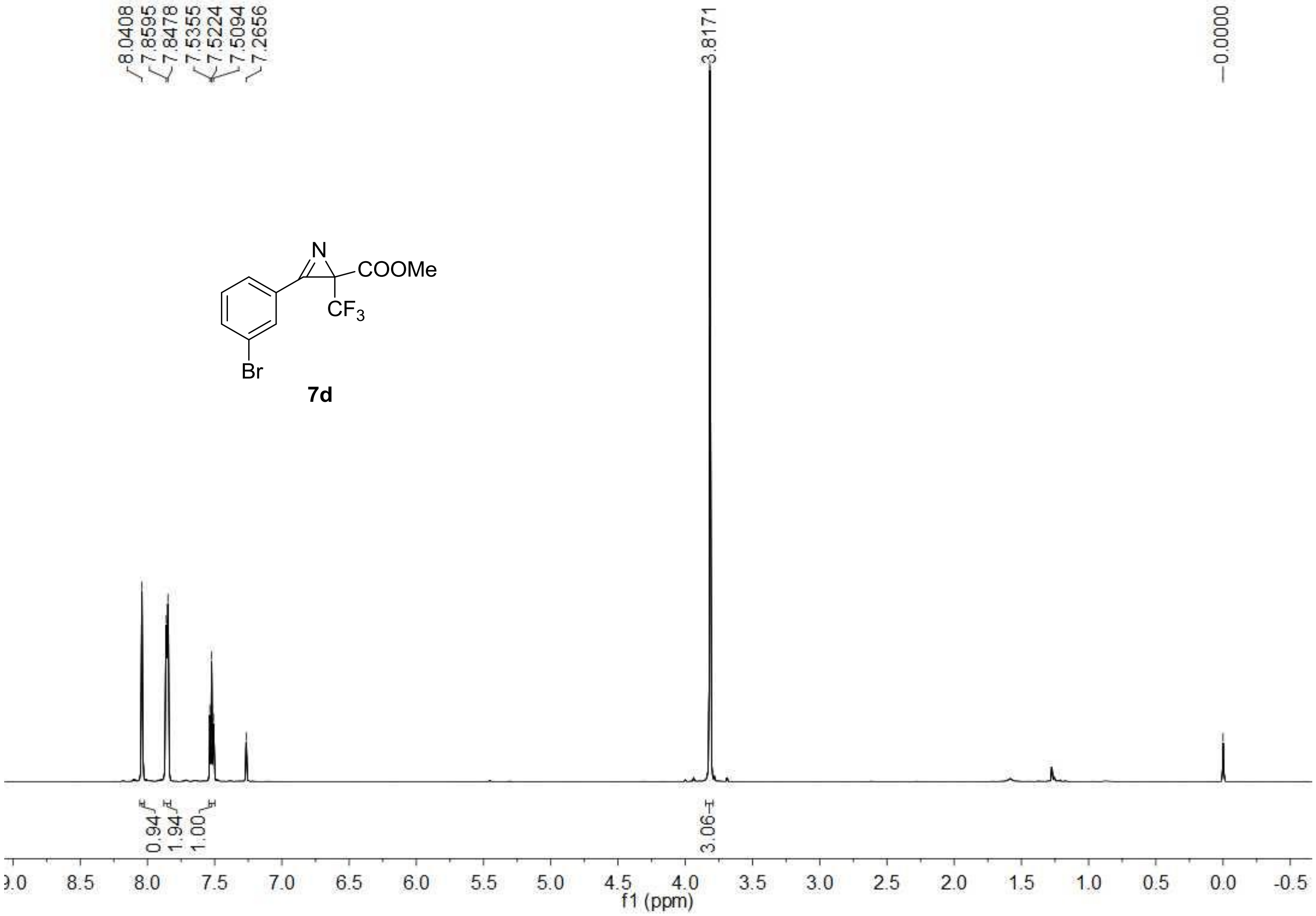
8.0408
7.8595
7.8478
7.5355
7.5224
7.5094
7.2656
3.8171
0.0000
N
COOMe
CF3
Br
7d
0.94
1.94
1.00
3.06
9.0 8.5 8.0 7.5 7.0 6.5 6.0 5.5 5.0 4.5 4.0 3.5 3.0 2.5 2.0 1.5 1.0 0.5 0.0 -0.5
f1 (ppm)

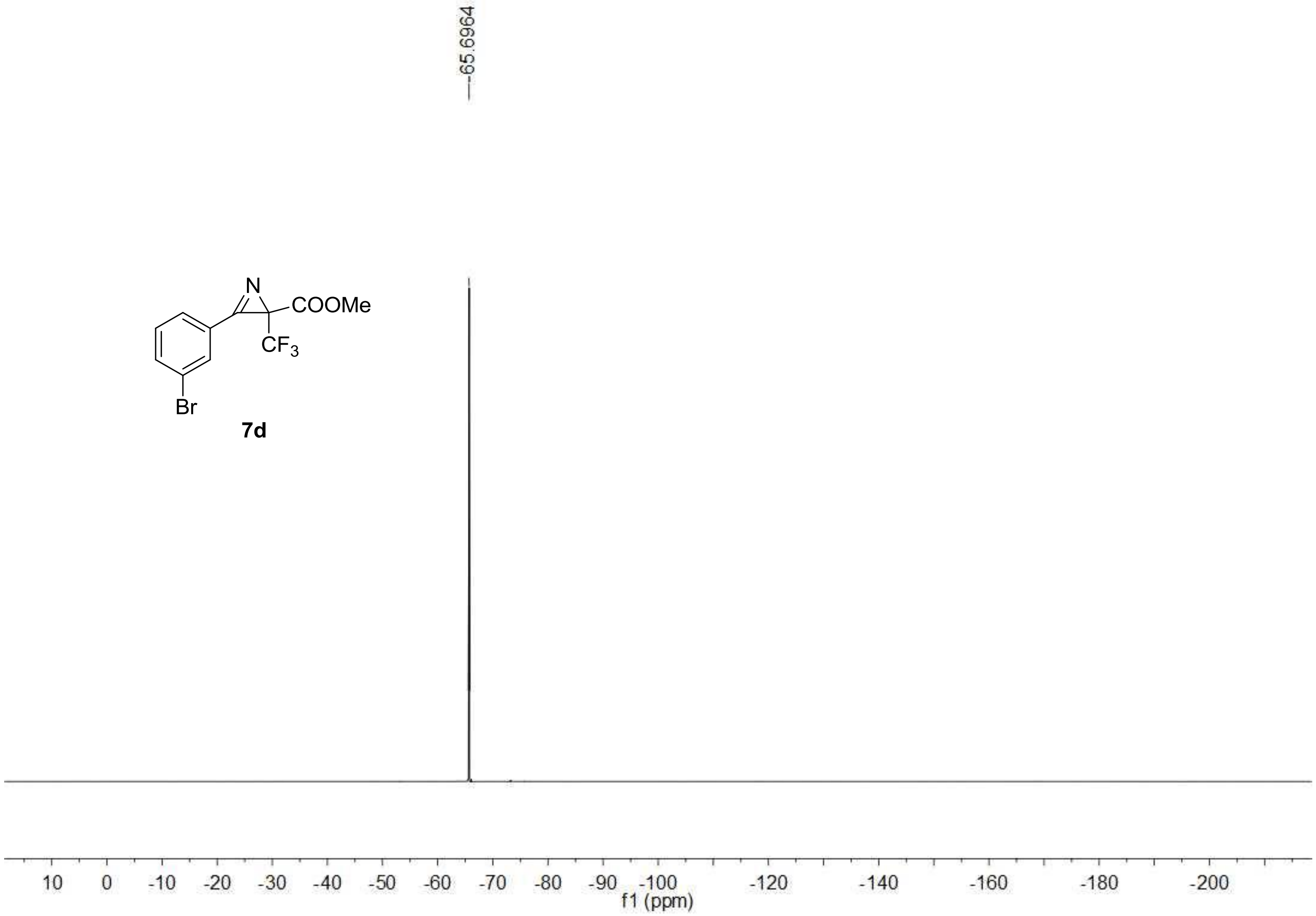
-65.6964
N
COOMe
CF3
Br
7d
10
0
-10
-20
-30
-40
-50
-60
-70
-80
-90
-100
-120
-140
-160
-180
-200
f1 (ppm)

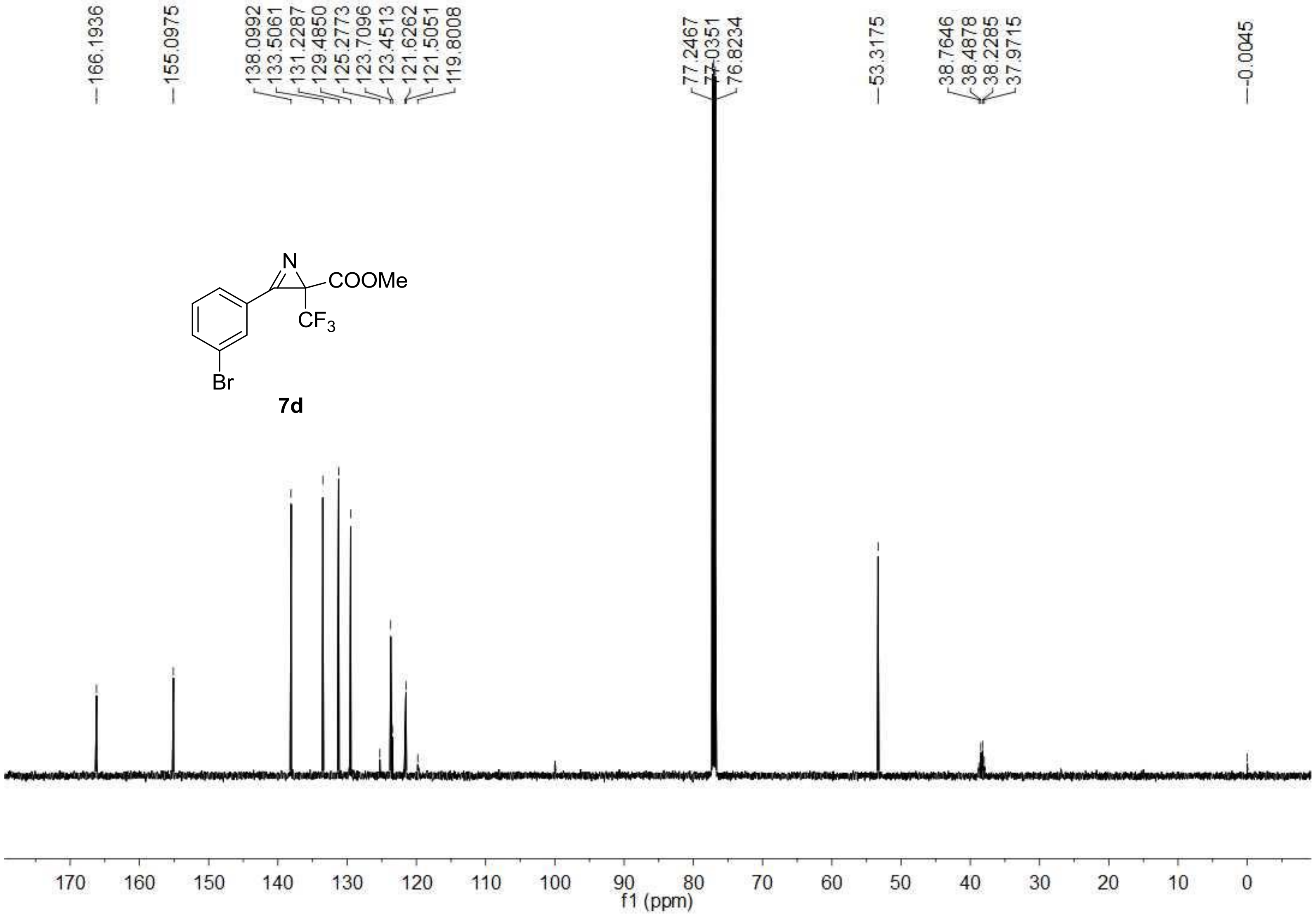
166.1936
155.0975
138.0992
133.5061
131.2287
129.4850
125.2773
123.7096
123.4513
121.6262
121.5051
119.8008
77.2467
77.0351
76.8234
53.3175
38.7646
38.4878
38.2285
37.9715
-0.0045
N
COOMe
CF3
Br
7d
170 160 150 140 130 120 110 100 90 80 70 60 50 40 30 20 10 0
f1 (ppm)

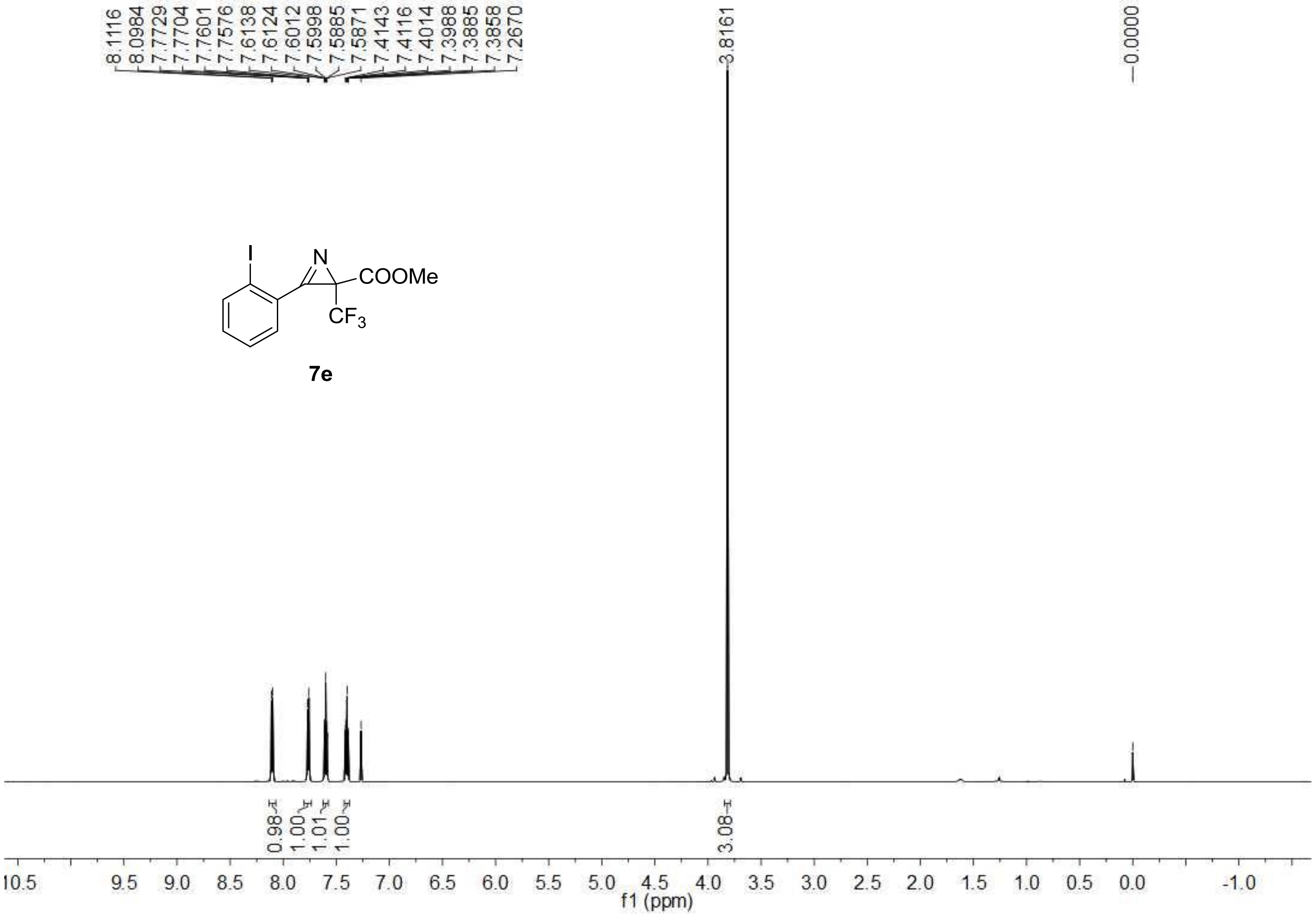
8.1116
8.0984
7.7729
7.7704
7.7601
7.7576
7.6138
7.6124
7.6012
7.5998
7.5885
7.5871
7.4143
7.4116
7.4014
7.3988
7.3885
7.3858
7.2670
3.8161
0.0000
I
N
COOMe
CF3
7e
0.98
1.00
1.01
1.00
3.08
10.5
9.5
9.0
8.5
8.0
7.5
7.0
6.5
6.0
5.5
5.0
4.5
4.0
3.5
3.0
2.5
2.0
1.5
1.0
0.5
0.0
-1.0
f1 (ppm)

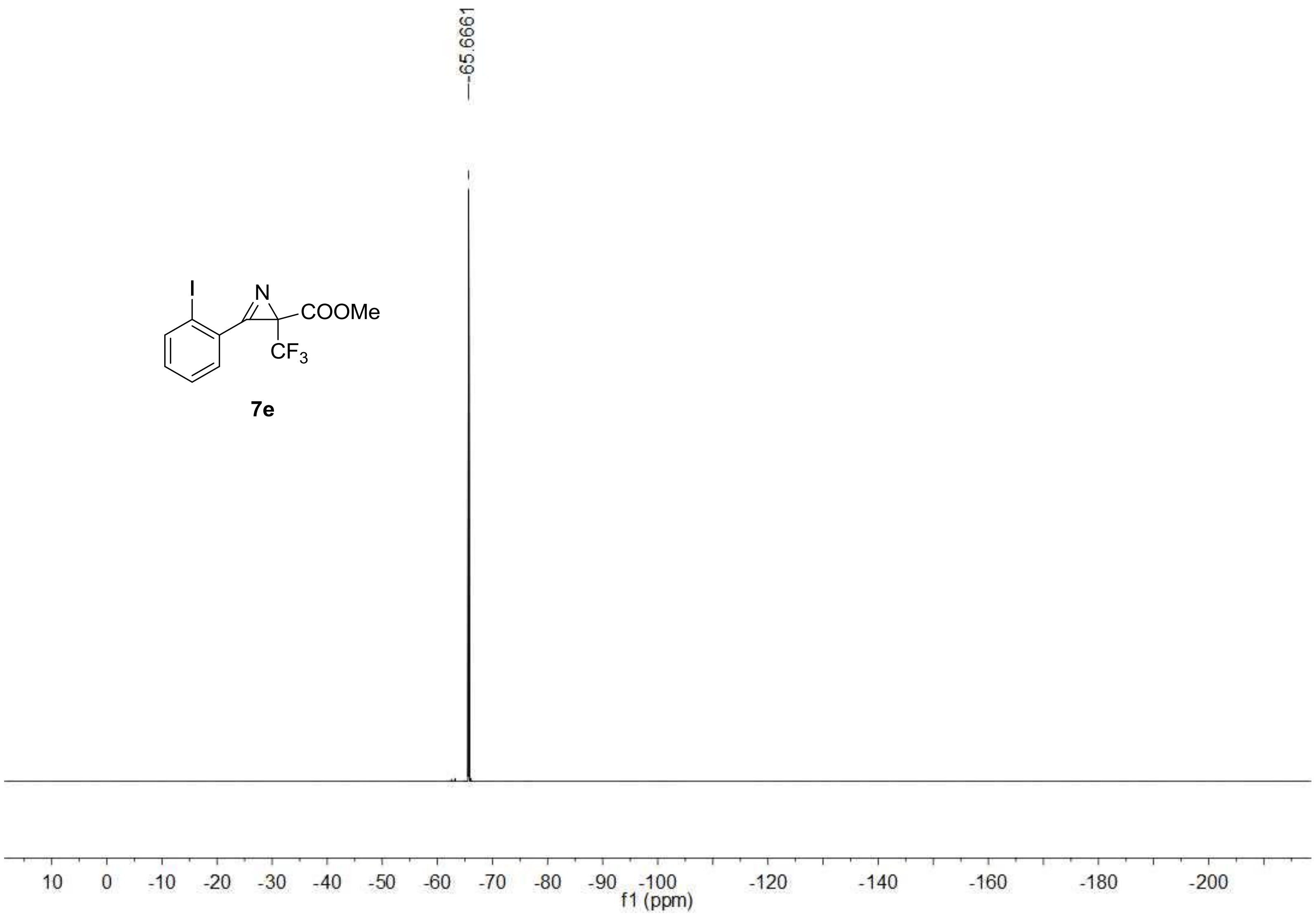
-65.6661
I
N
COOMe
CF_3
7e
10 0 -10 -20 -30 -40 -50 -60 -70 -80 -90 -100 -120 -140 -160 -180 -200
f1 (ppm)

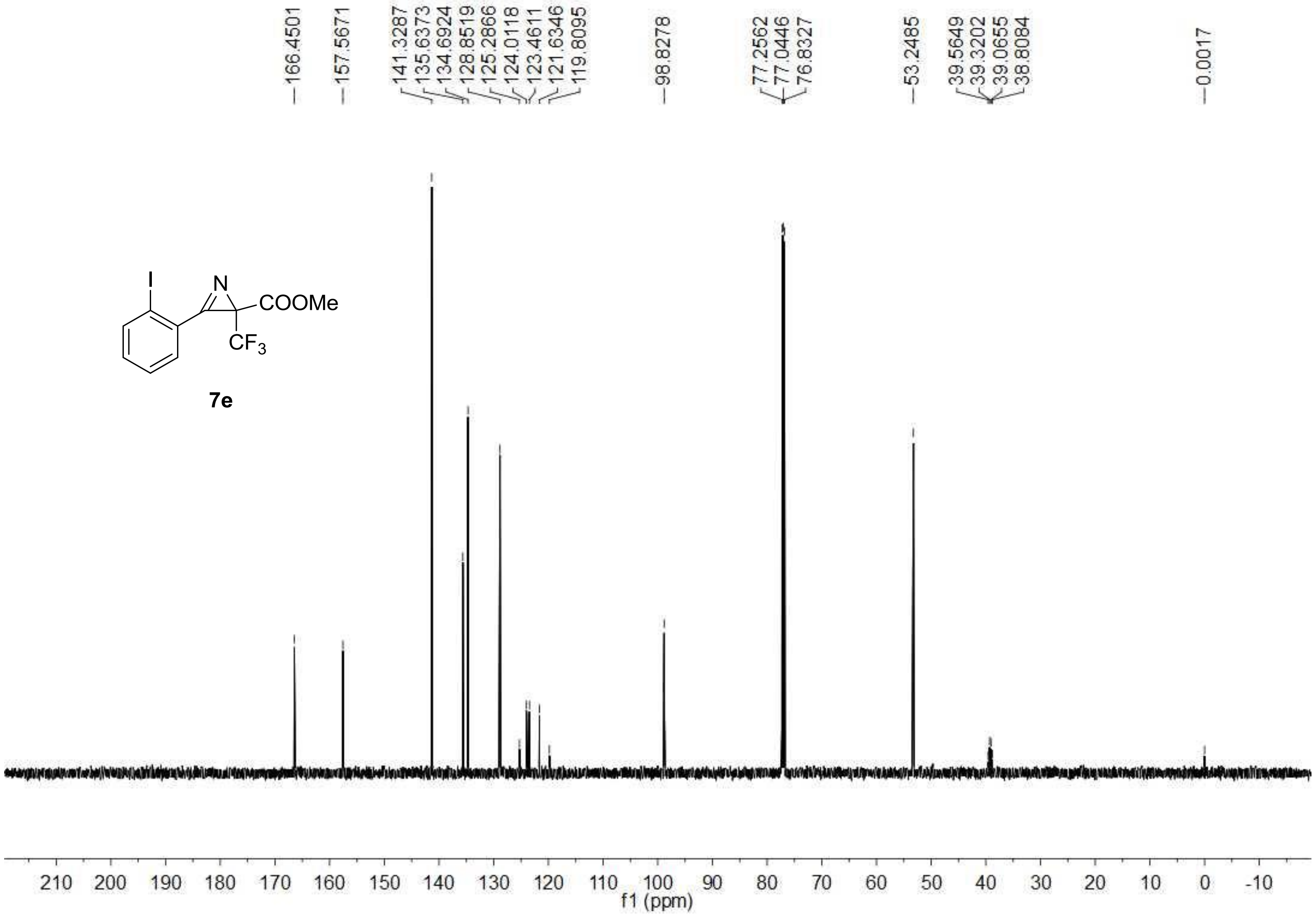
166.4501
157.5671
141.3287
135.6373
134.6924
128.8519
125.2866
124.0118
123.4611
121.6346
119.8095
98.8278
77.2562
77.0446
76.8327
53.2485
39.5649
39.3202
39.0655
38.8084
0.0017
I
N
COOMe
CF_3
7e
210 200 190 180 170 160 150 140 130 120 110 100 90 80 70 60 50 40 30 20 10 0 -10
f1 (ppm)

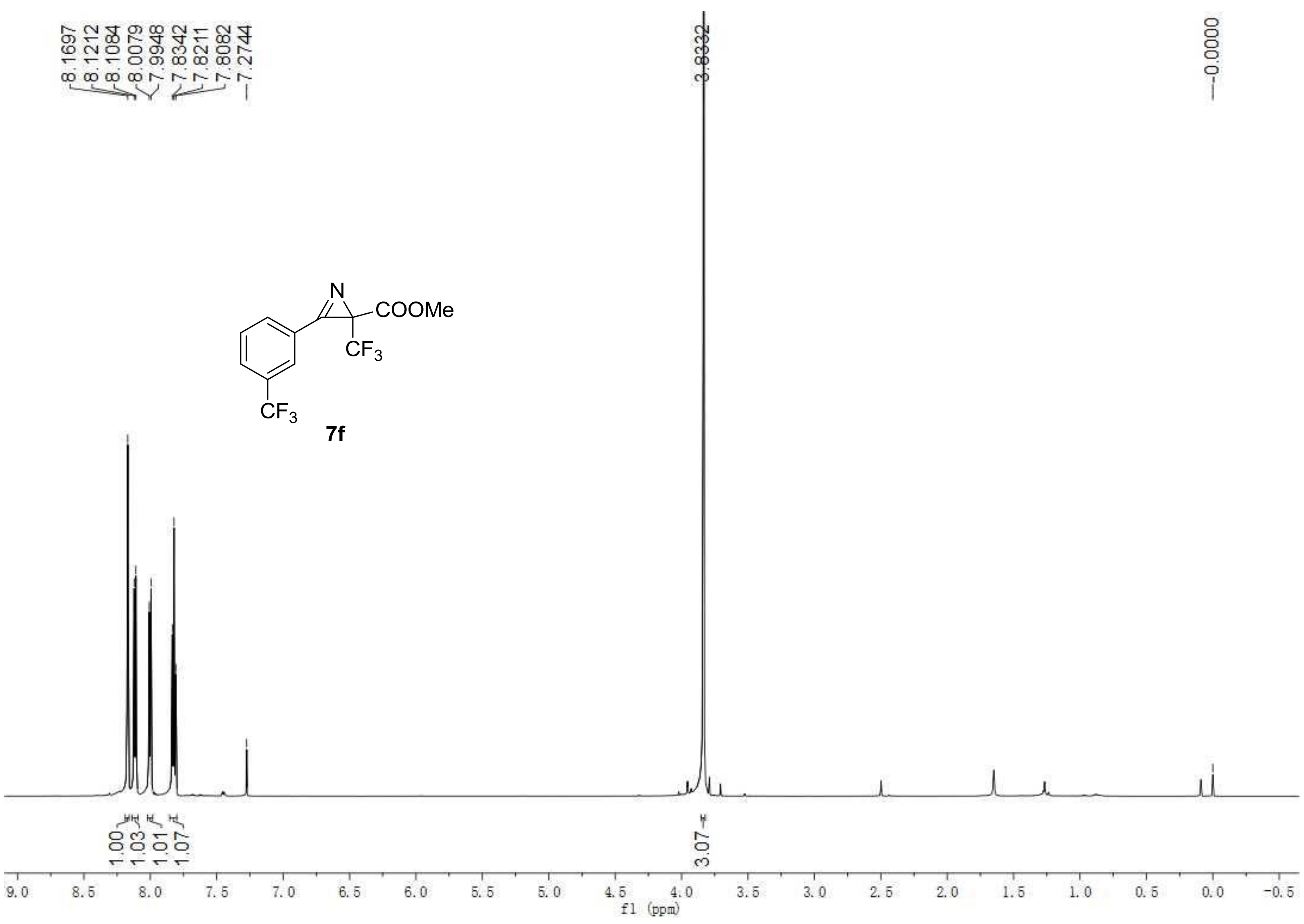
8.1697
8.1212
8.1084
8.0079
7.9948
7.8342
7.8211
7.8082
7.2744
3.8332
0.0000
N
COOMe
CF3
CF3
7f
1.00
1.03
1.01
1.07
3.07
9.0
8.5
8.0
7.5
7.0
6.5
6.0
5.5
5.0
4.5
4.0
3.5
3.0
2.5
2.0
1.5
1.0
0.5
0.0
-0.5
f1 (ppm)

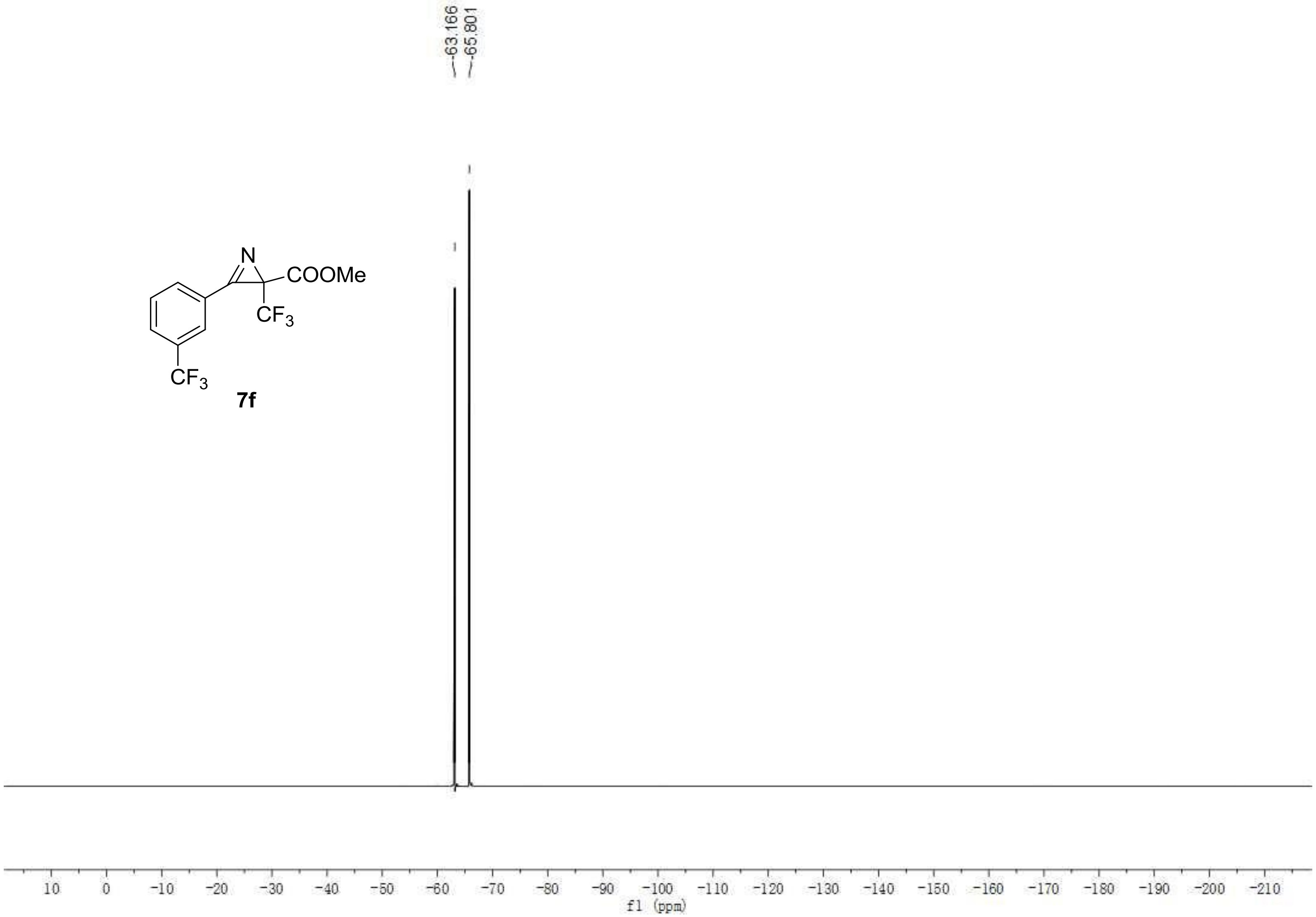
-63.166
-65.801
N
COOMe
CF3
CF3
7f
10 0 -10 -20 -30 -40 -50 -60 -70 -80 -90 -100 -110 -120 -130 -140 -150 -160 -170 -180 -190 -200 -210
f1 (ppm)

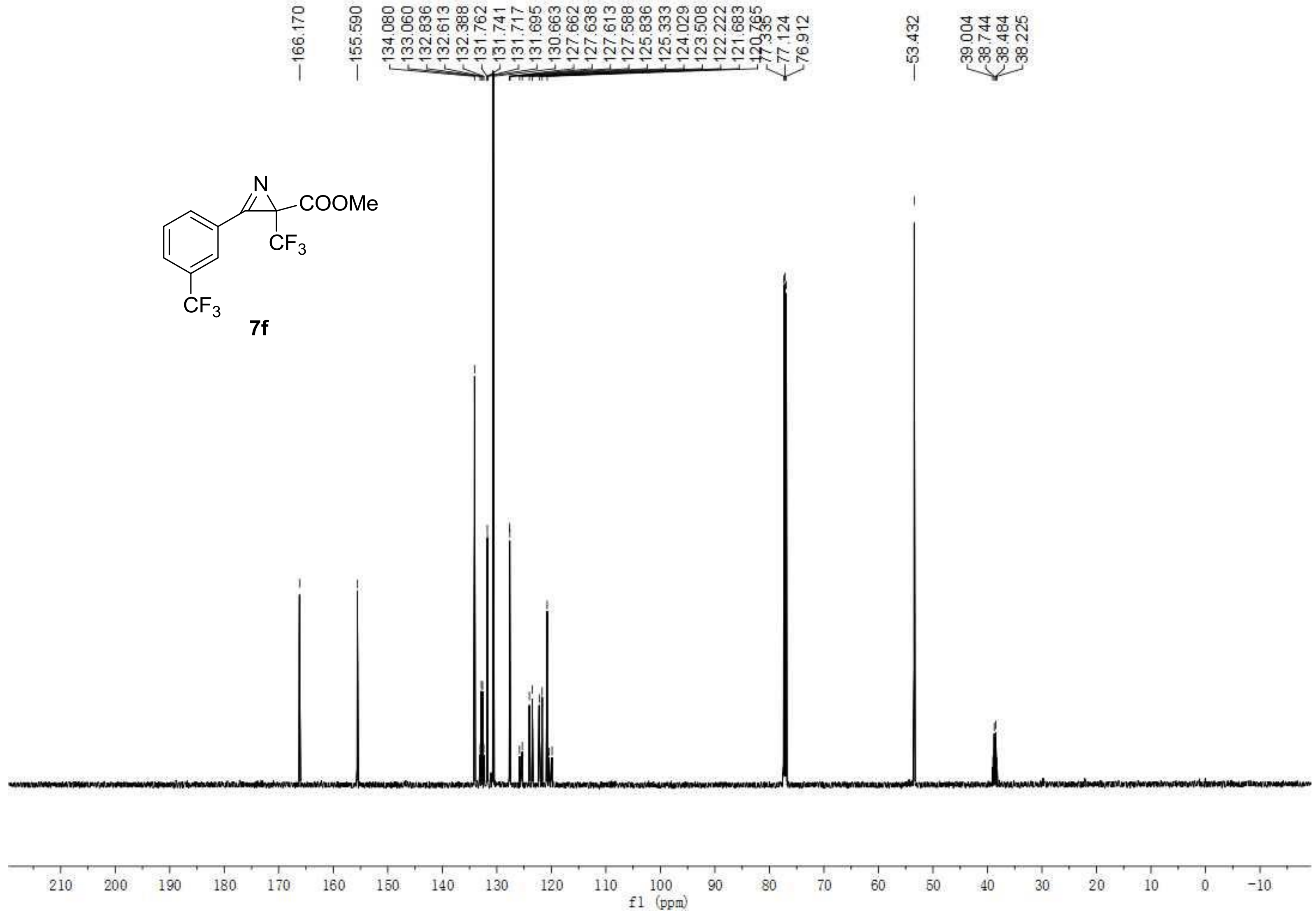
166.170
155.590
134.080
133.060
132.836
132.613
132.388
131.762
131.741
131.717
131.695
130.663
127.662
127.638
127.613
127.588
125.836
125.333
124.029
123.508
122.222
121.683
120.765
77.335
77.124
76.912
53.432
39.004
38.744
38.484
38.225
N
COOMe
CF3
CF3
7f
210 200 190 180 170 160 150 140 130 120 110 100 90 80 70 60 50 40 30 20 10 0 -10
f1 (ppm)

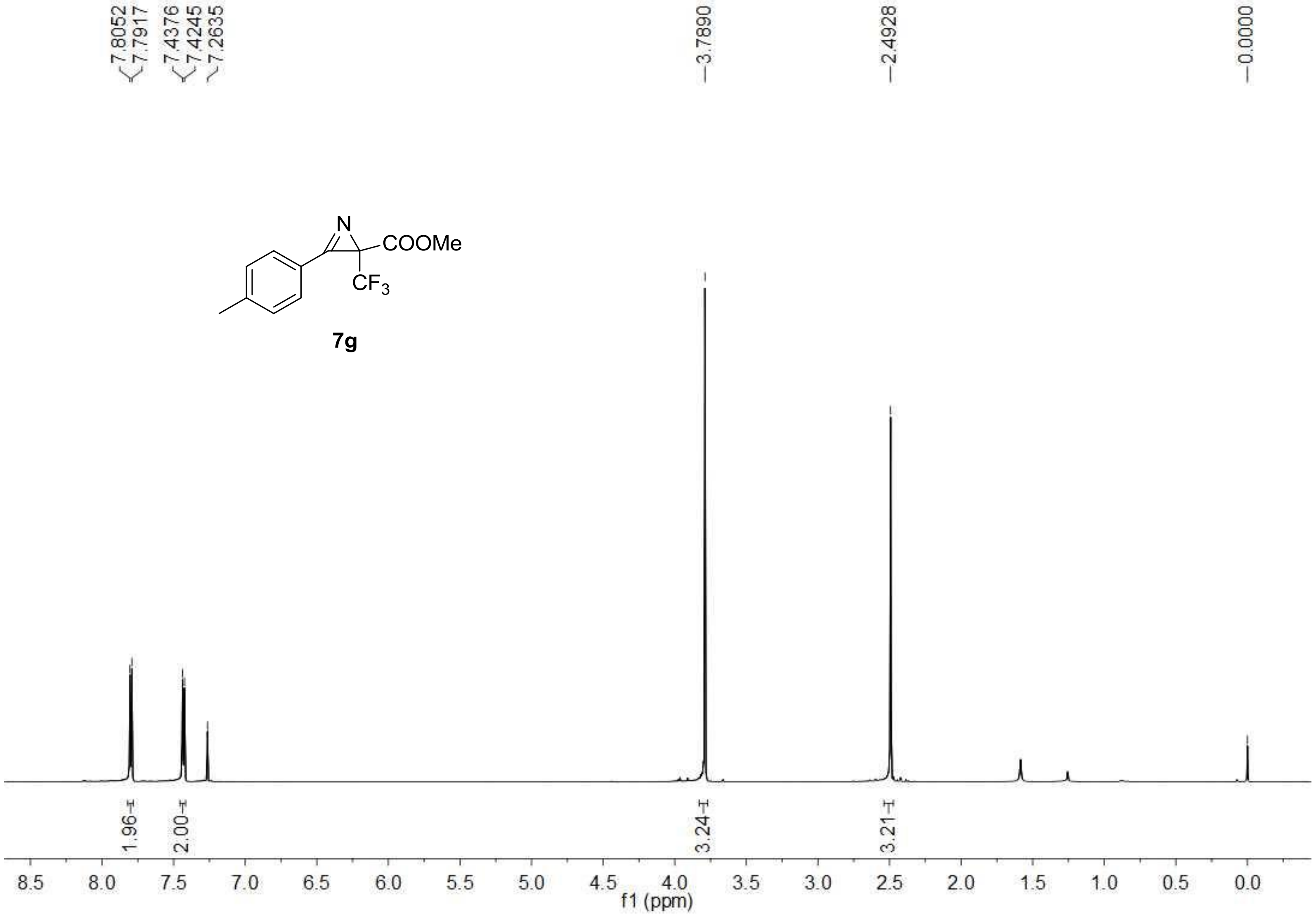
7.8052
7.7917
7.4376
7.4245
7.2635
3.7890
2.4928
0.0000
N
COOMe
CF3
1.96
2.00
3.24
3.21
8.5 8.0 7.5 7.0 6.5 6.0 5.5 5.0 4.5 4.0 3.5 3.0 2.5 2.0 1.5 1.0 0.5 0.0
f1 (ppm)

7g

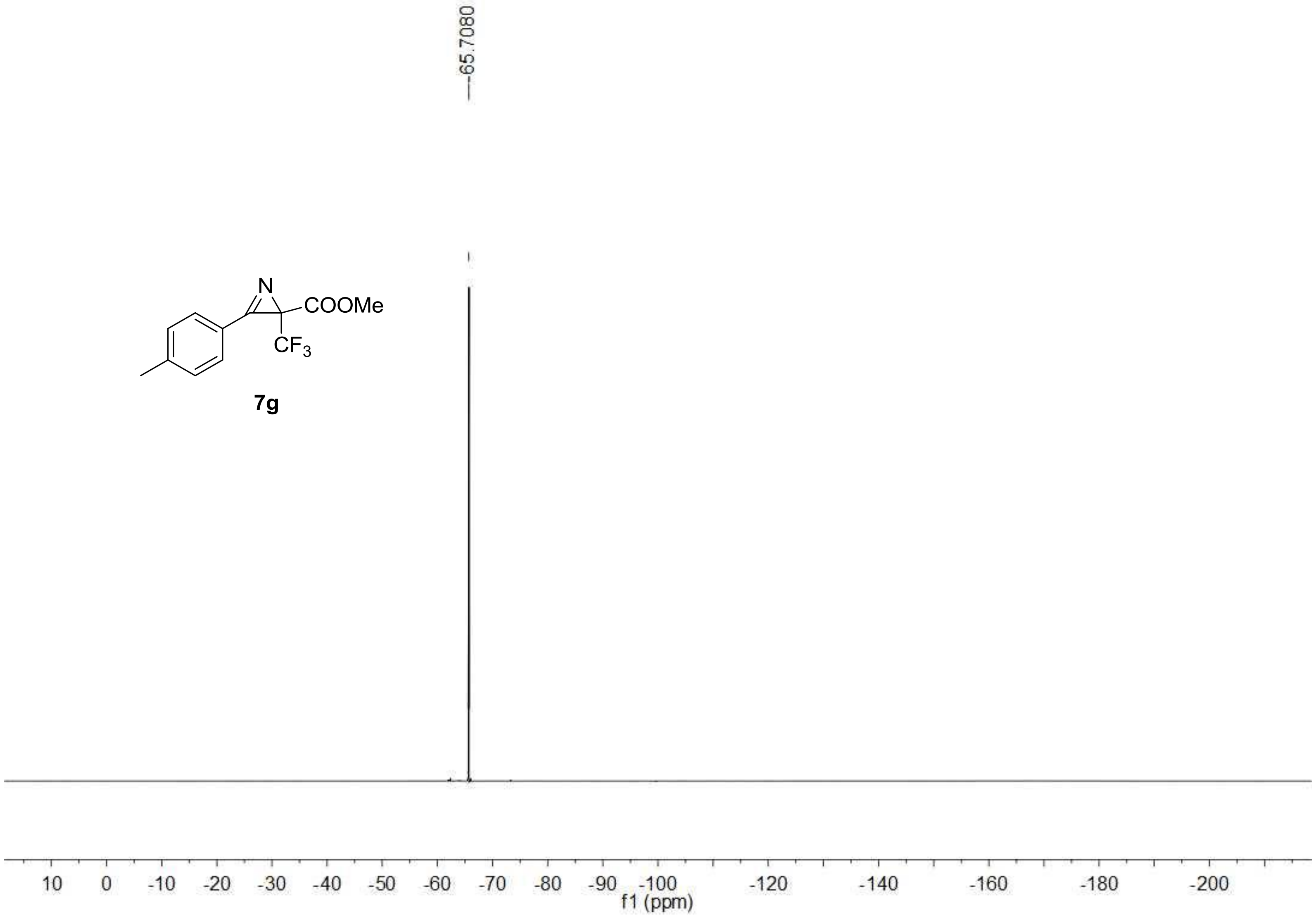
-65.7080
N
COOMe
CF3
7g
10 0 -10 -20 -30 -40 -50 -60 -70 -80 -90 -100 -120 -140 -160 -180 -200
f1 (ppm)

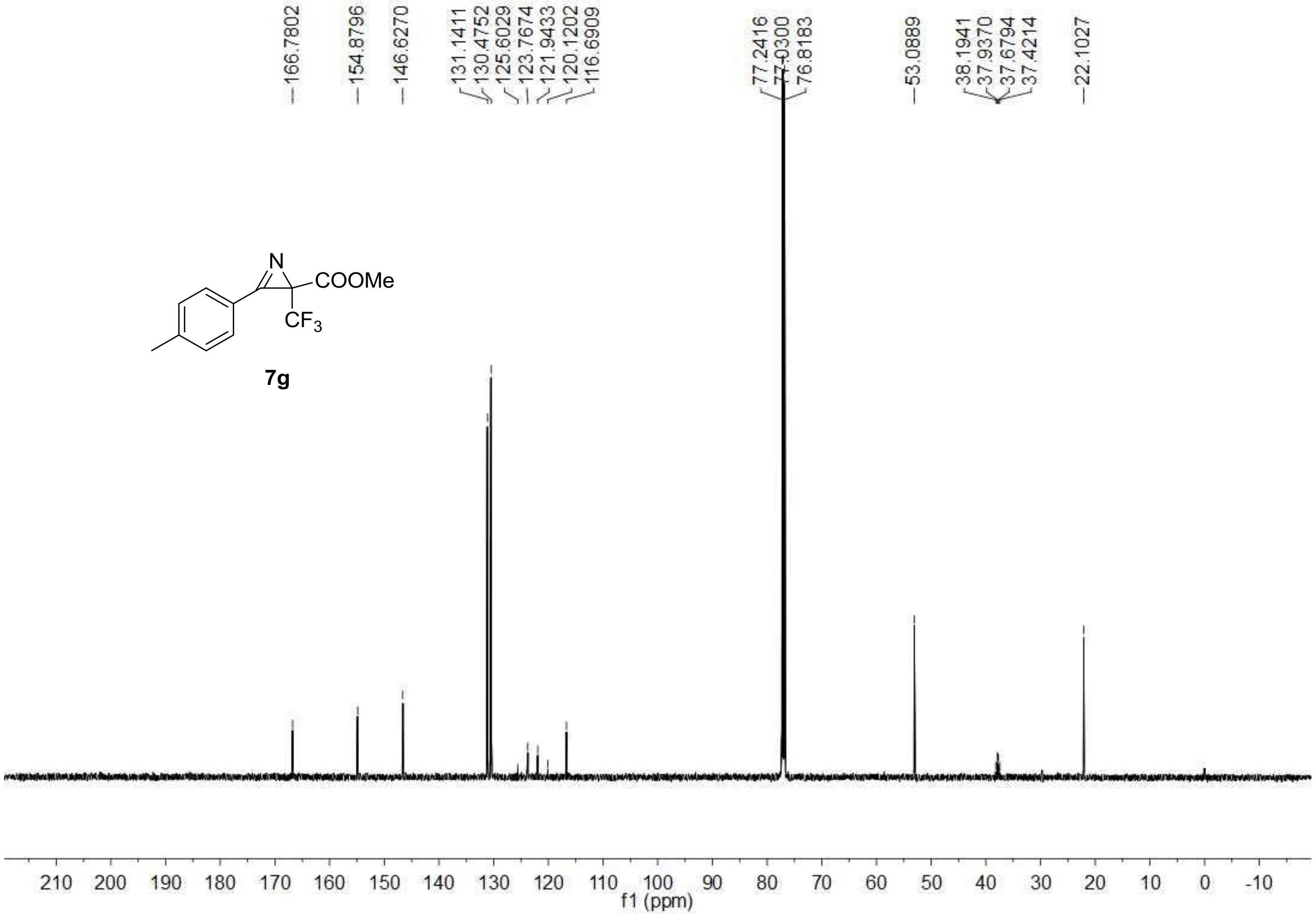
166.7802
154.8796
146.6270
131.1411
130.4752
125.6029
123.7674
121.9433
120.1202
116.6909
77.2416
77.0300
76.8183
53.0889
38.1941
37.9370
37.6794
37.4214
22.1027
N
COOMe
CF3
7g
210 200 190 180 170 160 150 140 130 120 110 100 90 80 70 60 50 40 30 20 10 0 -10
f1 (ppm)

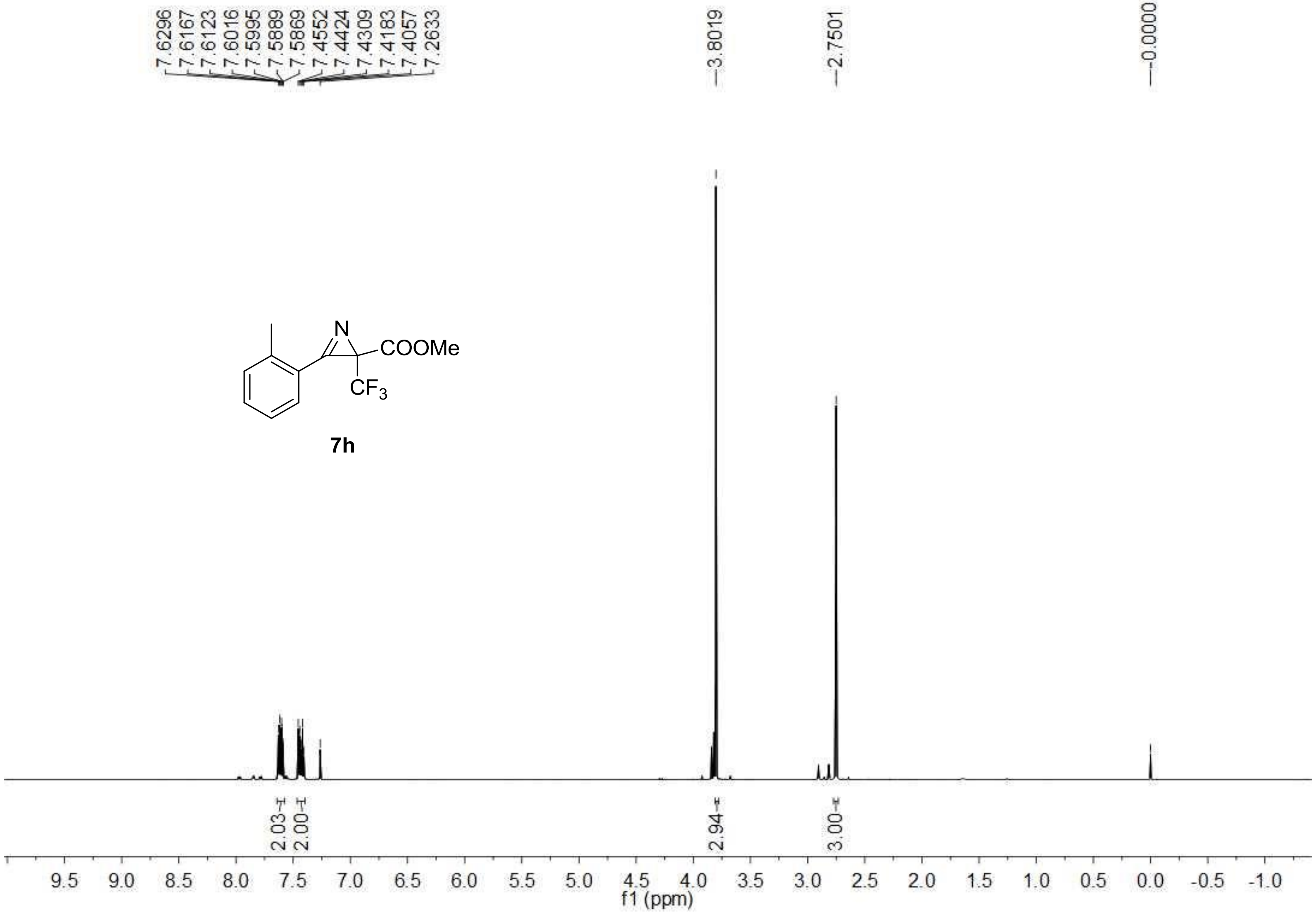
7.6296
7.6167
7.6123
7.6016
7.5995
7.5889
7.5869
7.4552
7.4424
7.4309
7.4183
7.4057
7.2633
3.8019
2.7501
0.0000
N
COOMe
CF3
7h
2.03
2.00
2.94
3.00
9.5 9.0 8.5 8.0 7.5 7.0 6.5 6.0 5.5 5.0 4.5 4.0 3.5 3.0 2.5 2.0 1.5 1.0 0.5 0.0 -0.5 -1.0
f1 (ppm)

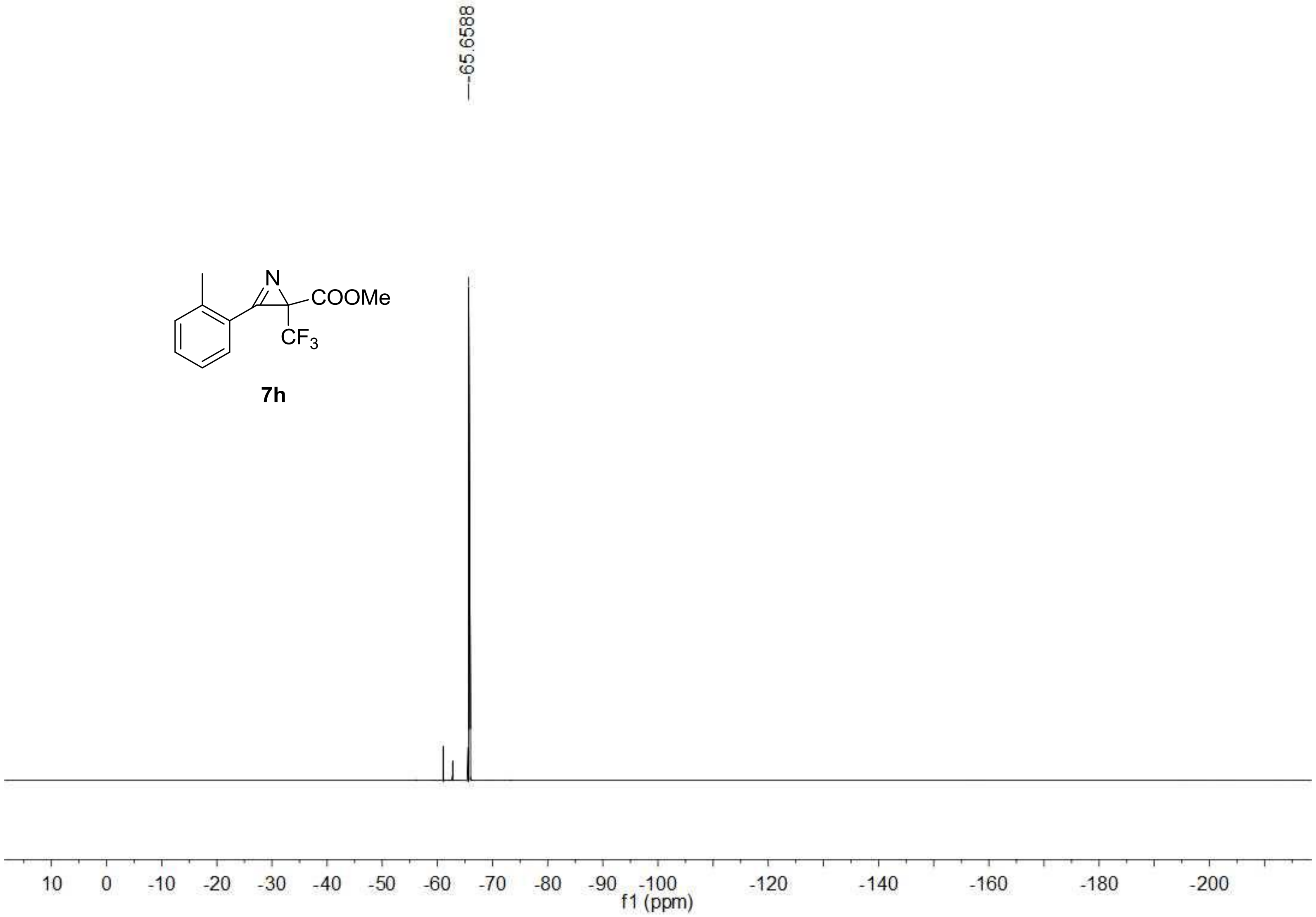
-65.6588
N
COOMe
CF3
7h
10
0
-10
-20
-30
-40
-50
-60
-70
-80
-90
-100
-120
-140
-160
-180
-200
f1 (ppm)

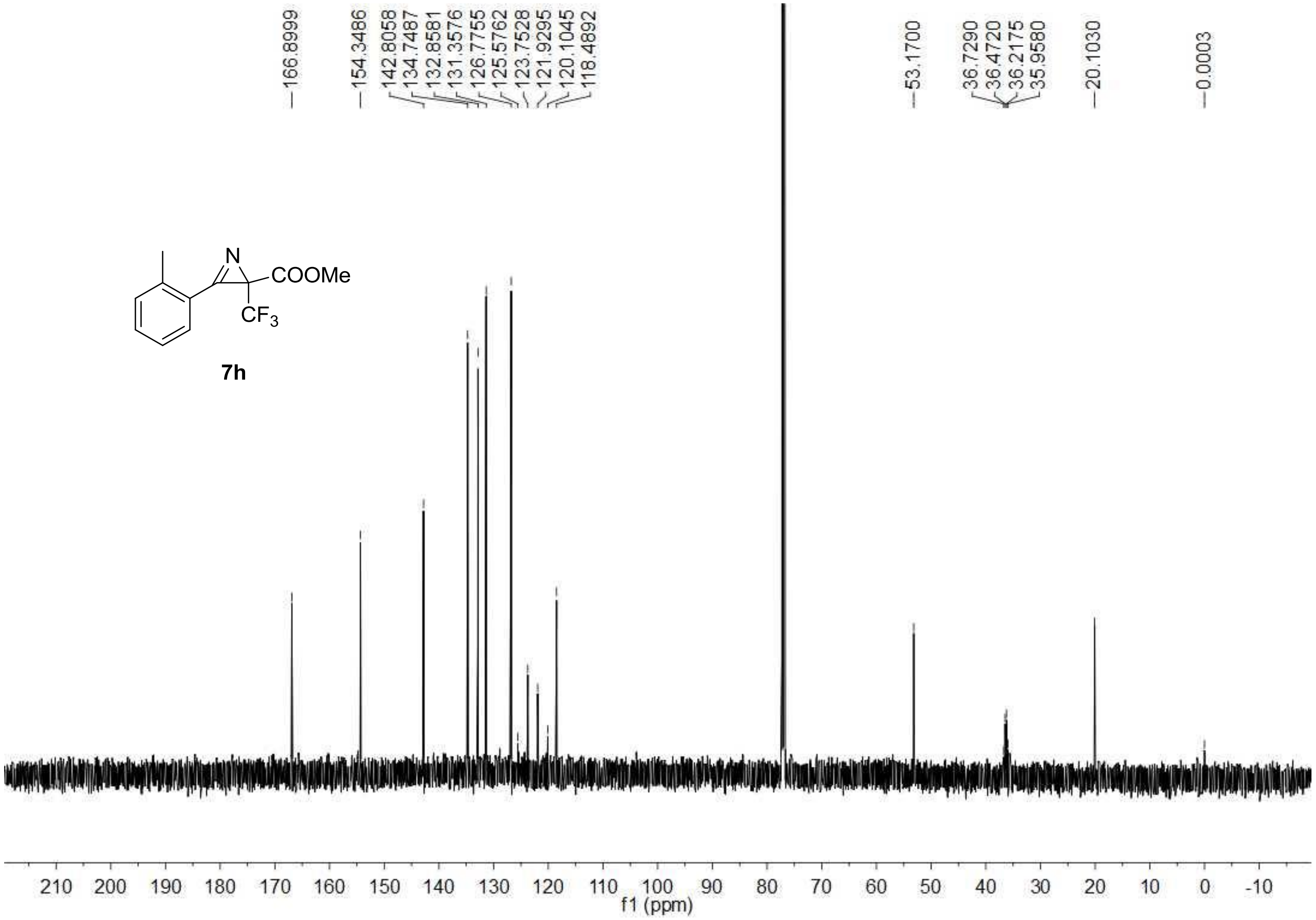
166.8999
154.3486
142.8058
134.7487
132.8581
131.3576
126.7755
125.5762
123.7528
121.9295
120.1045
118.4892
53.1700
36.7290
36.4720
36.2175
35.9580
20.1030
0.0003
N
COOMe
CF_3
7h
210 200 190 180 170 160 150 140 130 120 110 100 90 80 70 60 50 40 30 20 10 0 -10
f1 (ppm)

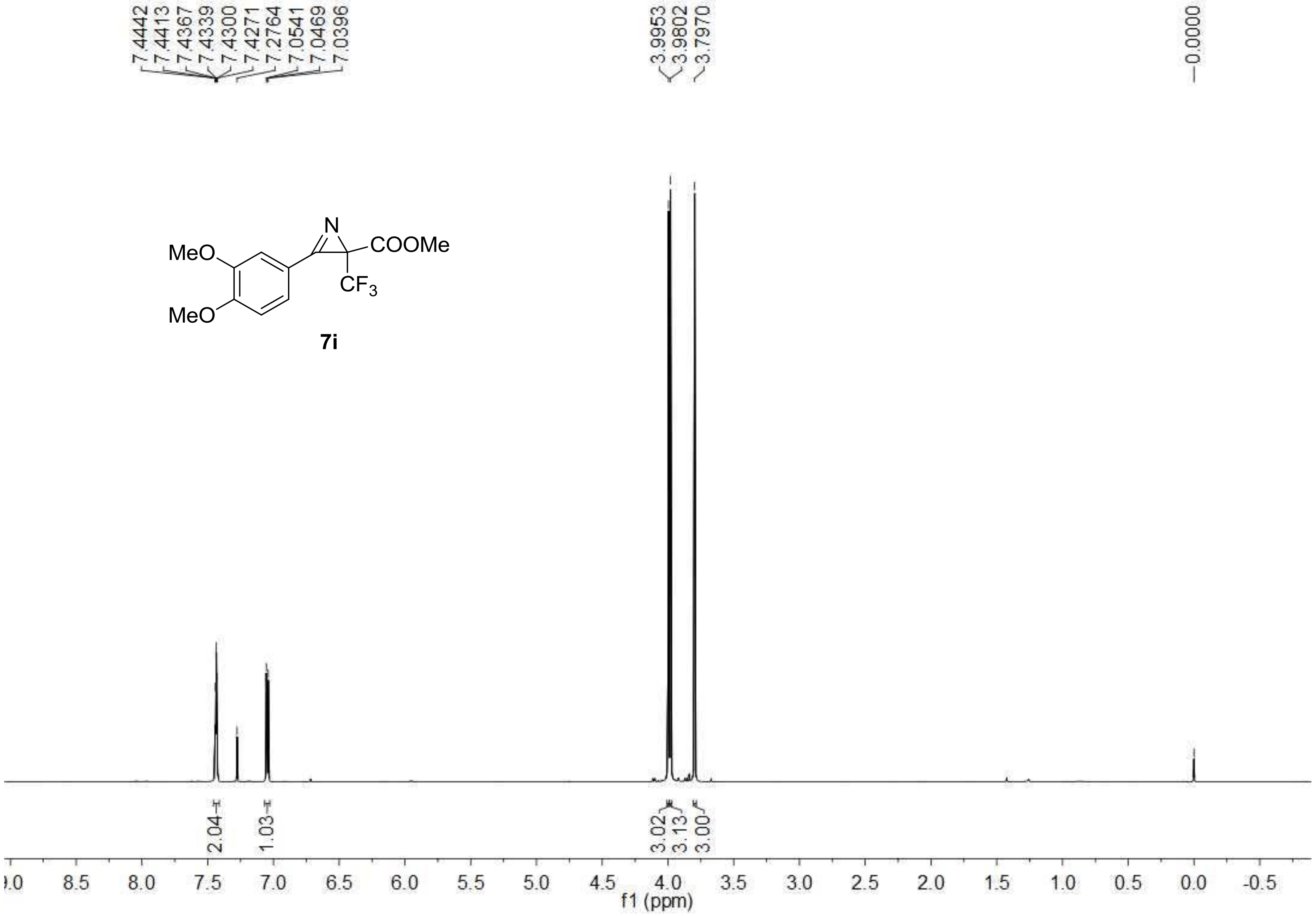
7.4442
7.4413
7.4367
7.4339
7.4300
7.4271
7.2764
7.0541
7.0469
7.0396
3.9953
3.9802
3.7970
0.0000
MeO
MeO
N
COOMe
CF3
7i
2.04
1.03
3.02
3.13
3.00
9.0
8.5
8.0
7.5
7.0
6.5
6.0
5.5
5.0
4.5
4.0
3.5
3.0
2.5
2.0
1.5
1.0
0.5
0.0
-0.5
f1 (ppm)

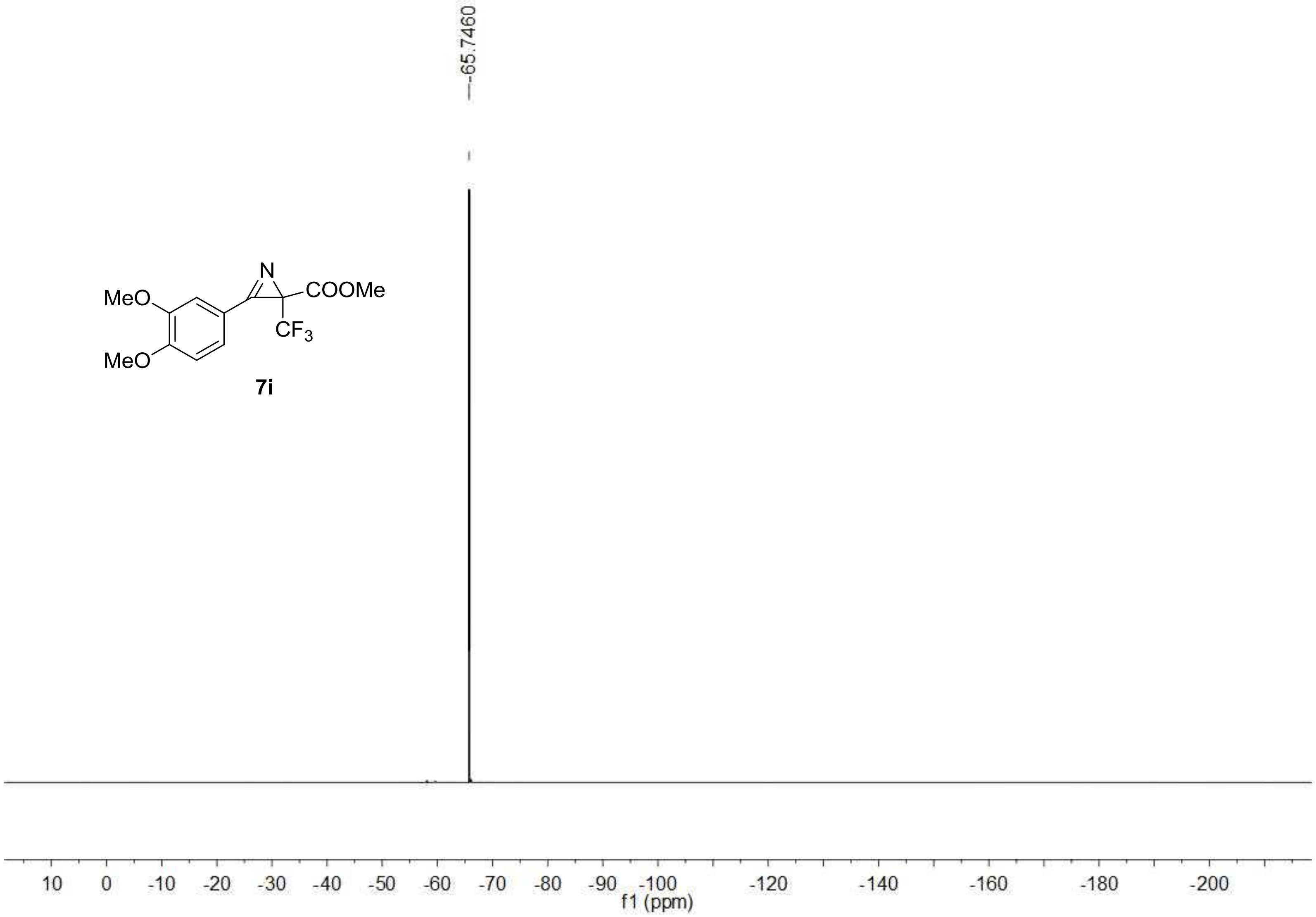
-65.7460
MeO
MeO
N
COOMe
CF3
7i
10 0 -10 -20 -30 -40 -50 -60 -70 -80 -90 -100 -120 -140 -160 -180 -200
f1 (ppm)

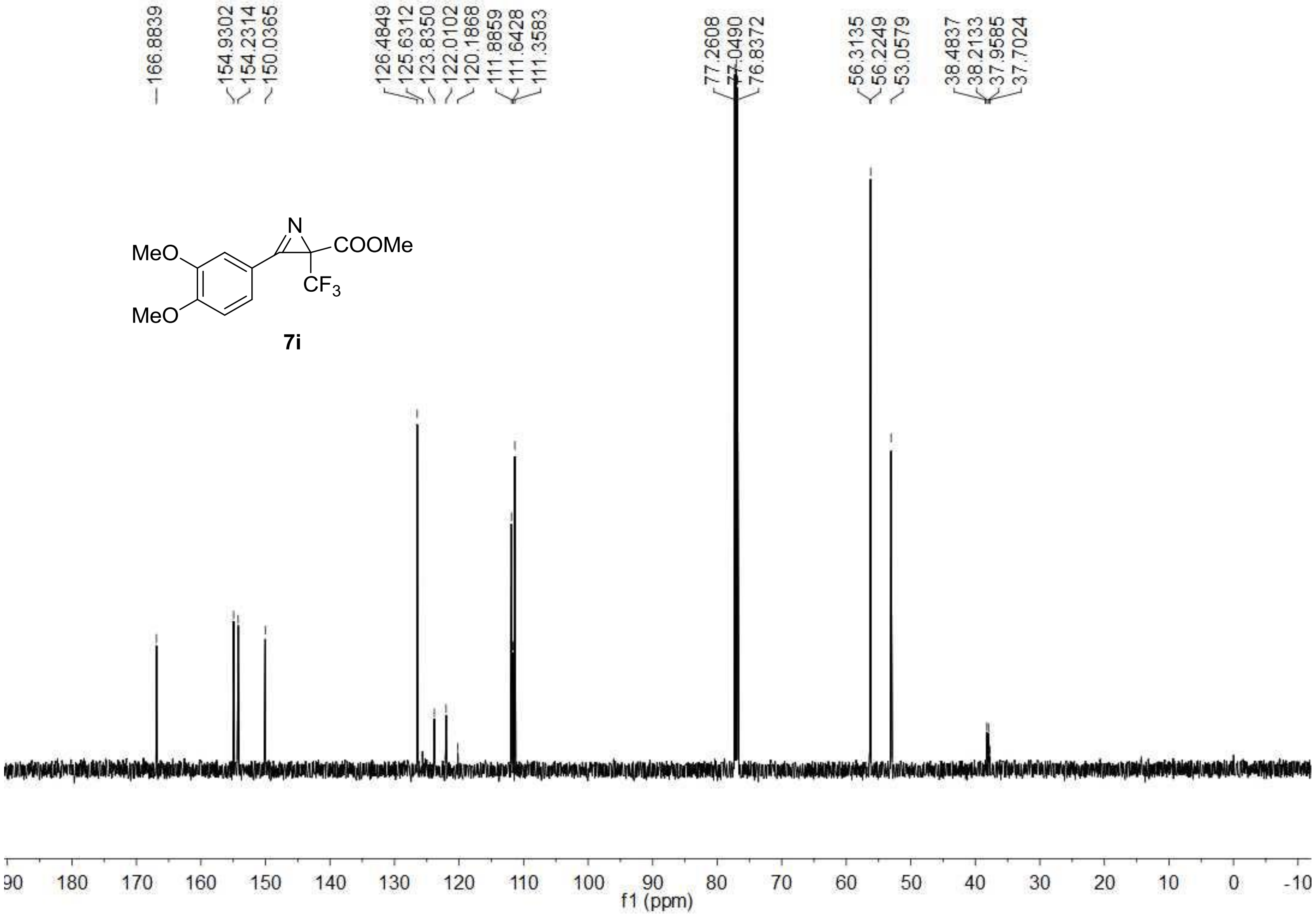
166.8839
154.9302
154.2314
150.0365
126.4849
125.6312
123.8350
122.0102
120.1868
111.8859
111.6428
111.3583
77.2608
77.0490
76.8372
56.3135
56.2249
53.0579
38.4837
38.2133
37.9585
37.7024
MeO
MeO
N
COOMe
CF3
7i
f1 (ppm)

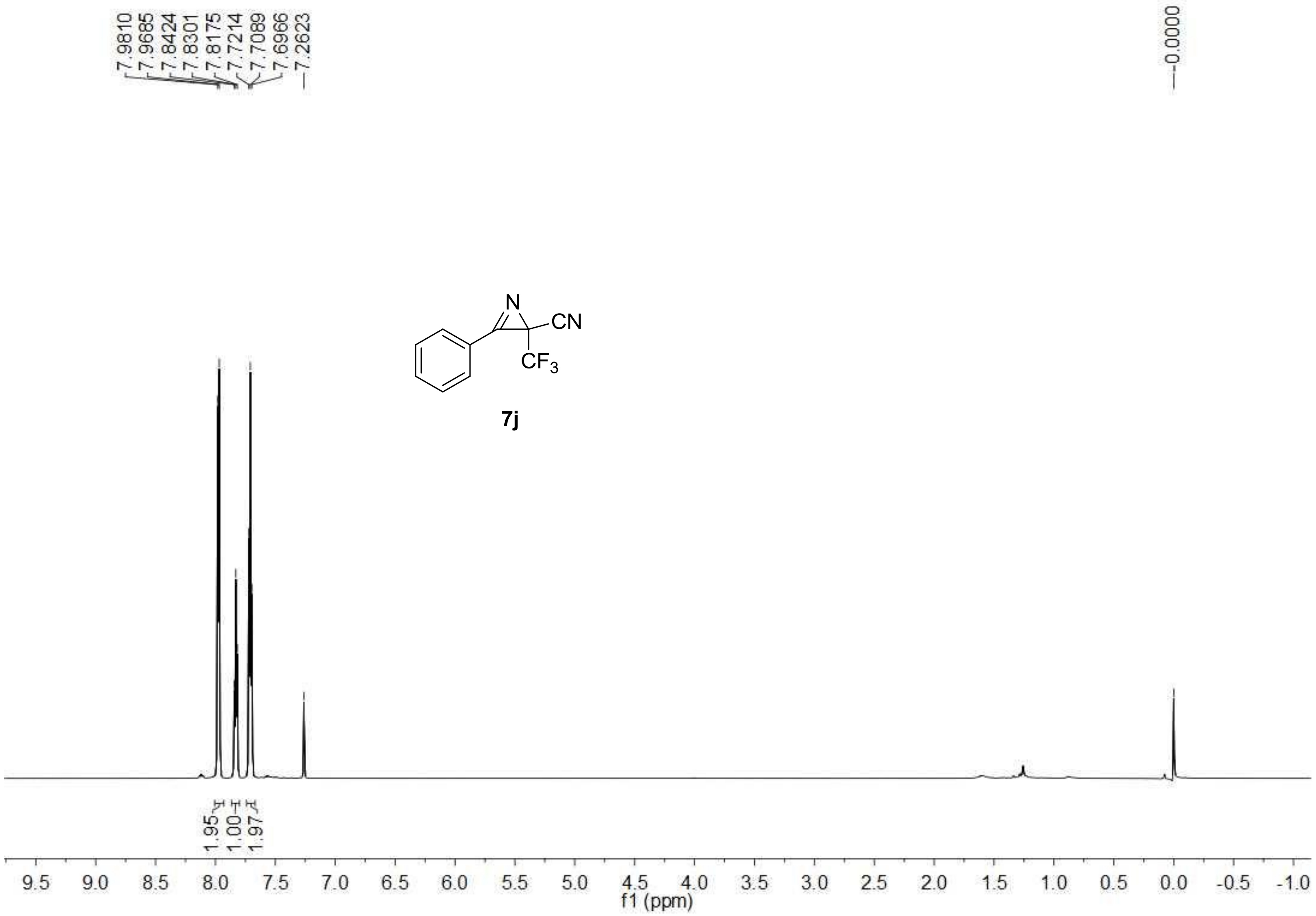
7.9810
7.9685
7.8424
7.8301
7.8175
7.7214
7.7089
7.6966
7.2623
0.0000
N
CN
CF3
7j
1.95
1.00
1.97
9.5 9.0 8.5 8.0 7.5 7.0 6.5 6.0 5.5 5.0 4.5 4.0 3.5 3.0 2.5 2.0 1.5 1.0 0.5 0.0 -0.5 -1.0
f1 (ppm)

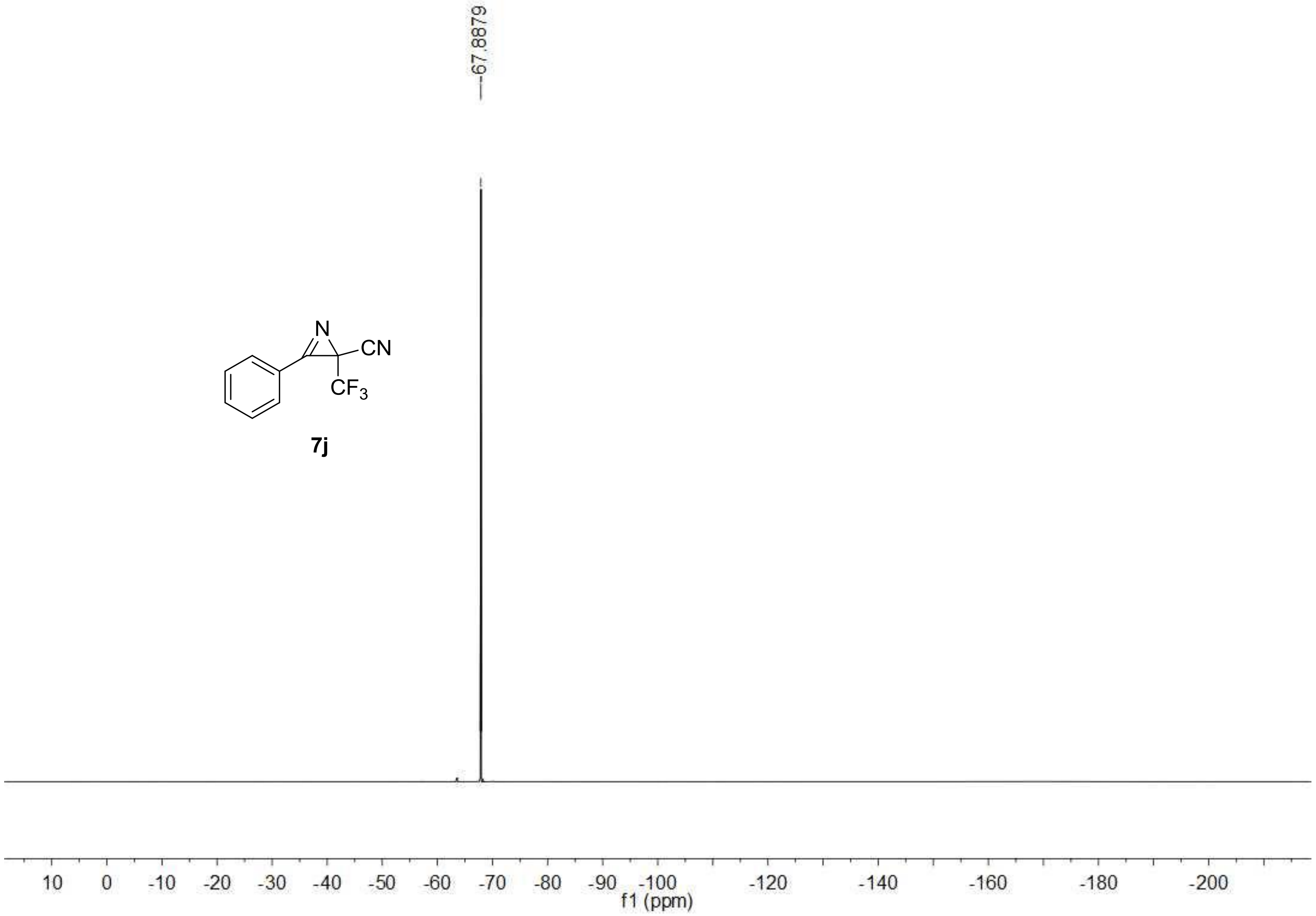

-67.8879
N
CN
CF3
7j
10 0 -10 -20 -30 -40 -50 -60 -70 -80 -90 -100 -120 -140 -160 -180 -200
f1 (ppm)

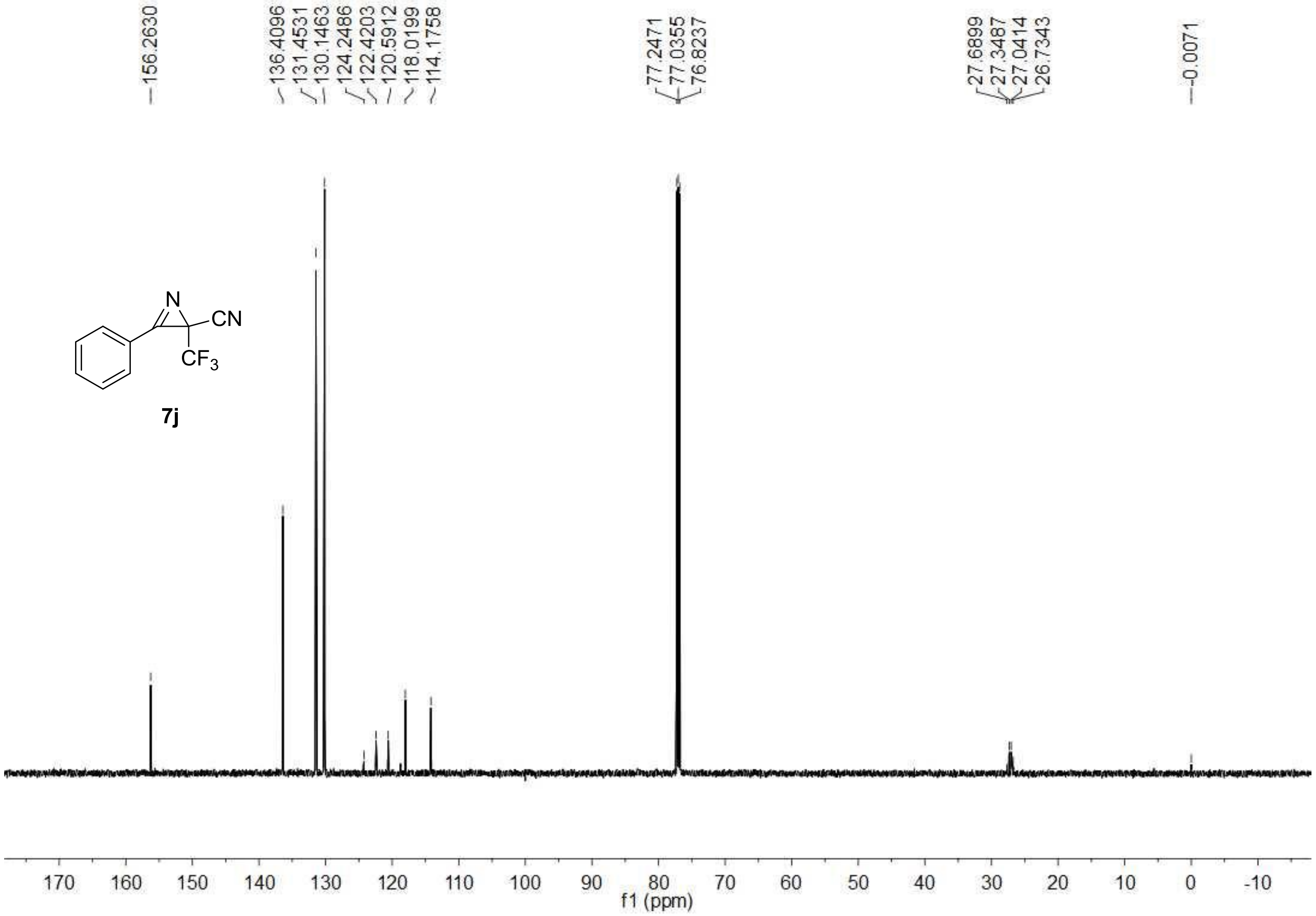
156.2630
136.4096
131.4531
130.1463
124.2486
122.4203
120.5912
118.0199
114.1758
77.2471
77.0355
76.8237
27.6899
27.3487
27.0414
26.7343
-0.0071
N
CN
CF3
7j
170 160 150 140 130 120 110 100 90 80 70 60 50 40 30 20 10 0 -10
f1 (ppm)

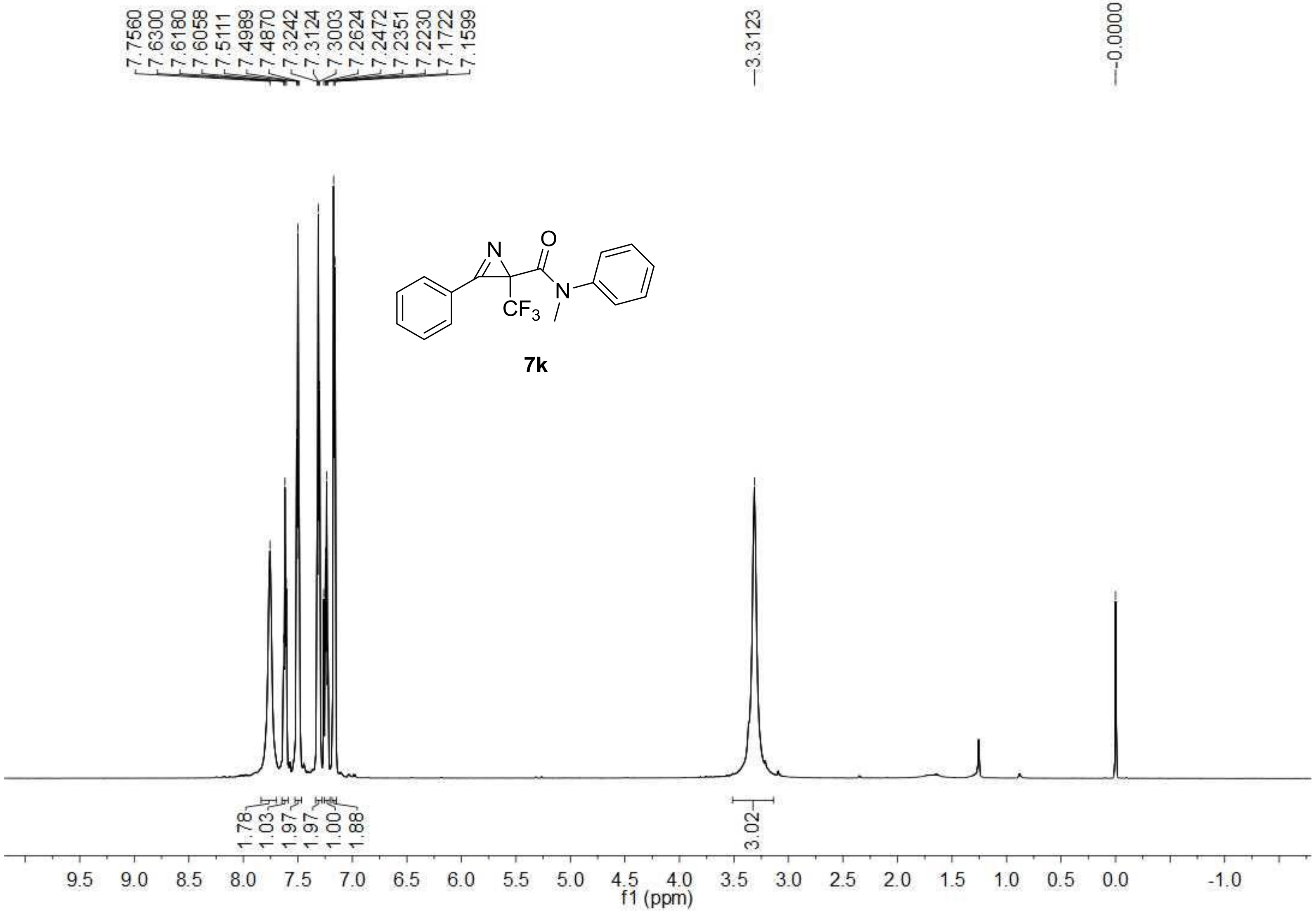
7.7560
7.6300
7.6180
7.6058
7.5111
7.4989
7.4870
7.3242
7.3124
7.3003
7.2624
7.2472
7.2351
7.2230
7.1722
7.1599
3.3123
0.0000
N
O
N
CF3
7k
1.78
1.03
1.97
1.97
1.00
1.88
3.02
9.5 9.0 8.5 8.0 7.5 7.0 6.5 6.0 5.5 5.0 4.5 4.0 3.5 3.0 2.5 2.0 1.5 1.0 0.5 0.0 -1.0
f1 (ppm)

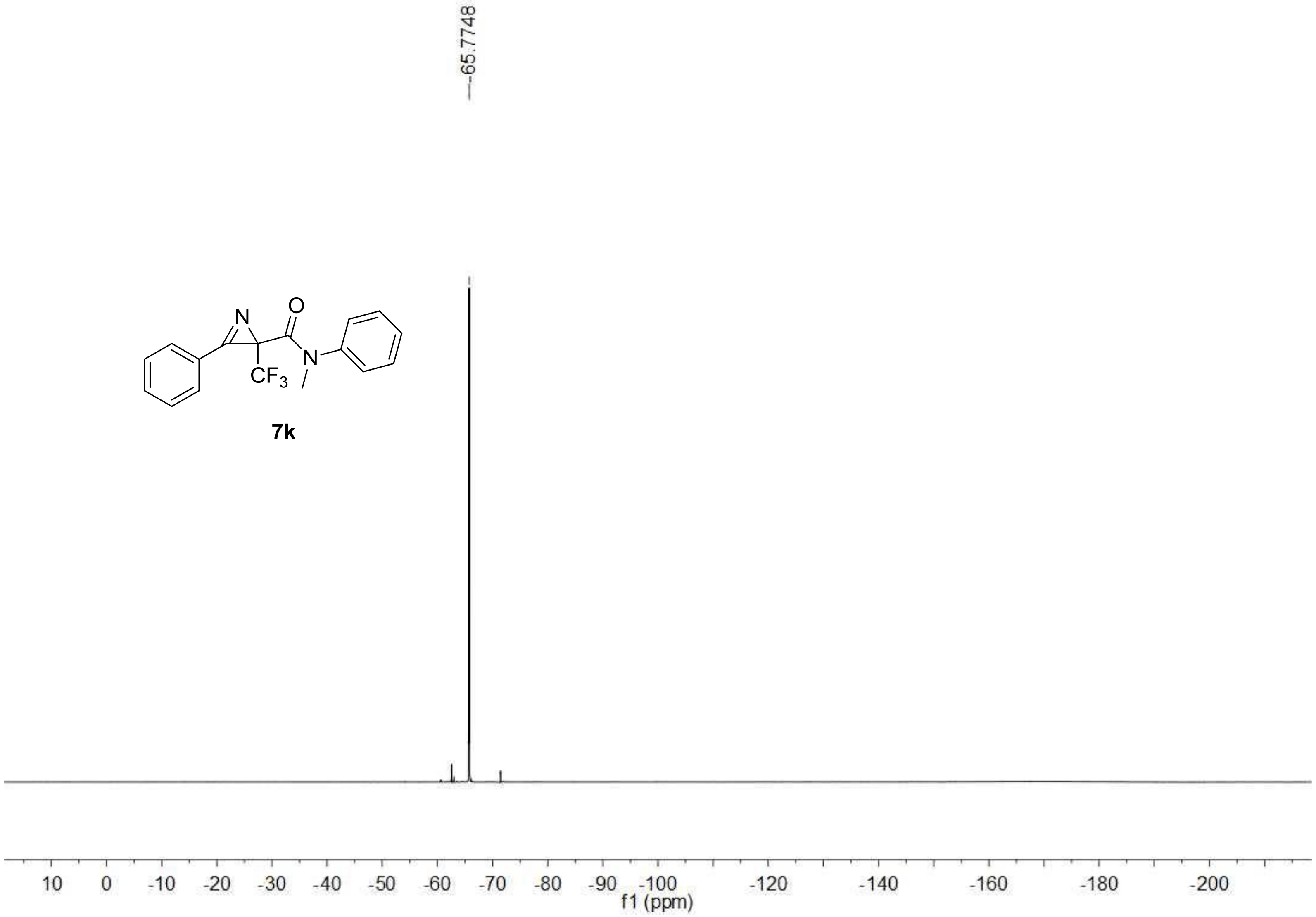

-65.7748
N
O
CF3
N
10
0
-10
-20
-30
-40
-50
-60
-70
-80
-90
-100
-120
-140
-160
-180
-200
f1 (ppm)

7k

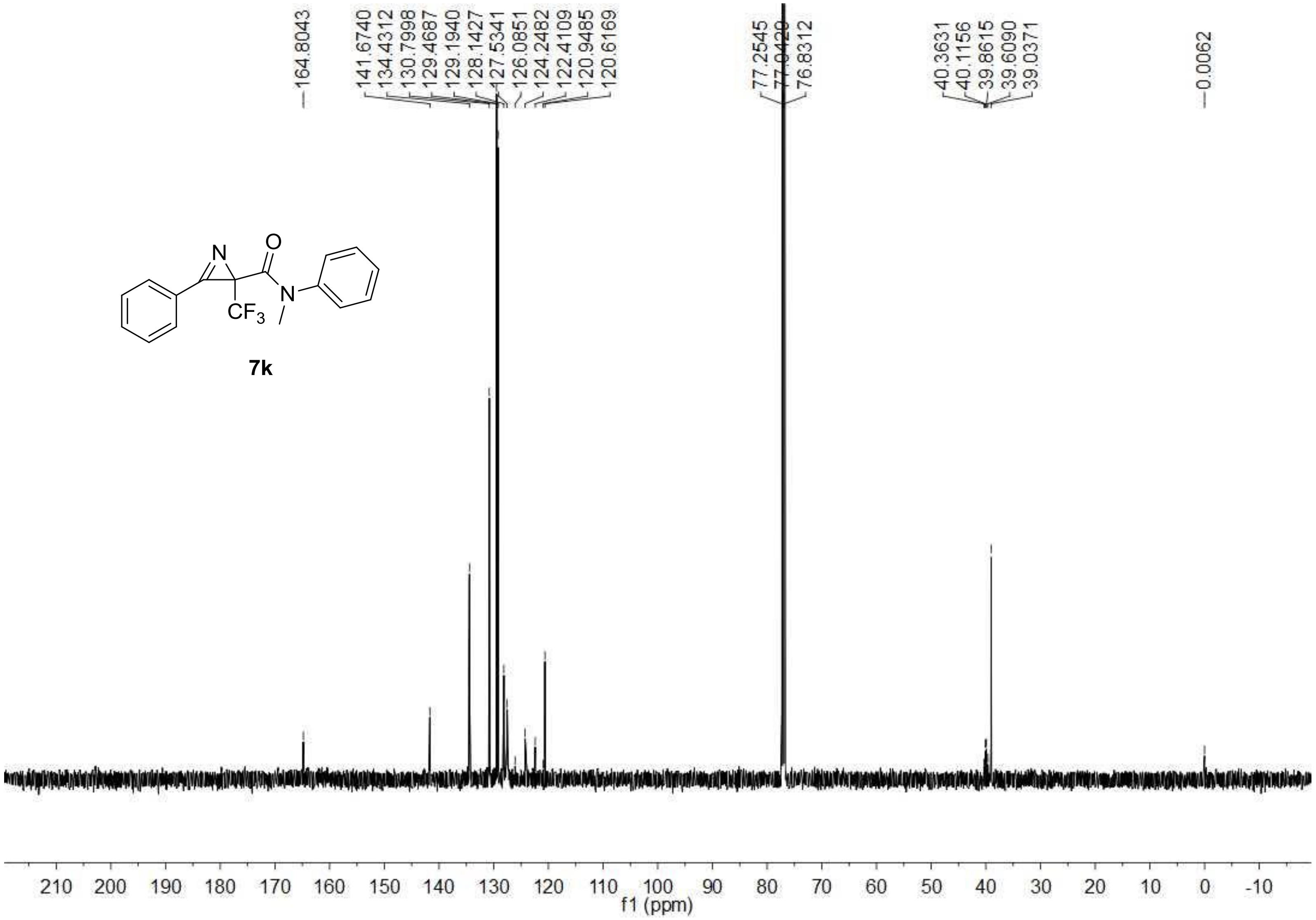

164.8043
141.6740
134.4312
130.7998
129.4687
129.1940
128.1427
127.5341
126.0851
124.2482
122.4109
120.9485
120.6169
77.2545
77.0429
76.8312
40.3631
40.1156
39.8615
39.6090
39.0371
-0.0062
N
O
N
CF3
7k
210 200 190 180 170 160 150 140 130 120 110 100 90 80 70 60 50 40 30 20 10 0 -10
f1 (ppm)

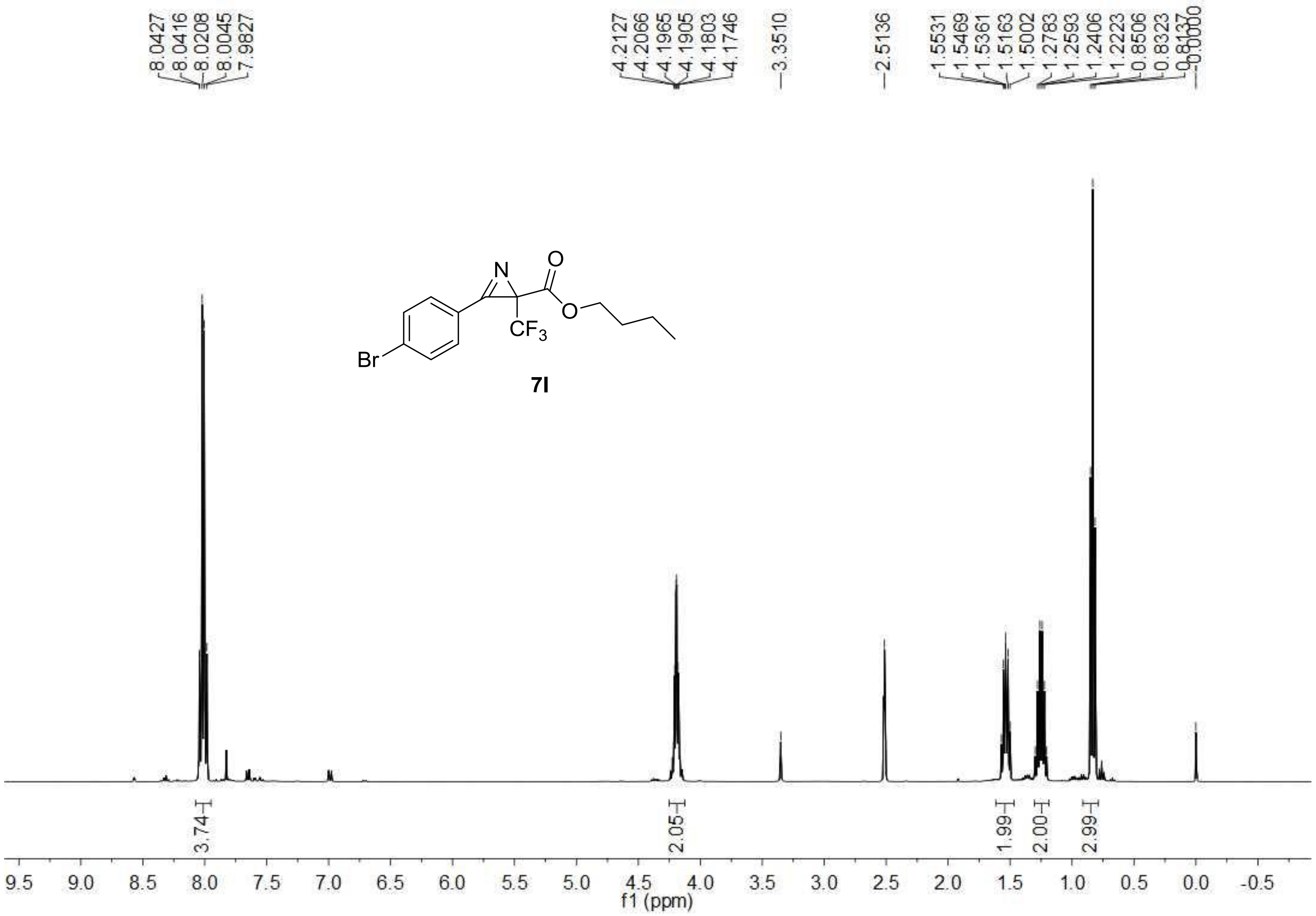
8.0427
8.0416
8.0208
8.0045
7.9827
4.2127
4.2066
4.1965
4.1905
4.1803
4.1746
3.3510
2.5136
1.5531
1.5469
1.5361
1.5163
1.5002
1.2783
1.2593
1.2406
1.2223
0.8506
0.8323
0.8137
0.0000
N
O
O
CF3
Br
7l
3.74
2.05
1.99
2.00
2.99
9.5 9.0 8.5 8.0 7.5 7.0 6.5 6.0 5.5 5.0 4.5 4.0 3.5 3.0 2.5 2.0 1.5 1.0 0.5 0.0 -0.5
f1 (ppm)

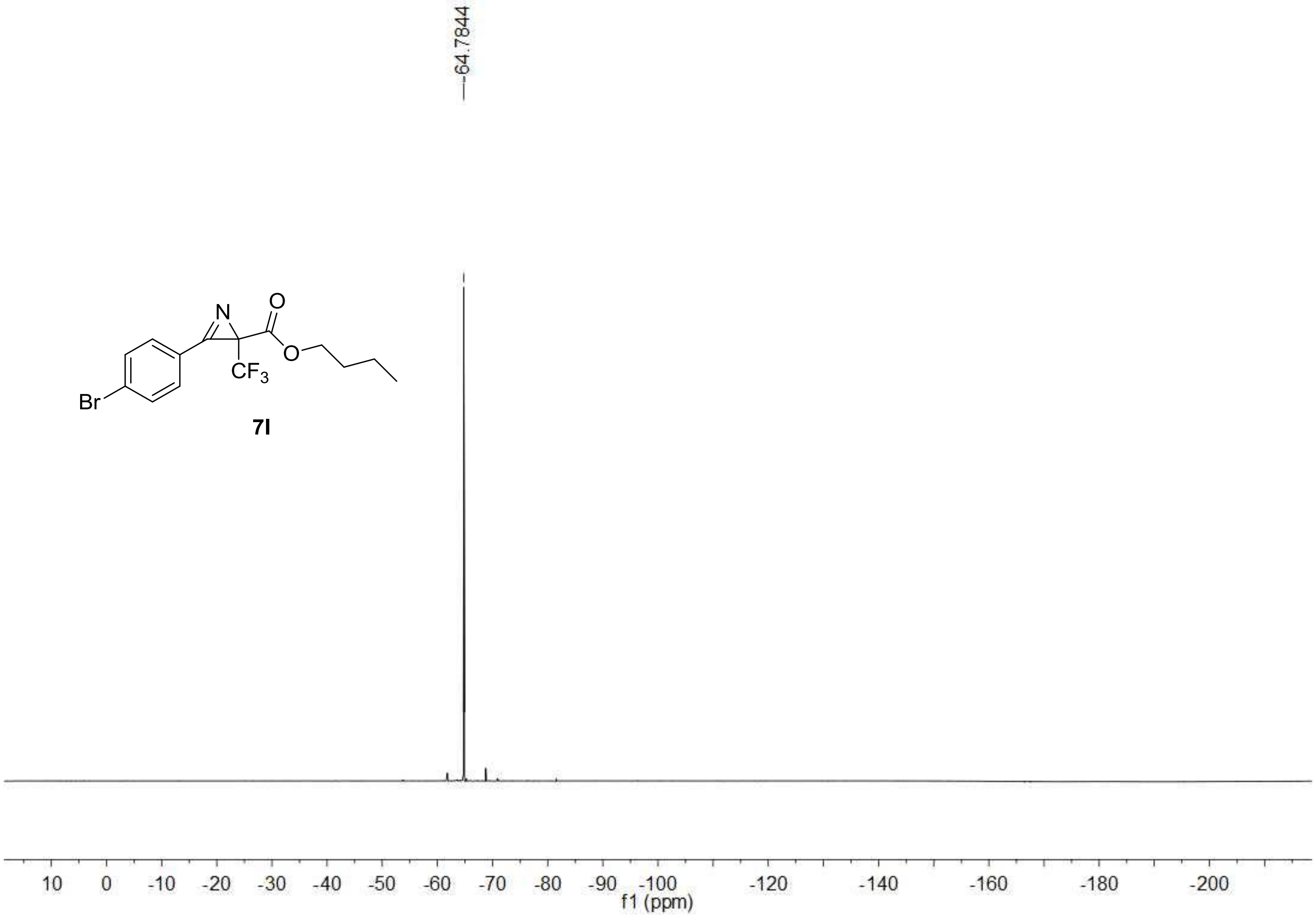
-64.7844
N
O
O
CF3
Br
7l
10 0 -10 -20 -30 -40 -50 -60 -70 -80 -90 -100 -120 -140 -160 -180 -200
f1 (ppm)

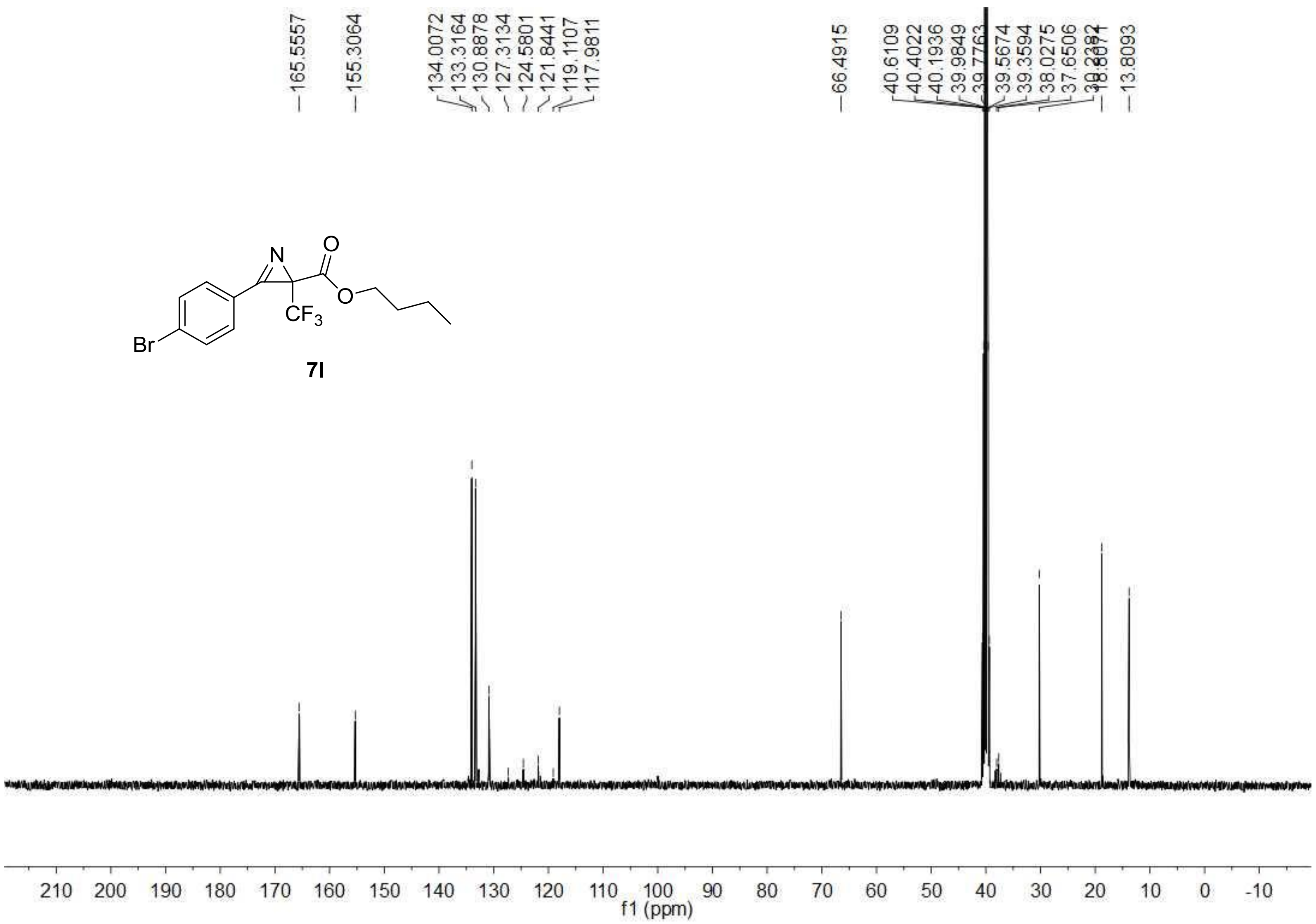
165.5557
155.3064
134.0072
133.3164
130.8878
127.3134
124.5801
121.8441
119.1107
117.9811
66.4915
40.6109
40.4022
40.1936
39.9849
39.7763
39.5674
39.3594
38.0275
37.6506
13.8093
N
O
O
CF3
Br
7l
210
200
190
180
170
160
150
140
130
120
110
100
90
80
70
60
50
40
30
20
10
0
-10
f1 (ppm)

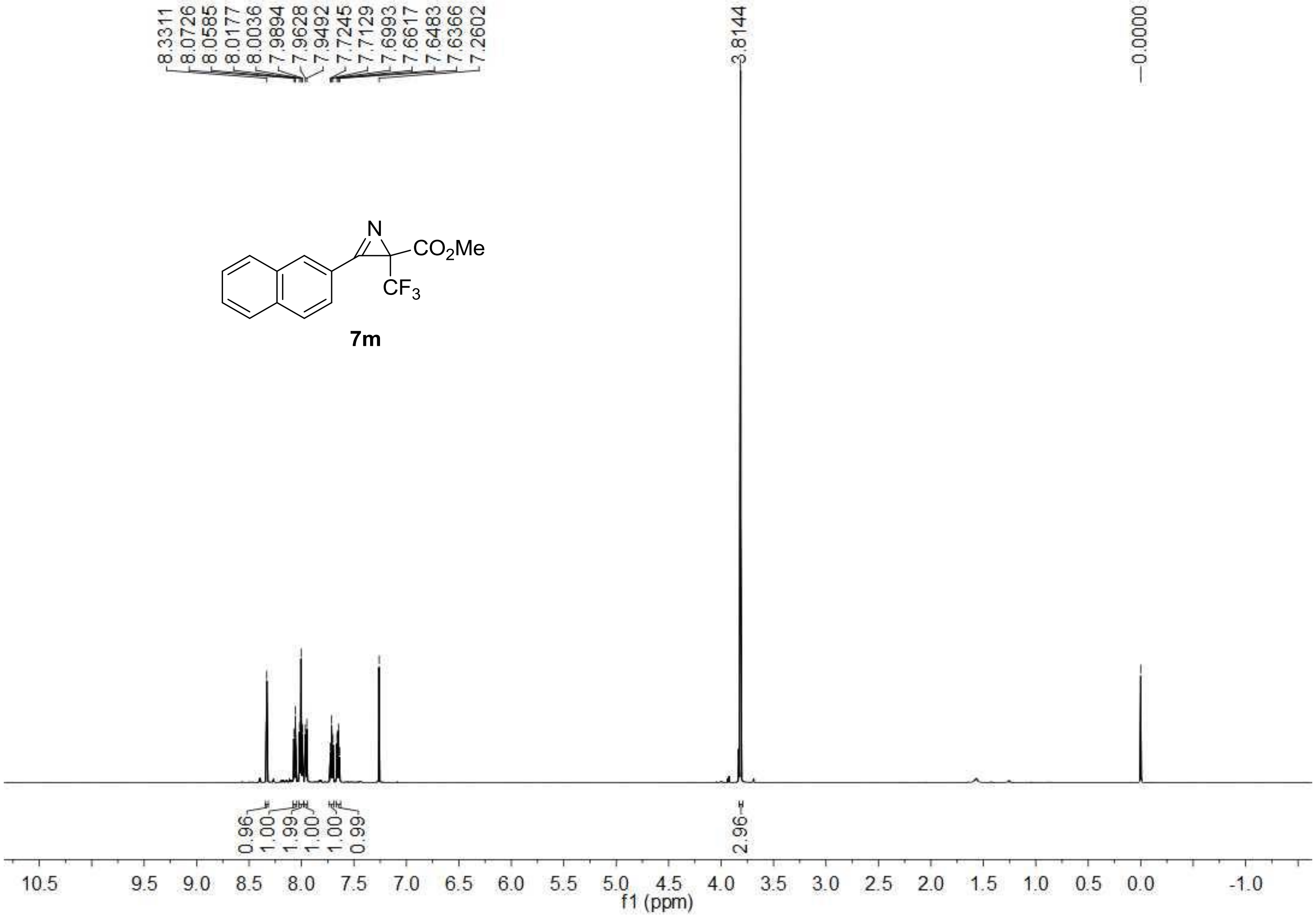
8.3311
8.0726
8.0585
8.0177
8.0036
7.9894
7.9628
7.9492
7.7245
7.7129
7.6993
7.6617
7.6483
7.6366
7.2602
3.8144
0.0000
N
CO2Me
CF3
7m
0.96
1.00
1.99
1.00
1.00
0.99
2.96
10.5 9.5 9.0 8.5 8.0 7.5 7.0 6.5 6.0 5.5 5.0 4.5 4.0 3.5 3.0 2.5 2.0 1.5 1.0 0.5 0.0 -1.0
f1 (ppm)

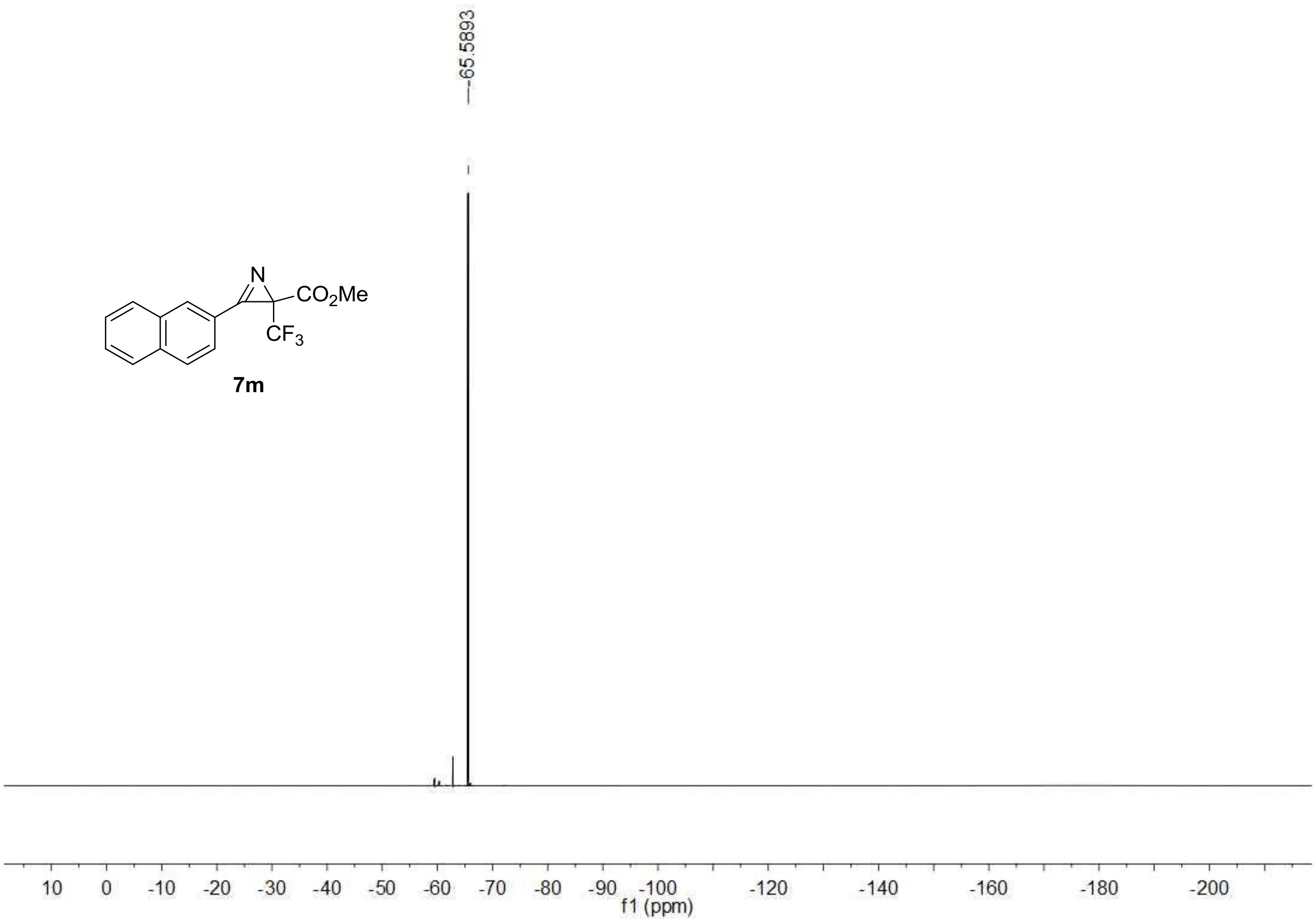
-65.5893
N
CO2Me
CF3
7m
10 0 -10 -20 -30 -40 -50 -60 -70 -80 -90 -100 -120 -140 -160 -180 -200
f1 (ppm)

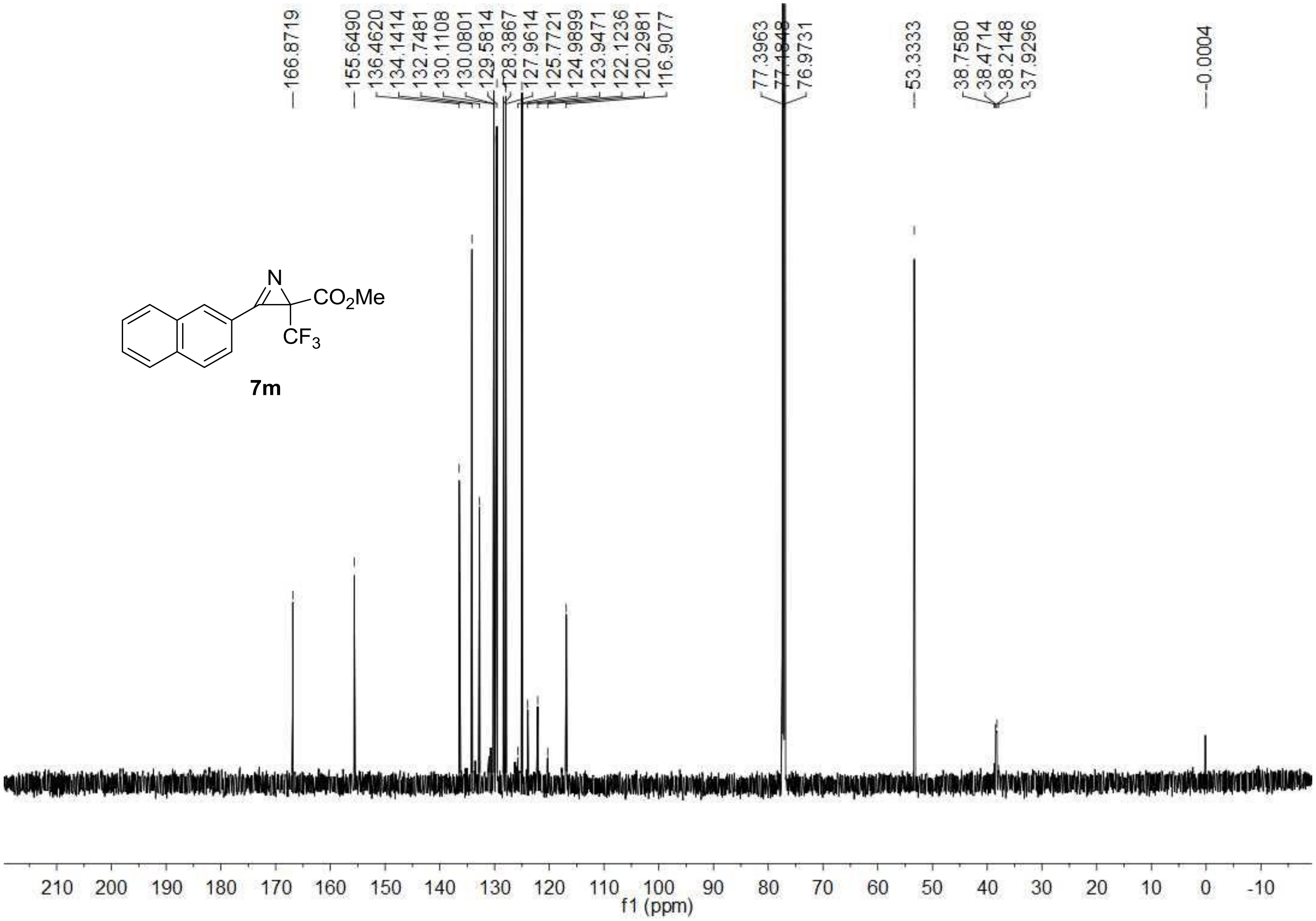
166.8719
155.6490
136.4620
134.1414
132.7481
130.1108
130.0801
129.5814
128.3867
127.9614
125.7721
124.9899
123.9471
122.1236
120.2981
116.9077
77.3963
77.1848
76.9731
53.3333
38.7580
38.4714
38.2148
37.9296
-0.0004
210 200 190 180 170 160 150 140 130 120 110 100 90 80 70 60 50 40 30 20 10 0 -10
f1 (ppm)
N
CO_2Me
CF_3

7m

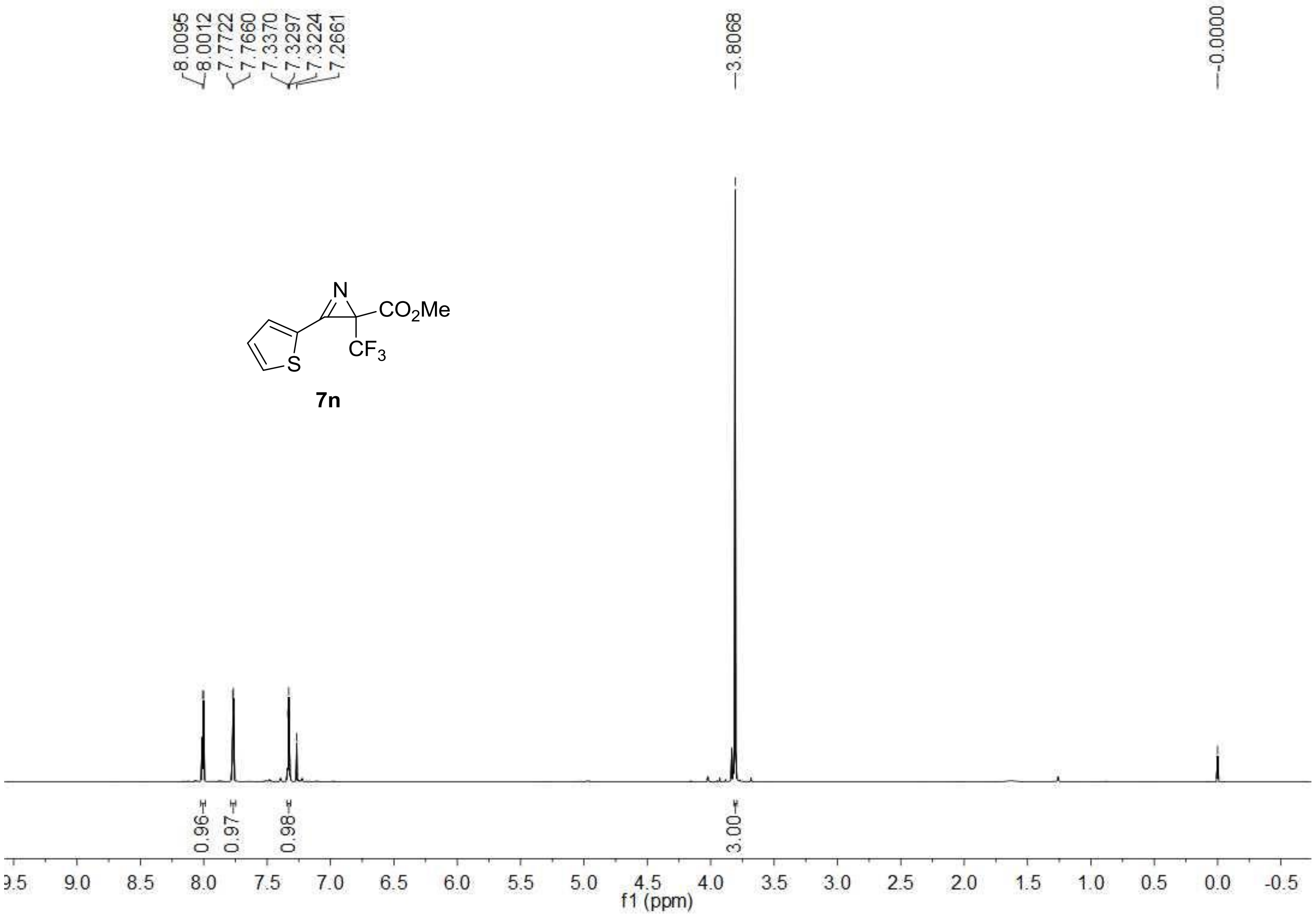
8.0095
8.0012
7.7722
7.7660
7.3370
7.3297
7.3224
7.2661
3.8068
0.0000
N
CO2Me
CF3
S
7n
0.96
0.97
0.98
3.00
9.5 9.0 8.5 8.0 7.5 7.0 6.5 6.0 5.5 5.0 4.5 4.0 3.5 3.0 2.5 2.0 1.5 1.0 0.5 0.0 -0.5
f1 (ppm)

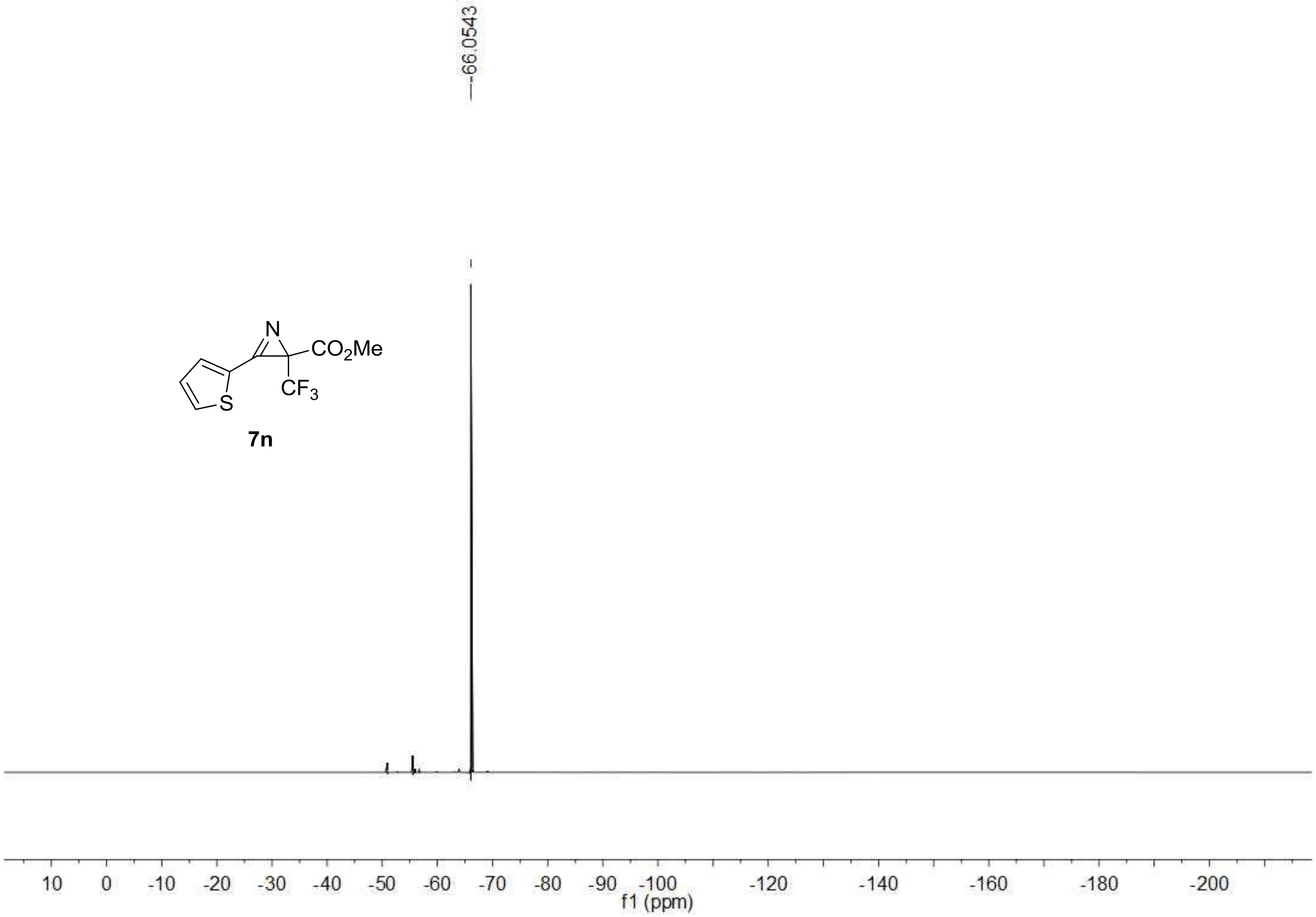

-66.0543
N
CO2Me
CF3
S
7n
10 0 -10 -20 -30 -40 -50 -60 -70 -80 -90 -100 -120 -140 -160 -180 -200
f1 (ppm)

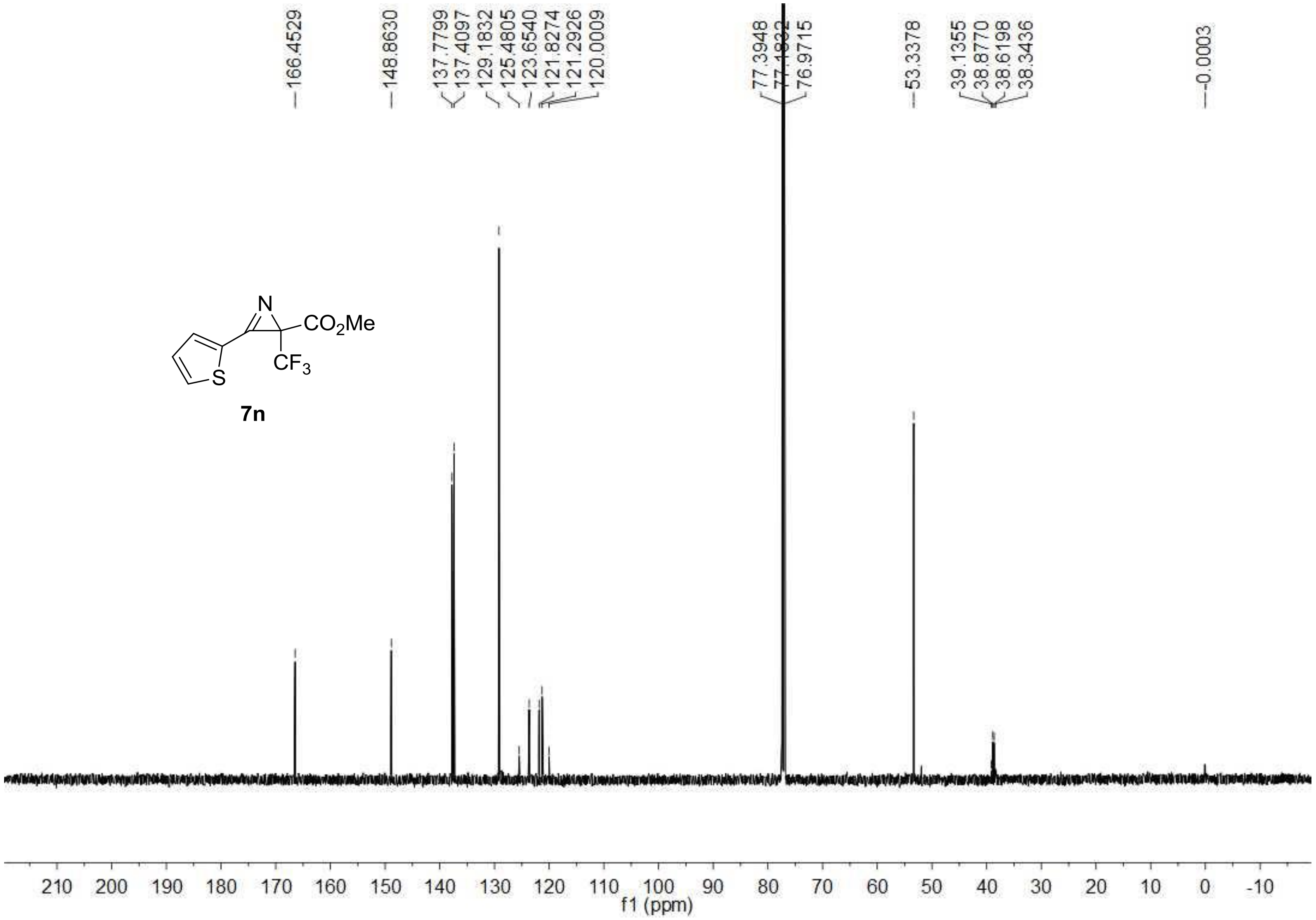

166.4529
148.8630
137.7799
137.4097
129.1832
125.4805
123.6540
121.8274
121.2926
120.0009
77.3948
76.9715
53.3378
39.1355
38.8770
38.6198
38.3436
0.0003
N
CO2Me
CF3
S
7n
210 200 190 180 170 160 150 140 130 120 110 100 90 80 70 60 50 40 30 20 10 0 -10
f1 (ppm)

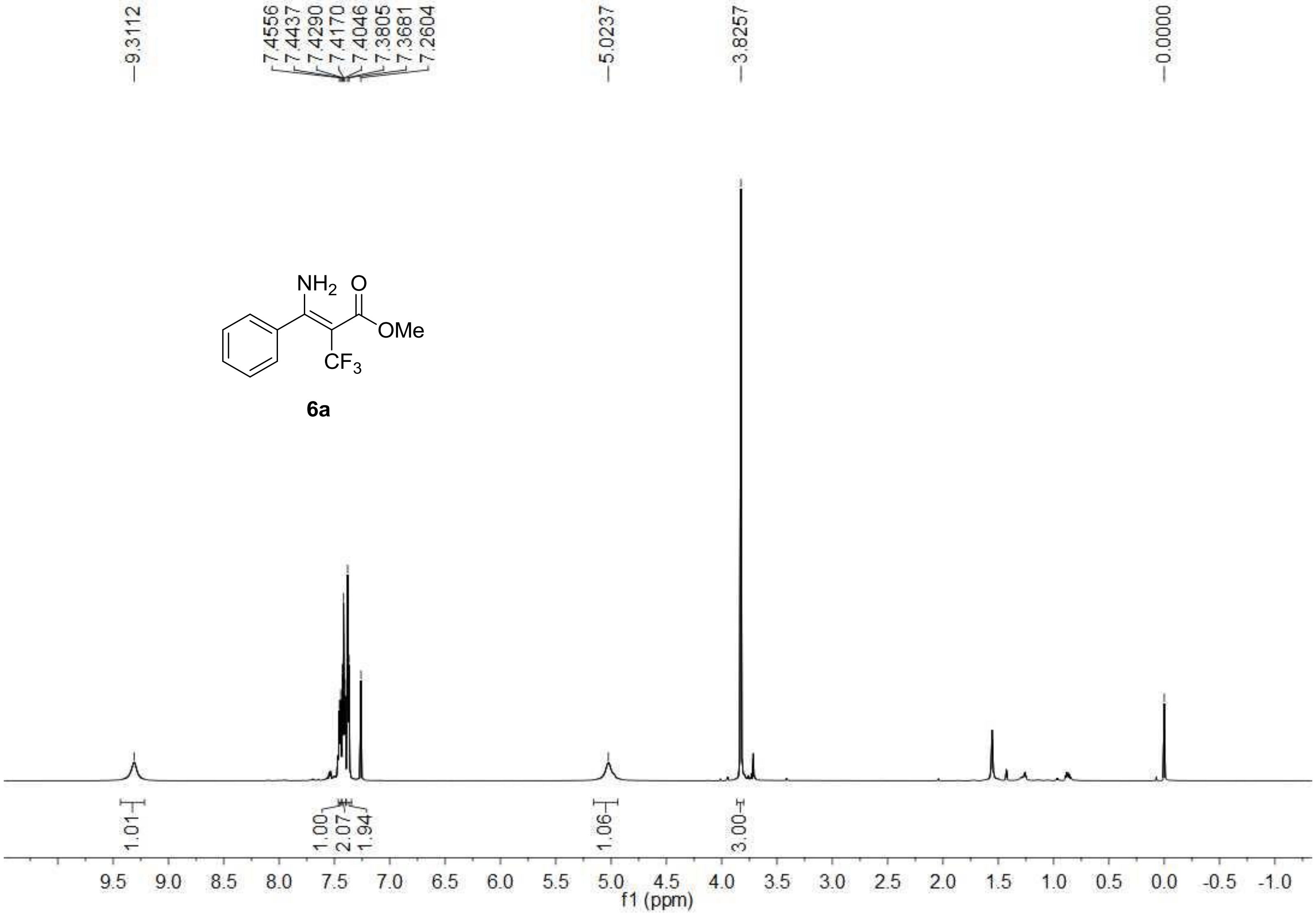
9.3112
7.4556
7.4437
7.4290
7.4170
7.4046
7.3805
7.3681
7.2604
5.0237
3.8257
0.0000
NH2
O
OMe
CF3
6a
1.01
1.00
2.07
1.94
1.06
3.00
9.5 9.0 8.5 8.0 7.5 7.0 6.5 6.0 5.5 5.0 4.5 4.0 3.5 3.0 2.5 2.0 1.5 1.0 0.5 0.0 -0.5 -1.0
f1 (ppm)

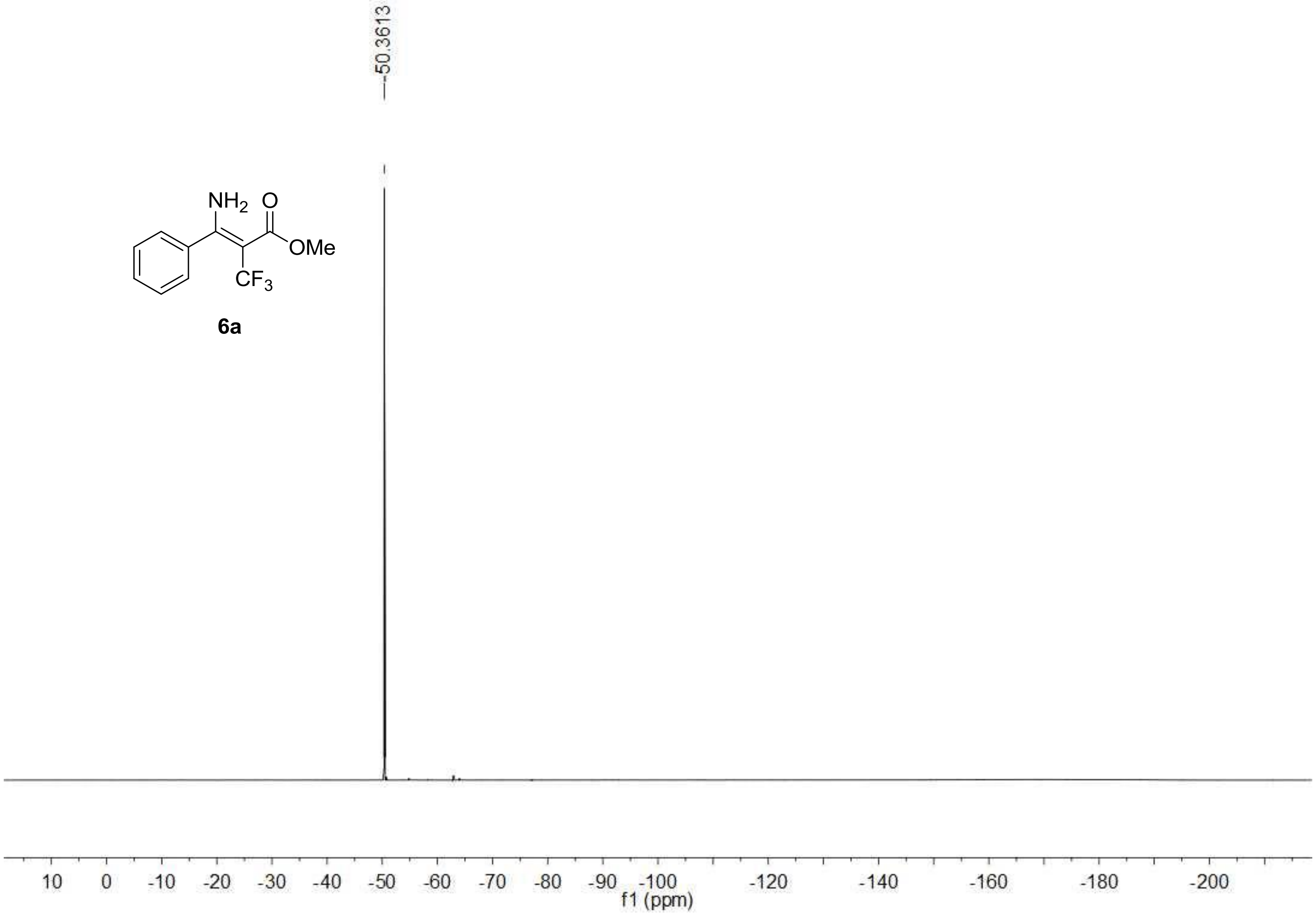

-50.3613
NH2
O
OMe
CF3
10
0
-10
-20
-30
-40
-50
-60
-70
-80
-90
-100
-120
-140
-160
-180
-200
f1 (ppm)

6a

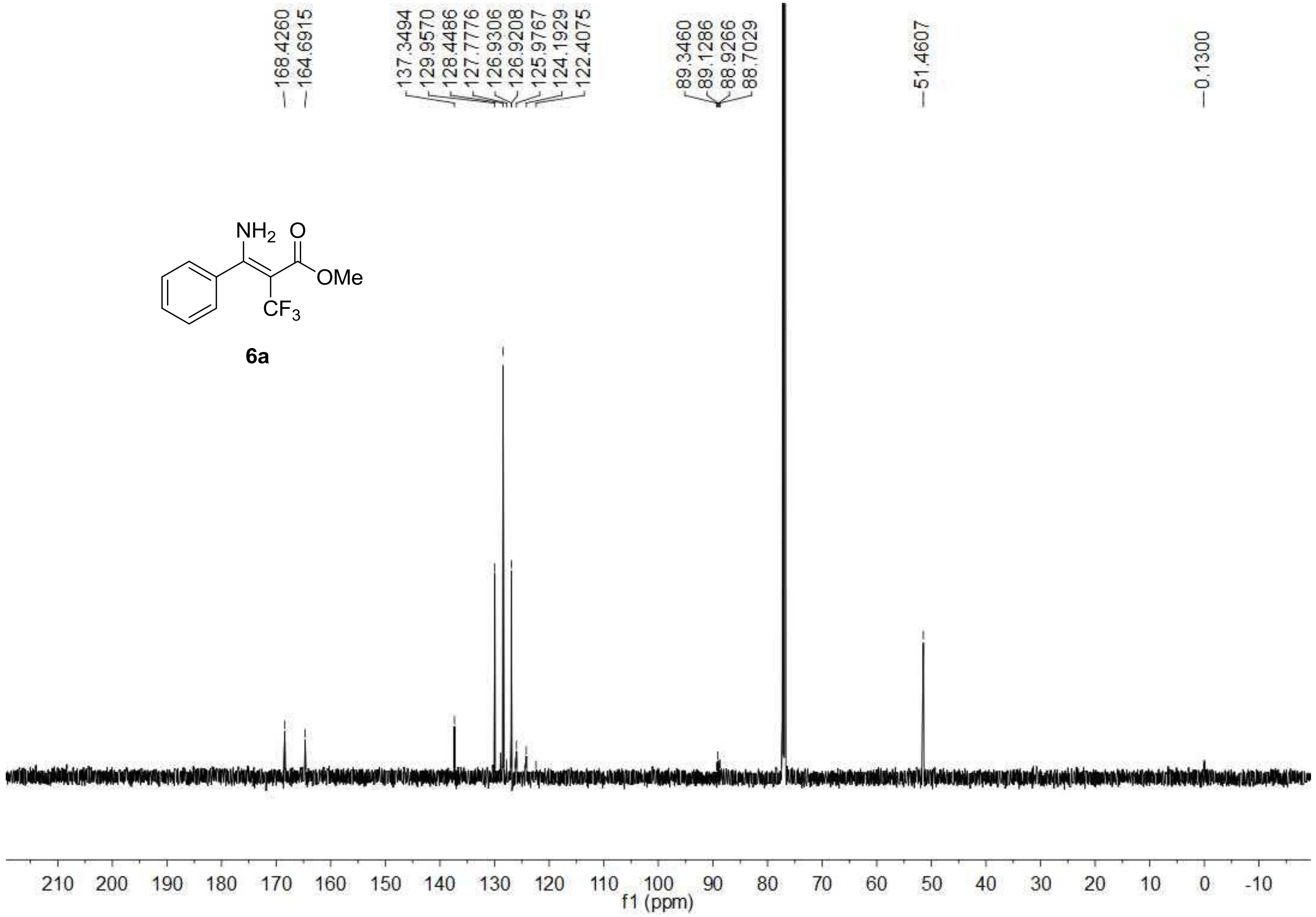
168.4260
164.6915
137.3494
129.9570
128.4486
127.7776
126.9306
126.9208
125.9767
124.1929
122.4075
89.3460
89.1286
88.9266
88.7029
51.4607
0.1300
NH2
O
OMe
CF3
6a
210 200 190 180 170 160 150 140 130 120 110 100 90 80 70 60 50 40 30 20 10 0 -10
f1 (ppm)

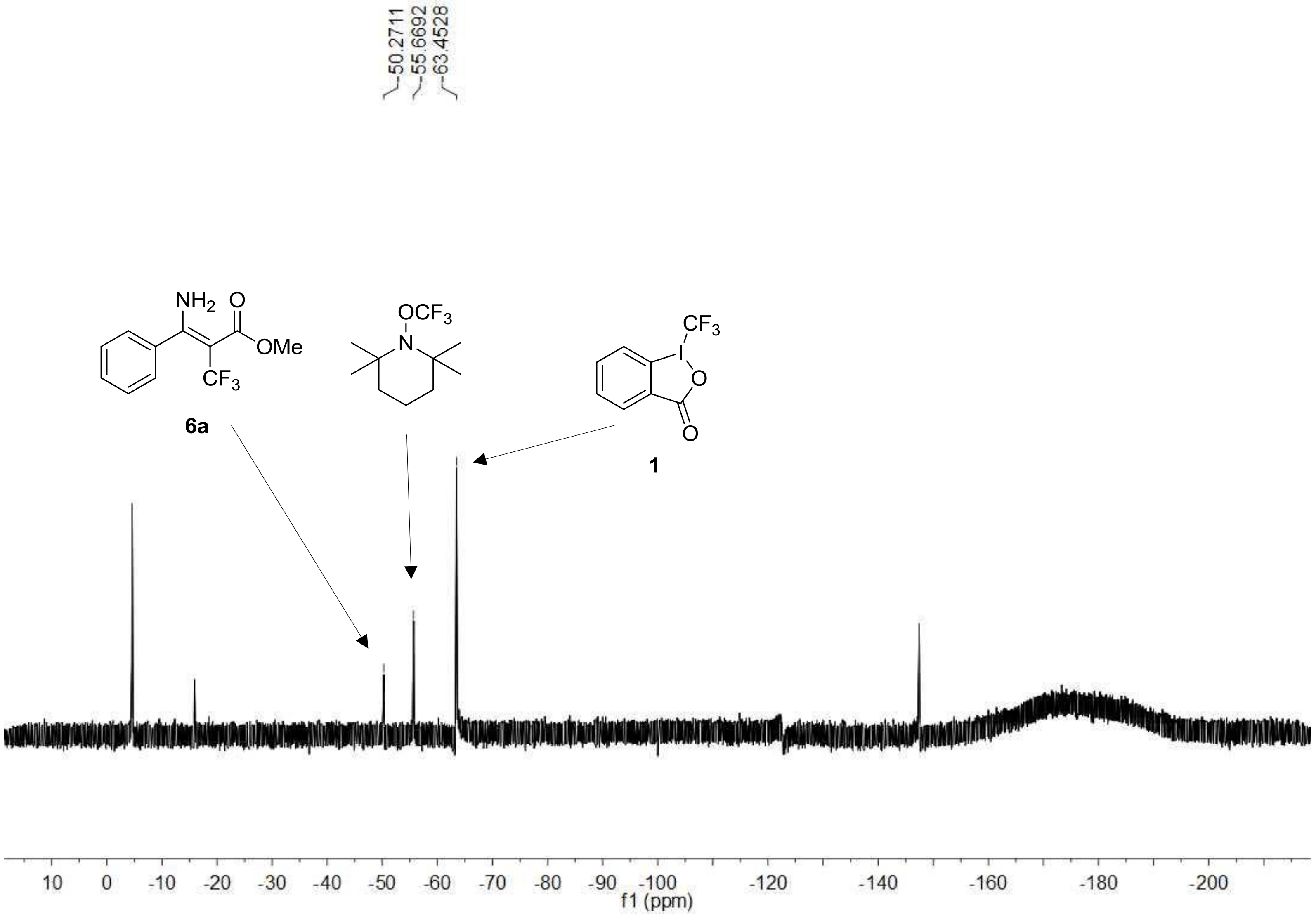

-50.2711
-55.6692
-63.4528
NH2
O
OMe
CF3
6a
OCF3
N
CF3
I
O
O
1
10 0 -10 -20 -30 -40 -50 -60 -70 -80 -90 -100 -120 -140 -160 -180 -200
f1 (ppm)
